# Unveiling a key catalytic pocket for the ruthenium NHC-catalysed asymmetric heteroarene hydrogenation†

**DOI:** 10.1039/d1sc06409f

**Published:** 2021-12-20

**Authors:** Andrea Hamza, Daniel Moock, Christoph Schlepphorst, Jacob Schneidewind, Wolfgang Baumann, Frank Glorius

**Affiliations:** Institute of Organic Chemistry, Research Centre for Natural Sciences Magyar Tudósok Körútja 2 H-1117 Budapest Hungary hamza.andrea@ttk.hu; Organisch-Chemisches Institut, Westfälische Wilhelms-Universität Münster Corrensstrasse 40 48149 Münster Germany glorius@uni-muenster.de; Leibniz-Institut für Katalyse e.V. Albert-Einstein-Straße 29a 18059 Rostock Germany; Institut für Technische und Makromolekulare Chemie, RWTH Aachen University Worringerweg 2 52074 Aachen Germany

## Abstract

The chiral ruthenium(ii)bis-SINpEt complex is a versatile and powerful catalyst for the hydrogenation of a broad range of heteroarenes. This study aims to provide understanding of the active form of this privileged catalyst as well as the reaction mechanism, and to identify the factors which control enantioselectivity. To this end we used computational methods and *in situ* NMR spectroscopy to study the hydrogenation of 2-methylbenzofuran promoted by this system. The high flexibility and conformational freedom of the carbene ligands in this complex lead to the formation of a chiral pocket interacting with the substrate in a “lock-and-key” fashion. The non-covalent stabilization of the substrate in this particular pocket is an exclusive feature of the major enantiomeric pathway and is preserved throughout the mechanism. Substrate coordination leading to the minor enantiomer inside this pocket is inhibited by steric repulsion. Rather, the catalyst exhibits a “flat” interaction surface with the substrate in the minor enantiomer pathway. We probe this concept by computing transition states of the rate determining step of this reaction for a series of different substrates. Our findings open up a new approach for the rational design of chiral catalysts.

## Abstract 2

DFT computations and *in situ* NMR spectroscopy studies on the mode of enantioinduction of the Ru-bis-NHC catalytic system in heteroarene hydrogenation indicate that the catalytic pocket and stabilizing non-covalent interactions play a key role.

## Graphical abstract

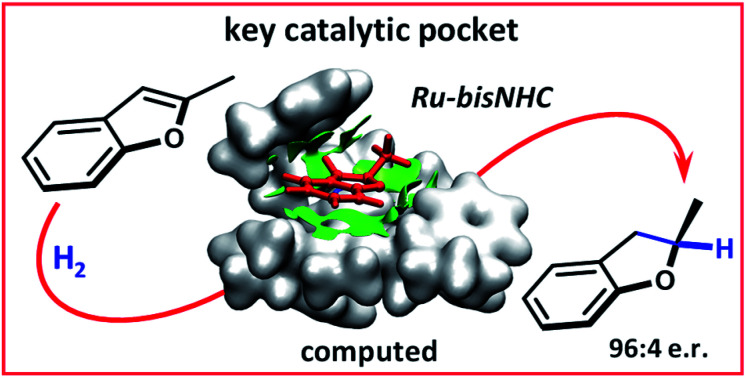

## Introduction

Enantioselective hydrogenation is arguably the most straightforward method for the conversion of aromatic to enantioenriched saturated heterocycles, thereby having high potential to promote a much needed expansion of the accessible chemical space.^1^ Although an increased interest in the area of enantioselective heteroarene hydrogenation has emerged in academia and industry in recent years,^2^ concurrently ensuring catalytic activity and selectivity still remains a particularly challenging task. As a consequence, asymmetric (hetero)arene hydrogenation continues to be considerably less explored compared to the hydrogenation of ketones, imines/enamines, and olefins.^3^ In comparison, heteroarene hydrogenation protocols often lack a detailed mechanistic understanding, especially regarding the mode of enantioinduction.^4^ This might be due to missing directed, polarized interactions between the catalyst (including its ligands) and the substrates in many successful catalyst systems, rendering computations more difficult and the identification of origins of stereoselectivity elusive. Non-covalent molecular (van-der-Waals) forces, especially attractive London dispersion forces, play a major role in molecular stabilization.^5^ Repulsive steric forces often predominate enantioinduction, while modes based on attractive London dispersion forces continue to be uncommon.^6^ An analysis of the contribution of such attractive dispersion to the stabilization of a transition state in a metal complex catalysed reaction is a truly needed addition to the field, as an improved understanding bears potential for the improvement of existing or the design of new, enzyme-like catalysts. We propose that especially the field of asymmetric heteroarene hydrogenation can profit of such exploration as π-systems and resulting saturated cycles are predestined motifs for non-directed forces.^7^

**Fig. 1:**
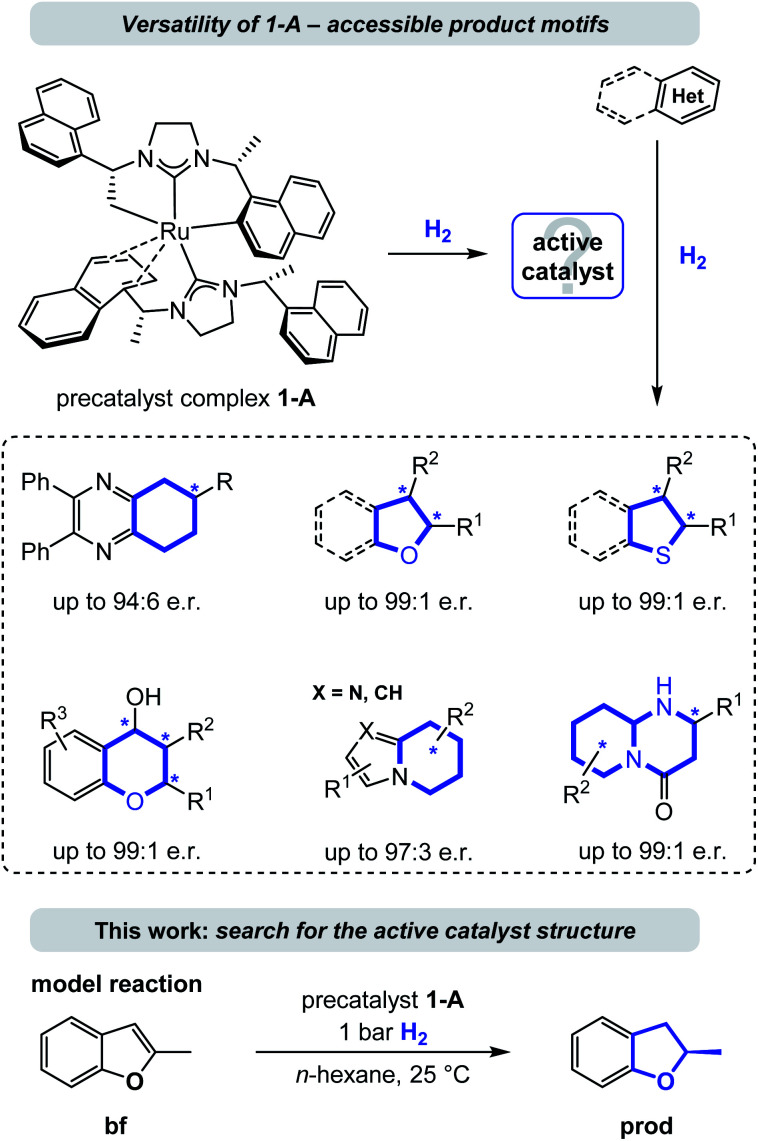
Explored scope of the privileged catalyst Ru-bis-NHC (1-A) for the enantioselective hydrogenation of various heteroarenes and the model reaction studied in this work. Abbreviation: e.r. = enantiomeric ratio.

Several homogenous catalysts have been developed for the saturation of heteroarenes by asymmetric hydrogenation^8^ and recent examples include complexes of iridium, rhodium, palladium, and ruthenium among other metals bearing a variety of chiral ligands.^9,10^ Because of their electron donating properties and structural modularity N-heterocyclic carbenes (NHCs) evolved to be a popular choice for the design of such chiral catalyst complexes.^11^ Particularly, the Ru-bis-NHC complex 1-A^10*a*,11*g*,12^ has emerged as a privileged catalyst (precursor) that is versatile and powerful in the asymmetric hydrogenation of a broad range of heterocycles including chromones and flavones,^10*b*^ the carbocyclic ring of quinoxalines,^10*a*^ (benzo)furans,^10*c*–*e*^ (benzo)thiophenes,^10*f*^ indolizines,^10*g*^ 2-pyridones,^10*h*^ cyclic vinylthioethers,^10*i*^ imidazo-pyridines,^10*j*^ 2-oxazolones,^10*k*^ and most recently pyrido-pyrimidones.^10*l*^ This ruthenium(ii) complex contains two chiral (*R*,*R*)-1,3-bis(1-(naphthalene-1-yl)ethyl)-4,5-dihydroimidazolylidene (SINpEt) ligands that have proven to be privileged structures with unique activity and selectivity. The same NHC-ligand could further be employed in a heteroleptic ruthenium complex together with a chiral diamine, granting access to additional enantioenriched, saturated heterocycles by hydrogenation.^10*m*,*n*^ In the precatalyst structure 1-A one of the two SINpEt ligands is doubly deprotonated and both bind in a multidentate fashion (see Fig. 1). In a hydrogen atmosphere this complex forms a catalytically active species which was observed to effectively hydrogenate structurally and electronically diverse heterocycles in a broad temperature range from −10 °C to 70 °C in various polar, apolar, protic as well as aprotic solvents.^10*a*–*l*^ Hydrogen pressure can be as low as 1 bar in the case of the hydrogenation of benzofurans, but typically ranges between 10 and 120 bar.^12^ Despite the broad synthetic utility and thorough characterization of structure and properties of the precatalytic Ru-bis-NHC 1-A complex, information on the structure of the catalytically active species under hydrogenation conditions as well as a deeper understanding of the underlying mechanism has been lacking.^12^

This work aims at the elucidation of the catalytically active form of 1-A by an exhaustive computational analysis and nuclear magnetic resonance (NMR) experiments. For this purpose, we investigated the hydrogenation of 2-methylbenzofuran (bf, Fig. 1), which was observed experimentally in previous work.^10*c*,12^

Herein we investigate in detail the vast conformational and configurational space of 1-A and derivatives to determine the identity of the active catalyst structure. Density Functional Theory (DFT) calculations have been carried out to provide a detailed analysis of several reaction pathways for the hydrogenation towards both product enantiomers. Based on the obtained results we analysed the stereoselectivity-determining factors driven by attractive dispersion forces in the catalytic pocket. These theoretical studies are supported by experimental observation of catalytic intermediates using *in situ* NMR spectroscopy. To the best of our knowledge, this is the first computational work providing insight into the mechanism of asymmetric heteroarene hydrogenation catalysed by Ru-bis-NHC 1-A.

## Results and discussion

### Computational details

For each individual complex and stationary point of the proposed reaction mechanism conformational search has been performed by Monte Carlo simulations using the built in OPLS_2005 force field of the program package MacroModel.^13^ A number of 10–40 conformers were selected for each molecule and optimized by applying DFT. The same procedure was followed for all the stationary points of the proposed reaction pathways, *i.e.* reaction intermediates and transition states as well (for details, see Section S1 of ESI†).

Estimating stereoselectivity by computation is a challenging task even for simpler systems. The most advanced levels of theory feasible for this catalytic system were used in order to ensure the best attainable accuracy. DFT calculations were performed by using the range-separated ωB97X-D exchange-correlational functional with dispersion corrections inherently included.^14^ For optimizations the SDD core potential^15^ with the respective basis set extended with additional polarization (*f*) functions^16^ was employed for the Ru atom and the 6-31G(d,p) basis set^17^ for the lighter atoms. To increase accuracy, for each optimized structure additional single point calculations were performed with a larger basis set, namely SDD augmented with 2f and 1g functions^16^ and the 6-311++G(2d,p) for the lighter atoms.^17^ The Gaussian 09 program package was used for all DFT calculations.^18^

For each optimized structure normal mode analysis was performed to characterize the nature of the stationary point as minimum or transition state. Thermal and entropic contributions to the gas-phase Gibbs free energy were estimated using the ideal gas, rigid rotor, harmonic oscillator (RRHO) model for *T* = 298.12 K and *c* = 1 mol L^−1^. Solvent effects were considered by computing the solvation free energies in the framework of integral equation formalism of the polarizable continuum model (SMD/IEFPCM) for *n*-hexane and toluene.^19^ The energetics presented here are relative Gibbs free energies including all the corrections calculated with the smaller basis set and the electronic energies at the large basis set. Reaction intermediates were localized by IRC calculations initiated from transition states in forward and reverse directions along the reaction coordinates, respectively. If otherwise not specified, rapid exchange is presumed between the different structures obtained by conformer screening. Therefore, each stationary point of the presented reaction pathways corresponds to the most stable conformer. The relevant structures were re-optimized by using other different levels of DFT in order to assess the reliability of the calculations (see Section S17 of ESI†).

### Summary of previous findings

**Fig. 2:**
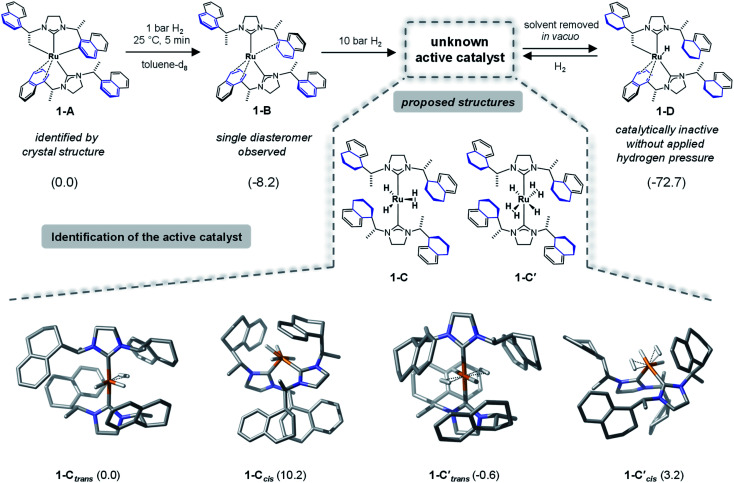
Lewis structures of experimentally identified precatalyst complexes 1-A, 1-B and 1-D and proposed catalyst structures 1-C, 1-C′ are shown in the upper part of the figure. Computed geometries for the possible isomers of 1-C and 1-C′ are displayed at the bottom of the figure. Relative Gibbs free energies in kcal mol^−1^ are shown in parenthesis, with respect to complex 1-A for the precatalyst structures and to complex 1-C*_trans_* for the forms of active catalyst, respectively. Structure 1-C*_trans_* is more stable than 1-D by 19.5 kcal mol^−1^ in free energy. The nomenclature 1-C*_trans_* refers to NHC ligands in *trans* positions. CH hydrogen atoms are omitted for clarity. Please note that while we show the absolute configuration of the newly formed stereocentres in the computed structures of 1-C, we do not indicate those in the Lewis formulas. In this study we were not able to characterise this stereochemistry without doubt.

To identify the structure of the active catalyst several factors have to be considered. Different coordination modes of the SINpEt ligand to the ruthenium centre are possible and for each configuration different molecular conformers may arise. This leads to a rather complex conformational space to be explored. The chiral Ru-bis-NHC 1-A system has been experimentally investigated in detail by Neugebauer, Wolf, Glorius *et al.*^12^ According to this study the structure of precatalyst [Ru(SINpEt)(SINpEt′)] 1-A is a rare example of a doubly cyclometalated NHC complex. The SINpEt ligand binds to the metal centre *via* the carbene C atom and the naphthyl ring in η^4^-coordination mode. By deprotonation of the methyl and naphthyl groups, the second SINpEt′ ligand coordinates to the ruthenium in a tridentate fashion. Upon application of low hydrogen pressure to a solution of 1-A in toluene-d_8_ the *cis* Ru(0)-bis-NHC complex 1-B is formed (Fig. 2). During the transformation of 1-A to 1-B the hydrogenation of 2-methylbenzofuran has been monitored, but no substrate coordination to the metal centre has been observed. Instead, the active, yet not identified form (1-C) of the catalyst is slowly formed under H_2_ pressure, presumably by partial hydrogenation of the naphthyl moieties. In an attempt to isolate 1-C after application of high hydrogen pressure and solvent evaporation *in vacuo*1-D was obtained. This complex is characterized by three partially hydrogenated naphthyl moieties, a methylene group bound to the metal centre and the η^4^-coordination of the fourth naphthyl group. Upon substrate addition to 1-D no hydrogenation of the 2-methylbenzofuran took place. However, after hydrogen gas was applied rapid hydrogenation of the benzofuran was observed and 1-D was no longer present. Instead, complex 1-C was again detected by NMR, indicating that this fourth partial naphthyl hydrogenation is reversible. Overall, the hydrogenation of naphthyl substituents appears crucial for the formation of the active catalyst.

### Forms of the active catalyst

As a starting point we initiated a detailed computational analysis of the structures of the experimentally observed species precatalyst 1-A, and unreactive species 1-B and 1-D (for comparison of calculated and experimental structure of 1-A see the ESI, Section S2†). The calculations for complex 1-B indicate that the protonation of the methylene group in 1-A results in a stabilization of 8.2 kcal mol^−1^. Further H_2_ uptake induces the partial hydrogenation of three naphthyl residues and additional stabilization (by 72.2 kcal mol^−1^) in complex 1-D, relative to 1-A. This is in good agreement with the experiments and given that 1-D is readily converted to the active catalyst under hydrogen atmosphere suggests that the active catalyst form also contains partially reduced naphthyl moieties. Under several possible transformations we assume the partial hydrogenation of the fourth naphthyl moiety to be the one most likely to lead to the active catalyst form of 1. Out of the plethora of identified conformers only the most stable structures of 1-C are presented in Fig. 2 (several energetically higher lying conformers can be found in the ESI, Sections S5 and S6†). In 1-C*_trans_*, one dihydrogen and two hydride ligands are bound to the ruthenium centre and its relative energy is set to 0.0 kcal mol^−1^ and it is 19.5 kcal mol^−1^ more stable than 1-D. However, several ruthenium hydride/dihydrogen species might exist in dynamic exchange when H_2_ pressure is applied. Structure 1-C′*_trans_* contains two dihydrogen and two hydride ligands and has an energy of −0.6 kcal mol^−1^ relative to 1-C (Fig. 2).^12^ In this nomenclature “*trans*” and “*cis*” notations refer to the arrangement of carbene ligands. Computed structures of other forms of the catalysts (*e.g.* with *cis* coordination of the carbene ligands) proved to be too high in energy or inactive for the present reaction (see Fig. 2 and Sections S4, S9 and S10 in the ESI†). Thermodynamically, 1-C′*_trans_* is the lowest lying structure. However, a vacant substrate coordination site is required for successful hydrogenation. Since substrate coordination to 1-C′*_trans_* is not possible without the dissociation of one H_2_ molecule, the coordinated complex bf⋯1-C*_trans_* was chosen as the starting point of the reaction. The *cis* analogue of 1-C*_trans_* was also considered in a detailed analysis but proved to lead to non-feasible reaction pathways (see the details in Sections S7 and S8 of the ESI†).

### NMR characterization of 1-C

**Fig. 3:**
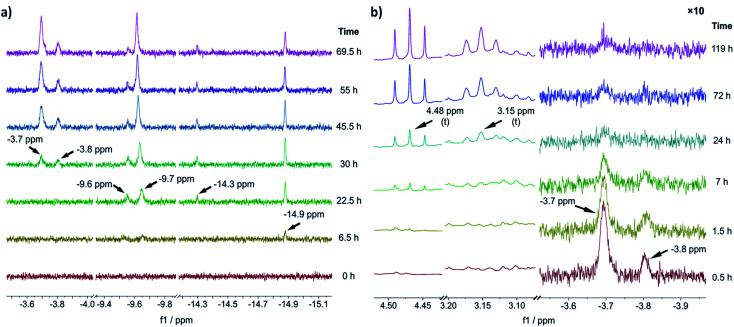
(a) Slices of the hydride region of ^1^H NMR spectra recorded before (*t* = 0 h) and after addition of H_2_ to a solution of 1-A at different reaction times. (b) Slices of aliphatic (left) and hydride (right) region of ^1^H NMR spectra recorded after addition of benzofuran (at *t* = 0 h, 1.5 equiv. relative to 1-A) to the reaction solution obtained *via* reaction of 1-A with H_2_ for 69.5 h. The hydride region has been scaled by a factor of 10 relative to the aliphatic region. All experiments were performed in THF-d_8_, for details see ESI Section S19†.

To gain an experimental perspective on the nature of the active catalyst species, we studied the reactivity of 1-A using *in situ* NMR spectroscopy. First, we investigated the speciation of 1-A over time under a 1 bar H_2_ atmosphere, with the results shown in Fig. 3a. It is important to note that due to relatively poor mixing and diffusion inside the NMR tube (compared to a stirred reactor), all reactions are expected to occur at timescales slower than those observed in normal reactors. 6.5 h after addition of H_2_, the first hydride species could be observed at *δ*(^1^H) = −14.9 ppm, followed by the appearance of new hydride signals at *δ*(^1^H) = −9.6, −9.7 and −14.3 ppm after 22.5 h. Likely, corresponding large and small signals (*δ*(^1^H) = −14.9, −14.3 and *δ*(^1^H) = −9.7, −9.6 ppm) are major and minor isomers of the same species.

The chemical shift of *δ*(^1^H) = −9.7 ppm is in good agreement with the previously identified monohydride species 1-D, in which three out of four naphthyl substituents have been partially hydrogenated.^12^ A relatively slow hydrogenation of the naphthyl ring could also explain why this species only appeared after 22.5 h of reaction time. The earlier appearance of the species at *δ*(^1^H) = −14.9 ppm could in turn suggest that it is an intermediate formed on route to 1-D.

After 30 h of reaction time, an additional pair of hydride signals (also likely major/minor isomers) appeared at *δ*(^1^H) = −3.7 and −3.8 ppm. Given that this species only appeared after formation of 1-D suggests that it might be derived from it, possibly *via* partial hydrogenation of the fourth naphthyl substituent. The strong downfield shift of the hydride resonance is characteristic for a *trans*-dihydride complex, due to the strong *trans* effect of hydride ligands.^9*c*^ Furthermore, the chemical shift of the hydride ligands is in good agreement with the predicted shift for 1-C′*_trans_* (between *δ*(^1^H) = −3.4 and −3.8 ppm) (see ESI, Section S20†). Hence, we propose that this species is a *trans*-dihydride complex with four partially hydrogenated naphthyl substituents akin to 1-C′*_trans_*. Since the reaction was conducted in THF-d_8_, the two equatorial ligands could be either H_2_ or THF molecules. To investigate the kinetic competence of the formed hydride complexes for heteroarene hydrogenation, we added benzofuran to the reaction mixture under 1 bar of H_2_ and continued *in situ* NMR monitoring (see Fig. 3b). Benzofuran instead of 2-methylbenzofuran was used to resolve product signals more easily due to the lower multiplicity of the aliphatic protons. The addition of benzofuran led to gradual reduction of the hydride signal intensity (but not complete disappearance) at *δ*(^1^H) = −3.7 and −3.8 ppm and concomitant formation of two triplets at *δ*(^1^H) = 4.48 and 3.15 ppm, which correspond to the aliphatic protons in 2,3-dihydrobenzofuran (see Fig. 3b). Hydride signals for the other species either remained constant in intensity or slightly increased and no new hydride species appeared (see ESI, Fig. S25†). These results suggest that the *trans*-dihydride species *δ*(^1^H) = −3.7 ppm is reactive towards benzofuran. It might form a substrate complex, allowing for hydride transfer and subsequent product formation (see ESI, Fig. S25 and S26†). The fact that its concentration is reduced without completely disappearing indicates that presence of benzofuran leads to a new steady-state concentration for this species, possibly due to its involvement in the catalytic cycle. Slow formation of the *trans*-dihydride species is also in agreement with the catalytic reaction's pronounced induction period.^12^

Based on these *in situ* NMR studies we conclude that slow, partial hydrogenation of the naphthyl substituents leads to the formation of a *trans*-dihydride species (*δ*(^1^H) = −3.7 ppm), which is akin to 1-C′*_trans_* in structure and appears to be an intermediate in the catalytic cycle.

### Substrate coordination

**Scheme 1:**
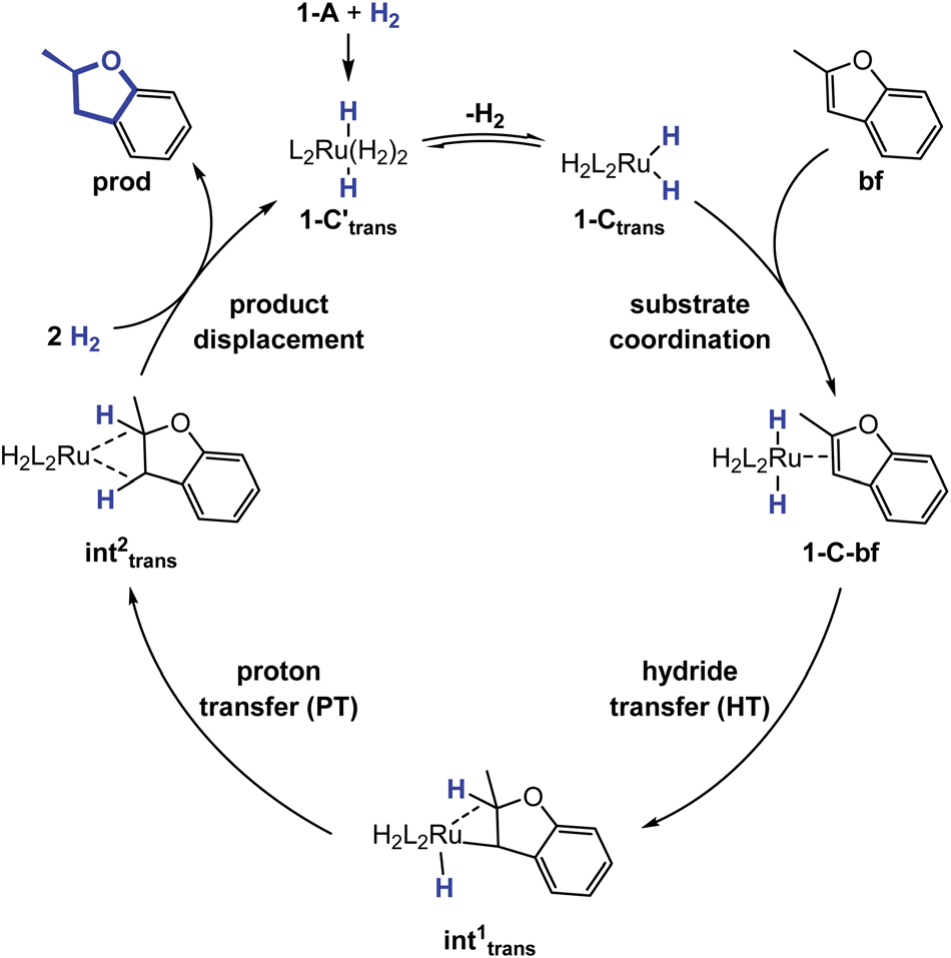
Proposed catalytic cycle of the heterolytic H_2_-cleavage hydrogenation reaction of bf.

The generalized reaction mechanism for asymmetric heteroarene hydrogenation requires the transfer of a hydride and a proton from the catalyst to the substrate (Scheme 1). As a starting point for our proposed mechanism we chose 1-C*′_trans_*, which is the most stable species in absence of a substrate. During substrate coordination we found a *cis*-dihydride substrate complex to be the most stable one (1-C-bf see ESI, Section S11† for energetic comparison of catalyst substrate complexes and corresponding transition states). Thus, we propose the formation of 1-C*_trans_* in equilibrium, which also exhibits the required vacant coordination site. After the association of bf, the next step involves a hydride transfer (HT) to the substrate with the corresponding barrier denoted TS^HT^. This leads to an intermediate where the η^2^-coordination of the substrate is preserved and the H atom is bound to both, the C2 atom of the substrate and to the metal centre *via* an agostic interaction. The second step is a proton transfer (PT) to the anionic carbon of the substrate. The catalyst 1-C′*_trans_* is regenerated as most stable species by oxidative addition of a new dihydrogen molecule (Scheme 1) and the association of a second dihydrogen. The stereochemical outcome of the reaction depends on the orientation of the substrate approaching the metal centre of the catalyst *via* its si- or re-face. The formation of the major (*R*)-product proceeds *via* the si-face attack.

**Fig. 4:**
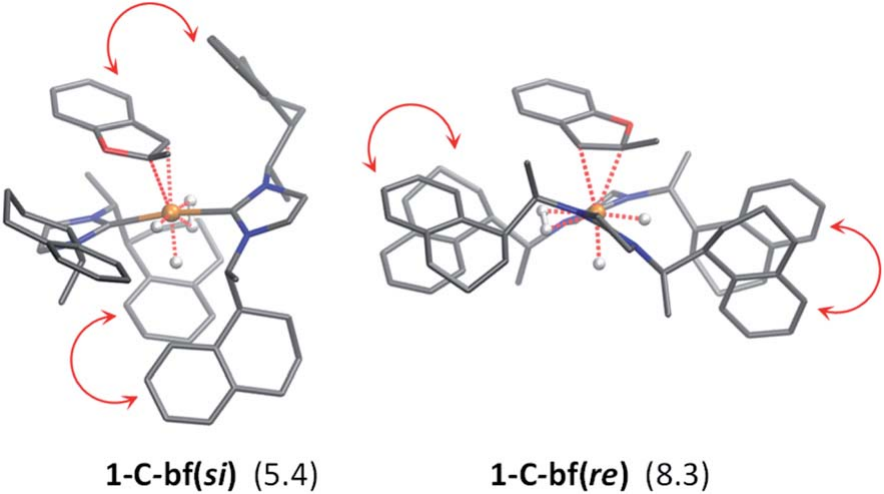
Optimized structures of 1-C-bf substrate complexes, in parentheses the Gibbs free energies (relative to 1-C*_trans_* + bf) are shown in kcal mol^−1^. Intramolecular π-stacking interactions are indicated by red arrows. The CH hydrogen atoms have been omitted for clarity.

In extensive computational studies for the catalyst⋯substrate intermediates all classes of catalyst 1-C were considered in detail with both, si- or re-face coordination of the bf. Herein, we exclusively present relevant structures, for details on all catalyst configurations studied see ESI, Sections S4–S7 and S9–S12.† All free energy values discussed here are related to the separated catalyst 1-C*_trans_* and bf. Catalyst 1-C*_trans_* affords the most preferred intermediate 1-C-bf(si) corresponding to the si-face coordination of the substrate, with a relative energy of 5.4 kcal mol^−1^. The re-face analogue 1-C-bf(re) is 2.9 kcal mol^−1^ higher in free energy (Fig. 4). Coordinated complexes corresponding to the 1-C*_cis_* catalyst isomer are more destabilized (see ESI, Section S10†). The same si-face coordination is also feasible for a substrate complex with *trans*-dihydride arrangement (akin to 1-C′*_trans_*), however our calculations predict that this structure lies 2.3 kcal mol^−1^ higher in energy. Overall the *cis*- and *trans*-dihydride isomers of 1-C*_trans_* are likely in fast equilibrium. We carried out a detailed study of the pathways with the *trans*-dihydride catalyst as well (see SI Section S12†), but we will focus on the energetically favourable *cis*-dihydride pathway.

The substrate binding mode is a common feature of all computed coordinated complexes. Coordination of bf involves η^2^ interaction of the C=C bond of the furan ring with the ruthenium centre. The Ru⋯C distances are relatively short (≈2.2 Å) and the 2-methylbenzofuran is non-planar; the H and methyl-groups of the furan ring point out of plane. However, the arrangement of the bulky naphthyl moieties is rather different in the computed structures. Complex 1-C-bf(re) exhibits pairs of aromatic stacking interactions of naphthyl moieties belonging to the different carbenes on the ruthenium centre (shown by red arrows in Fig. 4). The average distance between the carbon atoms of stacking naphthyl groups is 3.5 Å, pointing to stabilizing van-der-Waals interactions. In contrast, the catalyst ligands in structure 1-C-bf(si) form a well-defined binding pocket for the substrate. Several intra- and intermolecular contacts are noticed: T-shape like stacking of two adjacent naphthyl groups and secondary interactions between a naphthyl substituent of the catalyst and the aromatic part of the coordinated bf.

These two catalyst conformations were compared by removing the substrate of the coordinated complexes and re-optimizing the catalysts. These computations indicate that the catalyst structure originating from 1-C-bf(si) preserves the binding pocket while the more planar catalyst isomer of 1-C-bf(re) remains plain and is 5.2 kcal mol^−1^ higher in energy. Thus, the energy difference of the coordinated complexes partially originates in the preferred conformer structure of the ruthenium complex (see Section S13 of ESI†).

### On the origin of stereoselectivity

**Fig. 5:**
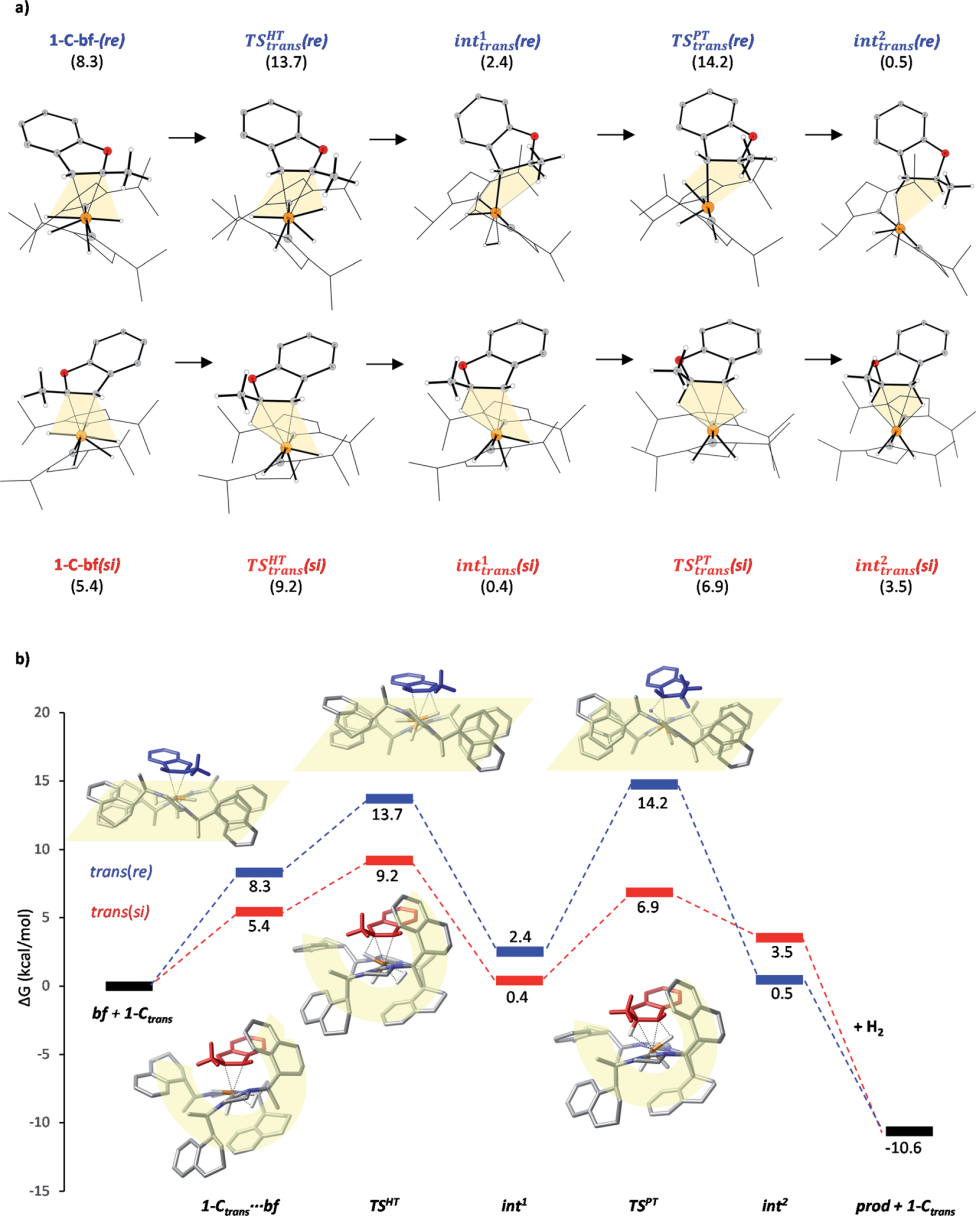
(a) Calculated structures along pathways *trans*(si) (lower) and *trans*(re) (upper). Gibbs free energies shown in parenthesis (in kcal mol^−1^) refer to the separated species 1-C*_trans_* and bf. (b) Reaction pathways *trans*(si) and *trans*(re). Gibbs free energy values refer to the separated reactants in kcal mol^−1^. Selected structures of coordinated complexes, HT and PT transition states are shown. The yellow shapes highlight the arrangements of NHC ligands.

The studied hydrogenation proceeds *via* a heterolytic H_2_-cleavage mechanism. We chose the si-face coordination of bf to the active catalyst form 1-C*_trans_* as the starting point (1-C-bf(si)) of the reaction, ultimately leading to the (*R*)-product isomer. The minor product is formed by following pathway *trans*(re). The calculated elementary steps along the two pathways are depicted in Fig. 5a. The re counterpart of complex 1-C-bf(si) is higher in energy but both structures are analogously prepared for the hydride- and the subsequent proton transfer processes (highlighted in yellow). Complexes 1-C-bf(si) and int^1^*_trans_*(si) are of a very similar structure. Complex int^1^*_trans_*(re), however, shows a slightly shifted bf ligand with a less pronounced agostic interaction. We found this important structural difference to be conserved all along the two pathways. The second reaction step involves a proton transfer from the ruthenium centre to the C3 atom of bf. The transition state for the re-complex is predicted to be 7 kcal mol^−1^ above TS^RE^*_trans_*(si). In the resulting intermediates int^2^*_trans_* of both pathways the dihydrobenzofuran remains bound *via* one or two agostic interactions to the ruthenium centre of the catalyst. Product elimination takes place simultaneously with the association of a new H_2_ to the free coordination site of the catalyst.

According to earlier experimental results the asymmetric hydrogenation of 2-methylbenzofuran selectively leads to the (*R*)-configured product with 96 : 4 e.r., which corresponds to a Gibbs free energy barrier difference of Δ*G* = 1.88 kcal mol^−1^ for the rate determining step. Our calculations indicate that pathway *trans*(si) toward the major product *R* is favoured over pathway *trans*(re) and the selectivity determining step is the hydride transfer. The energy difference between the HT barriers on the two pathways is 4.5 kcal mol^−1^ which is in qualitative agreement – within the expected calculation errors – with the experimental selectivity. The calculated selectivity is slightly nuanced by the height of transition state TS^PT^*_trans_*(re), which is of comparable magnitude with TS^HT^*_trans_*(re). Although the protonation transition state is predicted to be slightly above the hydride transfer TS on the minor pathway, *i.e.* it represents the rate determining state in this case, the above stereoselectivity model remains valid.

**Fig. 6:**
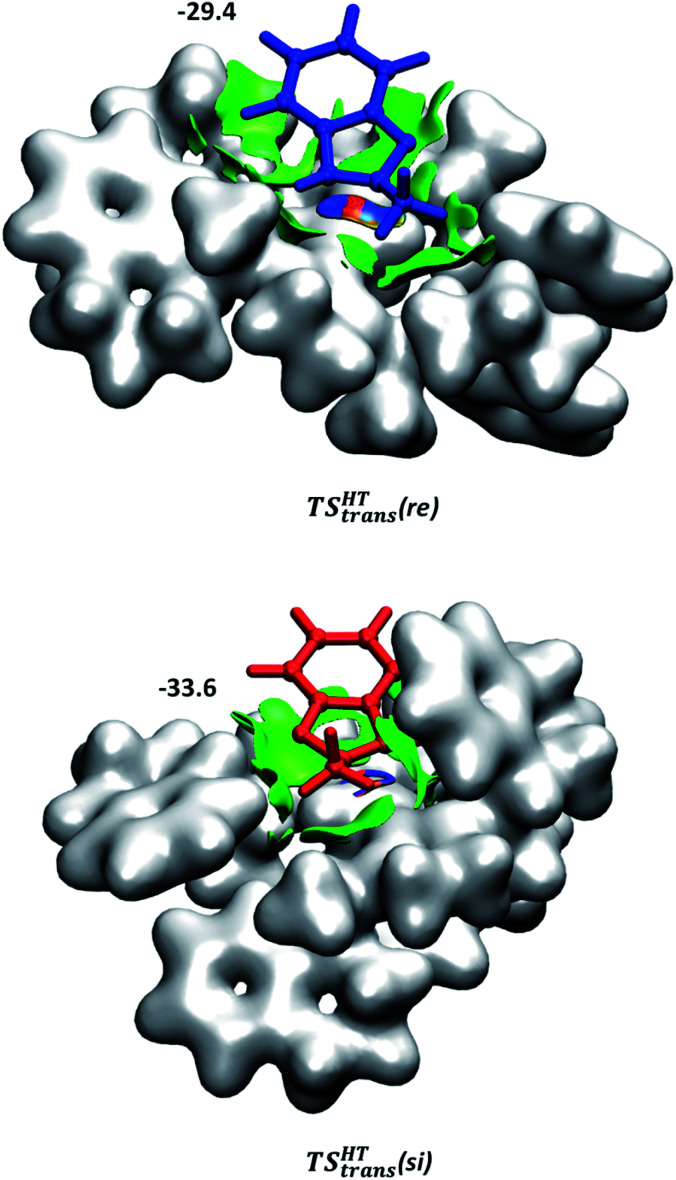
NCI plots generated for TS^HT^*_trans_*(si) and TS^HT^*_trans_*(re). Weak non-covalent interactions are represented in green. The applied cut-off for the gradient is *s* = 0.3 a.u. The catalyst is represented by an isodensity surface with *ρ* = 0.01 a.u. The numbers are the computed dispersion contributions to the total interaction energies calculated by using the D4 method^20^ (in kcal mol^−1^). For details see Section S15 of ESI.†

Although the first coordination sphere around the ruthenium centre is structurally very similar in both pathways, the arrangement of the naphthyl moieties exhibits considerable differences. The chiral pocket formed by the naphthyl moieties in complex 1-C-bf(si) is preserved along the *trans*(si) pathway (highlighted by yellow shapes in Fig. 5b). In contrast, along the pathway towards the minor product isomer the “flat” ligand arrangement observed in complex 1-C-bf(re) is maintained. To gain insight into the role of these different types of contact surfaces we generated the reduced density gradient (RDG) isosurface for the transition states TS^HT^*_trans_*(si) and TS^HT^*_trans_*(re). The non-covalent interaction (NCI) plots obtained are shown in Fig. 6 where stabilizing van-der-Waals interactions are represented by the green areas. It is apparent that the naphthyl moieties in the chiral pocket of the si-face transition state provide an intermolecular interaction with the substrate stronger by 11.6 kcal mol^−1^ compared to the re-face transition state (see Section S15 of ESI†). Thus, the remarkable ligand flexibility and conformational freedom of the partially hydrogenated naphthyl moieties leads to a non-covalent “lock-and-key” role of the catalyst complex. Chiral binding pockets were recently found to play a key role in asymmetric imine hydrogenation under frustrated Lewis-pair catalysis^21^ and more recently were analysed computationally in detail for confined binaphthyl-allyl-tetrasulfone and imidodiphosphorimidate catalysts.^22^

To assess the role of the proposed binding pocket the re-analogue of 1-C-bf(si) was computed by preserving the chiral pocket environment of the catalyst (for details, see ESI, Section S14†). The results indicate that the re-face binding of the substrate into the pocket is hindered by steric repulsion between the methyl-group of bf and the naphthyl moiety forming the pocket (see Fig. S19 in the ESI†). Overall the calculations predict this structure at 16.2 kcal mol^−1^ in free energy. Thus, the only thermodynamically possible way to form the minor product isomer is *via* the re-face coordination to the “flat” catalyst conformer. The computational results imply that the stereoselectivity of the bf hydrogenation is foremost induced by the preference of the catalyst to form the pocket conformer over the flat conformer. The strong preference for the si-face substrate coordination of this chiral pocket conformer and the stabilization by attractive dispersion interactions explain the high enantioselectivity of the studied hydrogenation.

### Hydride transfer transition states for different substrates

**Fig. 7:**
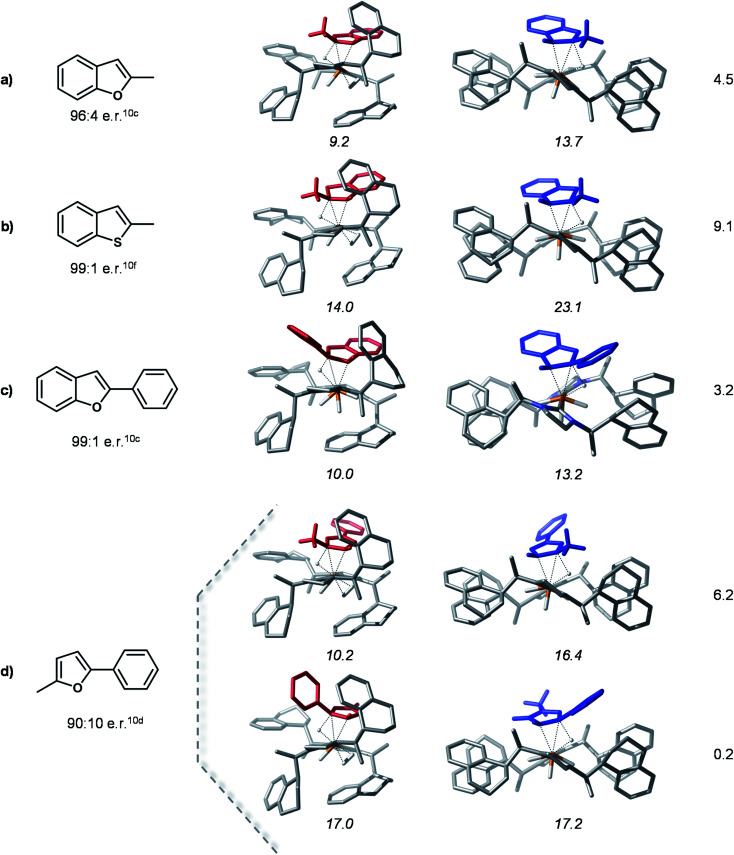
Computed HT transition state structures for different substrates. Experimental e.r. of hydrogenated products are shown below the Lewis structures of the substrates. The numbers in italics are the Gibbs free energies in kcal mol^−1^ of complexes with respect to the separated species 1-C*_trans_* and substrate. The numbers in the right column are the relative stabilities of the major and minor transition states (in kcal mol^−1^).

In order to probe the proposed stereoselectivity model we inspected the transition states of the rate determining step for this reaction for a series of different substrates with available experimental data. The TS^HT^*_trans_*(si) and TS^HT^*_trans_*(re) stationary points were used as model structures for the “pocket” and “flat” forms of these complexes. The investigated substrates cover a larger scale of steric and electronic properties and the corresponding products are obtained in high enantioselectivities. The compounds considered for this analysis and the optimized transition states structures are shown in Fig. 7.

The computed model HT transition states show that all considered substrates bind into the chiral pocket of the catalyst analogously to bf and the form of the catalyst remains almost unaltered. In all cases the transition states corresponding to the “flat” catalyst conformer are thermodynamically less favoured, thus leading to the minor product isomer. The separation of the transition states on the minor and major pathways in free energy is rather large, indicating high stereoselectivities. For 2-methyl-5-benzofuran (entry 5 in Fig. 7) coordination *via* two different double bonds and hence, two asymmetric reductions are possible. The computed energies of the HT transition states indicate that coordination *via* the alkyl substituted double bond takes place first, which is then also reduced first. These results are in qualitative agreement with experimental findings. The relative stabilities of transition states might be further refined by detailed conformational search for each structure, however, this is out of the scope of the present analysis. Overall, the computations carried out for these different substrates and their agreement with experimental data corroborate the proposed selectivity model.

## Conclusions

In this work, we have presented a detailed computational study of the asymmetric hydrogenation of 2-methylbenzofuran (bf) promoted by the Ru-bis-NHC (1) catalytic system. A conformational and configurational analysis covered the manifold orientations of NHC ligands and accounted for Ru⋯C and Ru⋯H bond breaking and formation. Computations predicted that the most stable form of the catalyst is *trans*-dihydride complex 1-C′*_trans_*, which was observed experimentally using *in situ* NMR spectroscopy. After liberation of one coordination site this complex is competent for the hydrogenation of benzofuran, which was experimentally confirmed through reactivity studies. For all elementary steps of the proposed hydrogenation mechanism quantum chemical calculations as well as conformational searches were performed. The stereochemical reaction outcome is decided by the si- or re-face approach of the substrate and the hydride transfer was found as the rate determining step. In accordance with experimental findings, the calculated stereoselectivity predicts the *R* isomer as the major hydrogenation product. The computed structures reveal that the naphthyl-groups of the most stable catalyst conformer define a binding pocket and coordinated bf is stabilized by attractive non-covalent interactions in this chiral-pocket in all stationary points along the major pathway. The flexibility of the catalyst allows for a non-covalent “lock-and-key” function with attractive London dispersion as part of the significant forces. Due to steric hindrance the re-face coordination into the pocket environment is not favoured. Thus, the complexes following the minor pathway do not display the chiral-pocket form of the catalyst but only a “flat” interaction surface with the substrate.

Our computational analysis provides insight into the form of the active catalyst and asymmetric catalytic hydrogenation of arenes. Similar to a recent report,^22^ we found a balance of steric and attractive interactions to play a major role in the studied transformation. The findings on stereocontrolling factors support the recent drive that attractive noncovalent interactions and London dispersion forces should be used more deliberately as a catalyst design element in the future. With our findings we hope to spark a new approach to the rational development of such catalysts, for example by targeted modifications of polarizability or the size of parts of the identified chiral catalyst pocket. This work is currently ongoing in our laboratories.

## Data availability

The computational methods have been described in the section “Computational details” of the manuscript and “Computational methods” of ESI.† Cartesian coordinates of the most important calculated structures are provided at the end of ESI.† Original NMR data files have been made publicly available *via* Zenodo and can be found at: https://doi.org/10.5281/zenodo.5801976.

## Author contributions

A. H. performed all computations. A. H., F. G., D. M., and C. S. developed the project, D. M. and C. S. performed the synthetic experiments. W. B. and J. S. planned and performed the NMR experiments and analyzed the resulting data. F. G. supervised the research and A. H., D. M., J. S., and F. G. wrote the manuscript.

## Conflicts of interest

There are no conflicts to declare.

## Supplementary Material

SC-013-D1SC06409F-s001
