# Supplementary material for: Unveiling a key catalytic pocket for the ruthenium NHC-catalysed asymmetric heteroarene hydrogenation
Source: Chem Sci. 2021 Dec 20;13(4):985–95. doi: 10.1039/d1sc06409f (PMC8790799; doi:10.1039/d1sc06409f)
Supplement: SC-013-D1SC06409F-s001 [file SC-013-D1SC06409F-s001.pdf]

Electronic Supplementary Material (ESI) for Chemical Science.
This journal is © The Royal Society of Chemistry 2021

Supplementary Information

Unveiling a Key Catalytic Pocket for the Ruthenium NHC-Catalysed Asymmetric Heteroarene Hydrogenation

Andrea Hamza,*[a] Daniel Moock,[b] Christoph Schlepphorst,[b] Jacob Schneidewind,[c,d] Wolfgang Baumann,[c] and Frank Glorius*[b]

[a]*Institute of Organic Chemistry, Research Centre for Natural Sciences, Magyar tudósok körútja 2, H-1117 Budapest, Hungary*

[b]*Organisch-Chemisches Institut, Westfälische Wilhelms-Universität Münster, Corrensstrasse 40, 48149 Münster, Germany*

[c]*Leibniz-Institut für Katalyse e. V., Albert-Einstein-Strasse 29a, 18059 Rostock, Germany*

[d]*Institut für Technische und Makromolekulare Chemie, RWTH Aachen University, Worringerweg 2, 52074 Aachen, Germany*

*e-mail: hamza.andrea@ttk.hu glorius@uni-muenster.de

Table of Contents

S1. Computational methods S3

S2. Comparison of experimental and calculated structures of precatalyst **1-A** S3

S3. Energy data of the most stable conformers of **1-A**, **1-B** and **1-D** S4

S4. Catalyst form **1-F** S4

S5. Computed catalyst isomers S5

S6. Conformational space of **1-C$_{trans}$** S7

S7. Free energy diagrams of catalyst-substrate complexes for the isomers of **1-C** and cyclometalated **1-E** S8

S8. Coordination complexes S9

S8.1 Structures of coordination complexes **1-E$_{trans}$-bf(si)** and **1-E$_{trans}$-bf(re)** S9

S8.2 Other forms of coordination complexes **1-C$_{trans}$-bf** S10

S8.3 Solvent coordination to the catalyst S10

S9. Reaction pathways for catalyst form **1-C″** S11

S10. Comparison of pathways *cis/trans* S12

S11. Transition states corresponding to the *cis/trans*-transformation of catalyst **1-C** and coordinated complex **1-C-bf(si)** S14

S12. Reaction pathway for the *trans*-dihydride catalyst **1-C$_{trans}$-H$_2$*trans*** S15

S13. Stability of “pocket” and “flat” conformers of **1-C$_{trans}$** S18

S14. “Pocket” configuration for **1-C$_{trans}$-bf(re)** S19

S15. Interaction energies and dispersion interaction S21

S15.1 Computational details S21

S15.2 Total and dispersion interaction energies S21

S15.3 Truncated model of “pocket” complex S22

S15.4 Activation strain energy S24

S16. Computed structures for different substrates S26

S17. Energy data calculated by other methods S27

S18. Energy data of calculated structures for the elementary steps of the reaction pathway S28

S19. In Situ NMR Experiments S29

S19.1 Experimental Procedure S29

S19.2 Results S29

S20. Calculated NMR spectra S32

S21. Cartesian coordinates of the calculated structures S34

S1. Computational methods

The initial conformational search on all structures was performed by Monte Carlo simulations using the built in OPLS_2005 force field of the program package *MacroModel*.[13] The bonds between the Ru and C atom of carbene ligands were kept frozen during the conformational search for the catalyst forms. Additionally the two C atoms of the furan ring coordinated to the Ru centre were also fixed for the intermediates and transition state structures during the Monte Carlo simulations. For each ensemble of conformers a similarity check was performed and 10 up to 40 of the most different structures were collected and fully re-optimized at the DFT level of theory without any restrictions on Ru-C (or other) bonds. For several low lying TS conformers IRC calculations were performed and if necessary additional conformer search was performed for these new structures by following the procedure above.

The energies presented in this paper correspond to the solution phase Gibbs free energies computed according to equation (1):

$$G_{solv} = E^{lb} + (G^0 - E^0) + \left(G^0_{solv} - E^0\right) + const. \quad (1)$$

where E^0 and $E^{lb}$are electronic energies computed at the DFT level ωB97X-D/6-31G(d,p) + SDD (augmented by f polarization functions for the Ru centre) and ωB97X-D/6-311++G(2d,p) + SDD (augmented by *2f,1g* polarization functions for the Ru centre), respectively. $G^0$and G_{solv} refer to the gas-phase and solution phase Gibbs free energies calculated at ωB97X-D/6-31G(d,p) + SDD (augmented by f polarization functions for the Ru centre) level of theory. The coefficients of the additional polarization functions of Ru were taken from 16. The last term of eq. 1 is the correction (const. = 0.00302 a.u.) used for switching to the standard state of solutions (c = 1mol/dm^3 concentration). Ultrafine integration grid was used for all computations. The ωB97X-D was chosen as a good compromise of cost of the calculation and accuracy of optimized geometries for transition metal compounds.[S1]

S2. Comparison of experimental and calculated structures of precatalyst 1-A

The optimized structure of precatalyst [Ru(SINpEt)(SINpEt'')] **1-A** is compared to the experimentally identified crystal structure in Figure S1.

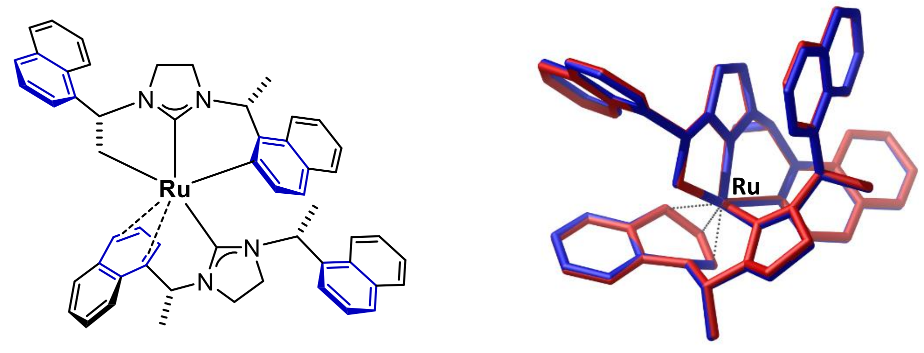

1-A

Figure S1: Overlay structure of calculated and XRD experimental structures of **1-A**. The hydrogen atoms are omitted for clarity.

S3. Energy data of the most stable conformers of 1-A, 1-B and 1-D

Table S1: Energy data computed for the optimized structures by using the range-separated ωB97X-D exchange-correlational functional.[1]

Struct	E^0	E^{lb}	G^0	$G^0_{toluene}$	$G^0_{n\text{-}hexane}$	$G_{toluene}$	$G_{n\text{-}hexane}$	ΔG_{tol}	$\Delta G_{n\text{-}hex}$
**1-A**	-2401.2014	-2401.7280	-2400.3557	-2401.2462	-2401.2432	-2400.9242	-2400.9213	**0.0**	**0.0**
**1-A_1**	-2401.1976	-2401.7246	-2400.3533	-2401.2435	-2401.2405	-2400.9233	-2400.9202	**0.6**	**0.7**
**1-A_2**	-2401.1879	-2401.7156	-2400.3446	-2401.2351	-2401.2321	-2400.9165	-2400.9134	**4.9**	**4.9**
**1-A_3**	-2401.1892	-2401.7176	-2400.3448	-2401.2352	-2401.2321	-2400.9160	-2400.9129	**5.1**	**5.2**
**1-A_4**	-2401.1834	-2401.7115	-2400.3399	-2401.2307	-2401.2276	-2400.9123	-2400.9091	**7.5**	**7.6**
**1-B**	-2402.4107	-2402.9371	-2401.5427	-2402.4558	-2402.4527	-2402.1111	-2402.1080	**-8.2**	**-8.0**
**1-B_1**	-2402.4085	-2402.9355	-2401.5417	-2402.4539	-2402.4507	-2402.1111	-2402.1080	**-8.2**	**-8.0**
**1-B_2**	-2402.4111	-2402.9374	-2401.5414	-2402.4566	-2402.4533	-2402.1101	-2402.1069	**-7.6**	**-7.3**
**1-B_3**	-2402.4087	-2402.9355	-2401.5406	-2402.4538	-2402.4506	-2402.1096	-2402.1064	**-7.2**	**-7.0**
**1-B_4**	-2402.4045	-2402.9316	-2401.5370	-2402.4501	-2402.4469	-2402.1067	-2402.1034	**-5.4**	**-5.1**
**1-D**	-2409.6948	-2410.2145	-2408.6942	-2409.7413	-2409.7379	-2409.2573	-2409.2540	**-72.7**	**-72.1**
**1-D_1**	-2409.6927	-2410.2126	-2408.6930	-2409.7378	-2409.7350	-2409.2551	-2409.2522	**-71.3**	**-71.0**
**1-D_2**	-2409.6882	-2410.2087	-2408.6893	-2409.7347	-2409.7316	-2409.2533	-2409.2502	**-70.2**	**-69.8**
**1-D_3**	-2409.6942	-2410.2136	-2408.6924	-2409.7380	-2409.7347	-2409.2526	-2409.2494	**-69.7**	**-69.2**
**1-D_4**	-2409.6918	-2410.2117	-2408.6900	-2409.7365	-2409.7333	-2409.2515	-2409.2483	**-69.1**	**-68.5**

[1]Notations are according to section **S1** and $G^0_{solvent}$ denotes the solution-phase Gibbs free energies for the *solvent=toluene* and *n-hexane*. All energies are expressed in a.u. The last two columns are the relative stabilities referred to the most stable structure **1-A** in kcal/mol.

S4. Catalyst form 1-F

In the computational process of identifying the active form of the catalyst we considered several possible configurations of the precatalyst which can be formed under H_2 pressure. One of the envisioned structures **1-F** resembling **1-D** exhibits three partially hydrogenated naphthyl ligands and two hydride ligands suitable for substrate hydrogenation. However, this catalyst form is energetically un-favoured and coordination to the metal centre in **1-F** is hampered by the η^4-type coordination to the ruthenium of the fourth naphthyl group.

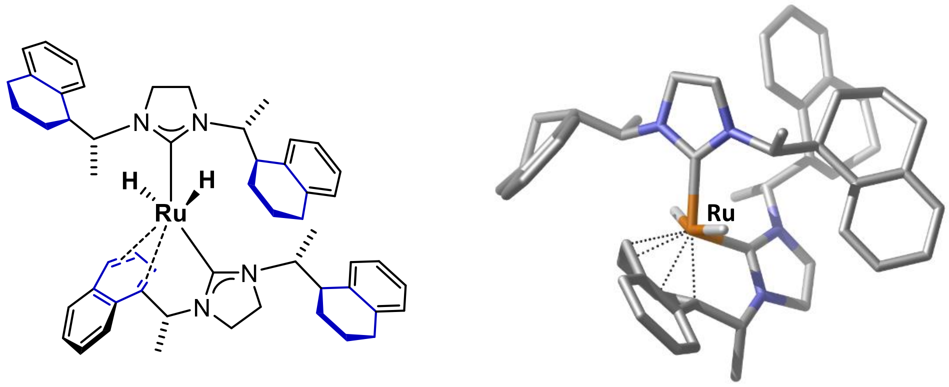

1-F **1-F** (20.0)

Figure S2: Optimized structure of **1-F** The number in parentheses is the relative Gibbs free energy in kcal/mol referred to **1-C$_{trans}$**.

S5. Computed catalyst isomers

The catalyst form suitable for asymmetric hydrogenation of heteroarenes must satisfy two main necessary requirements: free coordination site for the substrate and available hydride ligands for the two hydrogen transfers to the coordinated substrate. All feasible catalyst structures and their free energies relative to **1-C$_{trans}$** are presented in Figure S3. Each feasible catalyst complex has isomers *trans* and *cis,* referring to the arrangement of the carbene ligands.

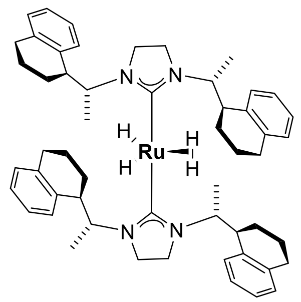

1-C

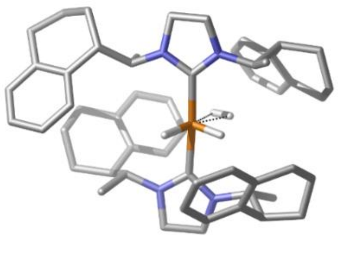

1-C$_{trans}$ (0.0)

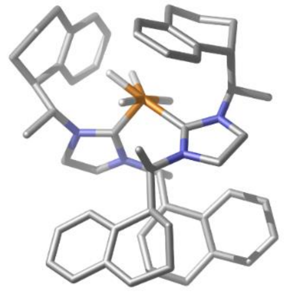

1-C$_{cis}$ (10.2)

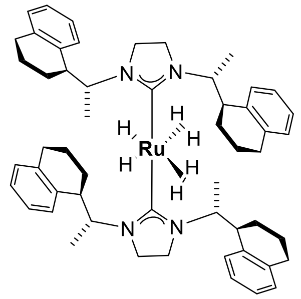

1-C′

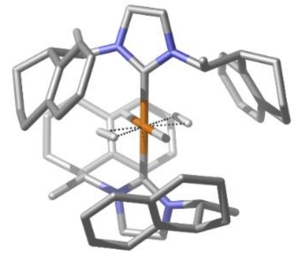

1-C′$_{trans}$ (-0.6)

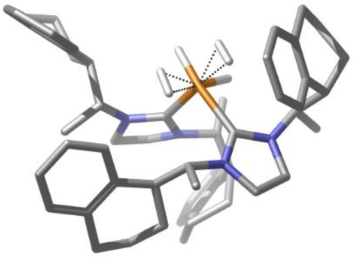

1-C′$_{cis}$ (3.4)

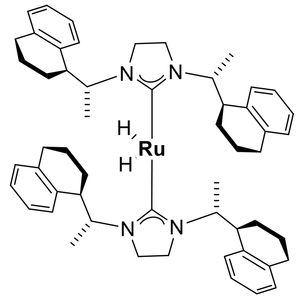

1-C″

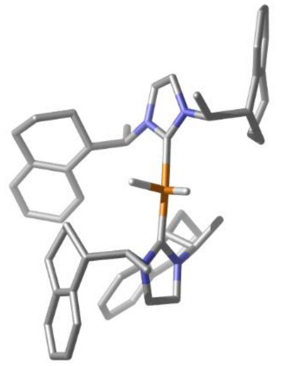

1-C″$_{trans}$ (7.2)

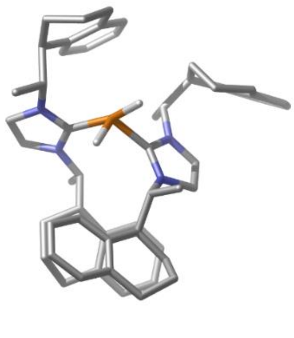

1-C″$_{cis}$ (8.0)

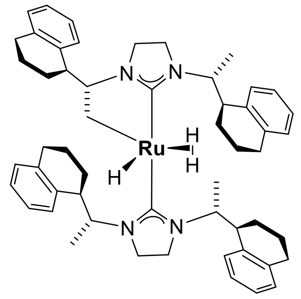

1-E

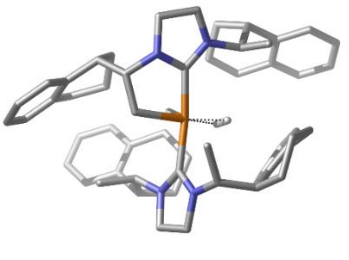

1-E$_{trans}$ (7.8)

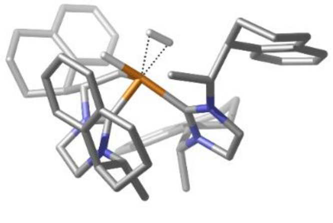

1-E$_{cis}$ (22.2)

Figure S3: Schematic structure of complexes **1-C, 1-C′, 1-C″** and **1-E.** Optimized geometries of *trans* and *cis* isomers for each complex are presented. The numbers in the parentheses are the relative Gibbs free energies referred to **1-C**$_{\mathbf{trans}}$ in kcal/mol. CH hydrogen atoms are omitted for clarity.

S6. Conformational space of 1-C_{trans}

The bulky naphthyl moieties in the NHC ligands are flexible and may adopt several arrangements. Most of the structures are stabilized by intramolecular attractive van der Waals interactions between the partially hydrogenated naphthyl groups of the ligands. The multitude of possible steric arrangements in the energy window of 0-9 kcal/mol is depicted in the overlay picture in Figure S4. The same complexity is characteristic to the conformational space of all intermediates and transition states along the reaction pathways studied.

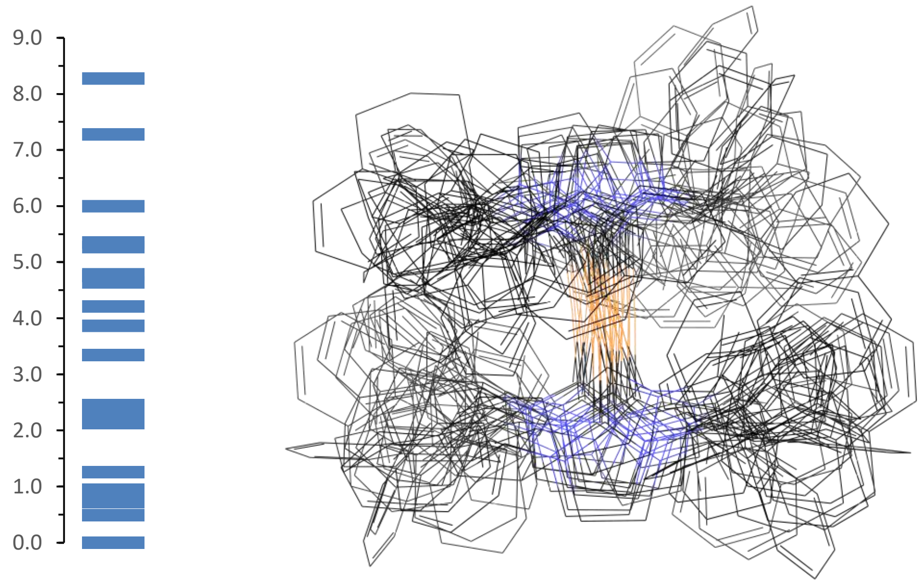

Figure S4: Superimposed structures of the optimized conformers in the free energy range of 9 kcal/mol. In the left the diagram of relative Gibbs free energies of these conformers related to **1-C_{trans}** is shown.

Table S2: Energy data computed for the optimized conformations of catalyst **1-C_{trans}** by using the range-separated ωB97X-D exchange-correlational functional.[2]

Struct	E^0	E^{lb}	G^0	$G^0_{toluene}$	$G^0_{n\text{-}hexane}$	$G_{toluene}$	$G_{n\text{-}hexane}$	ΔG_{tol}	$\Delta G_{n\text{-}hex}$
**1-C_{trans}**	-2414.4936	-2415.0104	-2413.4185	-2414.5454	-2414.5403	-2413.9840	-2413.9789	**0.0**	**0.0**
**1-C_{trans}_1**	-2414.4874	-2415.0055	-2413.4148	-2414.5408	-2414.5360	-2413.9832	-2413.9784	**0.5**	**0.3**
**1-C_{trans}_2**	-2414.5027	-2415.0195	-2413.4230	-2414.5488	-2414.5452	-2413.9828	-2413.9792	**0.7**	**-0.2**
**1-C_{trans}_3**	-2414.4931	-2415.0108	-2413.4191	-2414.5421	-2414.5380	-2413.9828	-2413.9787	**0.8**	**0.1**
**1-C_{trans}_4**	-2414.4947	-2415.0114	-2413.4183	-2414.5452	-2414.5404	-2413.9824	-2413.9776	**1.0**	**0.8**
**1-C_{trans}_5**	-2414.4901	-2415.0081	-2413.4165	-2414.5406	-2414.5364	-2413.9820	-2413.9778	**1.3**	**0.7**
**1-C_{trans}_6**	-2414.4946	-2415.0116	-2413.4179	-2414.5434	-2414.5393	-2413.9806	-2413.9764	**2.1**	**1.5**
**1-C_{trans}_7**	-2414.4945	-2415.0130	-2413.4181	-2414.5412	-2414.5378	-2413.9803	-2413.9769	**2.3**	**1.2**
**1-C_{trans}_8**	-2414.4965	-2415.0141	-2413.4186	-2414.5433	-2414.5395	-2413.9801	-2413.9763	**2.5**	**1.6**
**1-C_{trans}_9**	-2414.4986	-2415.0158	-2413.4185	-2414.5445	-2414.5410	-2413.9786	-2413.9751	**3.3**	**2.3**
**1-C_{trans}_10**	-2414.4905	-2415.0083	-2413.4144	-2414.5389	-2414.5349	-2413.9778	-2413.9737	**3.9**	**3.2**
**1-C_{trans}_11**	-2414.4977	-2415.0144	-2413.4164	-2414.5450	-2414.5409	-2413.9772	-2413.9731	**4.2**	**3.6**
**1-C_{trans}_12**	-2414.4890	-2415.0078	-2413.4118	-2414.5379	-2414.5341	-2413.9766	-2413.9728	**4.6**	**3.8**

**1-C$_{trans}$_13**	-2414.4894	-2415.0062	-2413.4127	-2414.5393	-2414.5351	-2413.9764	-2413.9721	**4.8**	**4.2**
**1-C$_{trans}$_14**	-2414.4894	-2415.0077	-2413.4131	-2414.5373	-2414.5336	-2413.9763	-2413.9727	**4.8**	**3.9**
**1-C$_{trans}$_15**	-2414.4833	-2415.0021	-2413.4087	-2414.5343	-2414.5301	-2413.9756	-2413.9714	**5.3**	**4.7**
**1-C$_{trans}$_16**	-2414.4939	-2415.0120	-2413.4151	-2414.5392	-2414.5358	-2413.9754	-2413.9721	**5.4**	**4.2**
**1-C$_{trans}$_17**	-2414.4871	-2415.0041	-2413.4106	-2414.5370	-2414.5329	-2413.9744	-2413.9703	**6.0**	**5.4**
**1-C$_{trans}$_18**	-2414.4845	-2415.0024	-2413.4083	-2414.5336	-2414.5293	-2413.9724	-2413.9681	**7.3**	**6.8**
**1-C$_{trans}$_19**	-2414.4897	-2415.0074	-2413.4120	-2414.5339	-2414.5309	-2413.9708	-2413.9678	**8.3**	**6.9**

[2]Notations correspond to the notation of Table S1. The Gibbs free energies are referred to **1-C$_{trans.}$**

S7. Free energy diagrams of catalyst-substrate complexes for the isomers of 1-C and cyclometalated 1-E

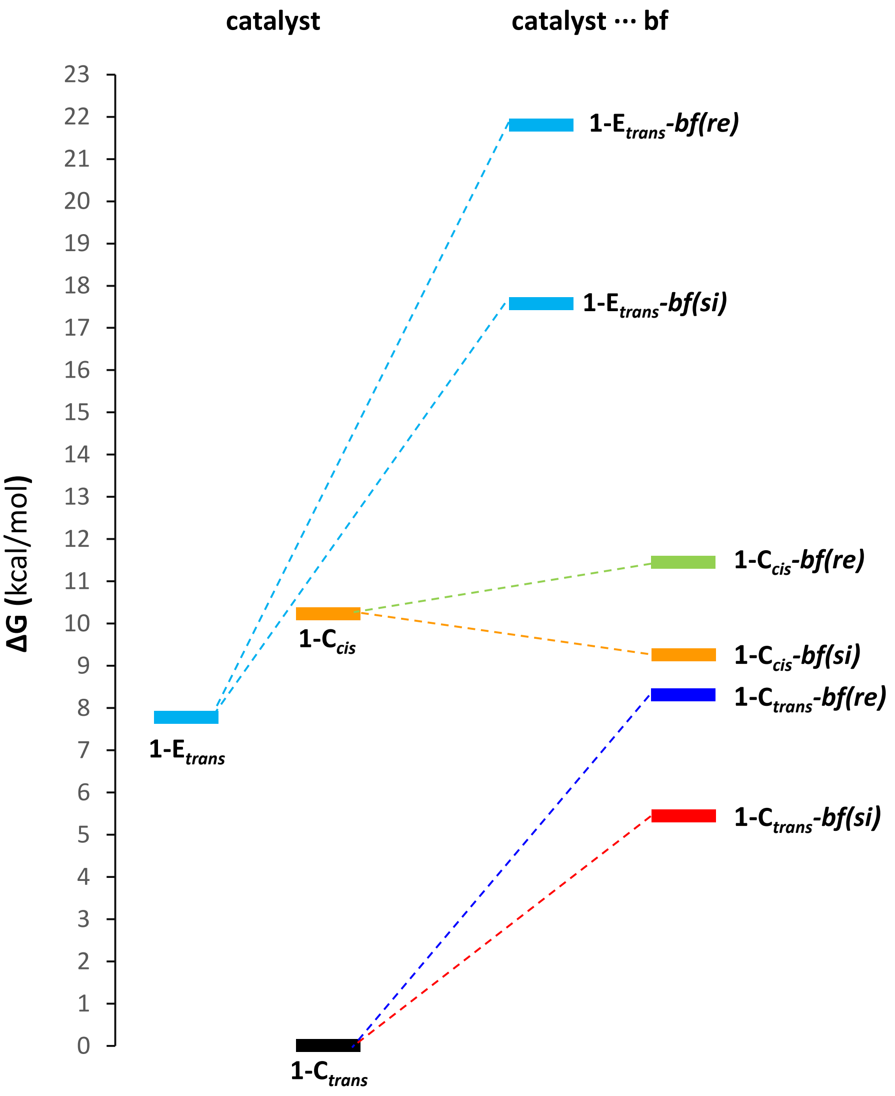

Figure S5: Free energy diagram for the catalyst isomers and the related catalyst…**bf** coordinated complexes.

Considering the arrangement of the carbene ligands in the complex the experimentally observed precatalyst structures **1-A**, **1-B** and **1-D** are *cis* isomers. In the light of this, we performed computational screening for the substrate coordination to all isomers of catalyst **1-C**, including *cis* isomers, too. The results show that even though the *cis* isomer of the catalyst is less stable than **1-C$_{trans}$** by 10 kcal/mol, the coordinated complex **1-C$_{cis}$-bf(si)** is of comparable energy to **1-C$_{trans}$-bf(si)**. Structure **1-E$_{trans}$** is more stable than **1-C$_{cis}$** but the coordination of the substrate to catalyst **1-E$_{trans}$** is computed to be high in free energy. The stabilities of different catalyst forms and related coordination complexes are depicted in the energy diagram of Figure S5.

S8. Coordination complexes

S8.1 Structures of coordination complexes 1-E$_{trans}$-bf(si) and 1-E$_{trans}$-bf(re)

We assume that the hydrogenation of **bf** is feasible by the catalyst form **1-E$_{trans}$**, as it exhibits three hydride ligands and a free coordination site. The extensive conformational search of the coordinated complexes for the *si*- and *re*-face approach of the substrate resulted in the lowest lying structures **1-E$_{trans}$-bf(si)** and **1-E$_{trans}$-bf(re)** presented in Figure S6. The atoms 2C and 3C of **bf** are activated similarly to the corresponding **1-C$_{trans}$-bf(si)** and **1-C$_{trans}$-bf(re)** complexes. However, the relative Gibbs free energies (17.6 and 21.8 kcal/mol) of these complexes relative to the free **1-C$_{trans}$** + **bf** reactant state lie too high in energy, leading to unfeasible reaction pathways.

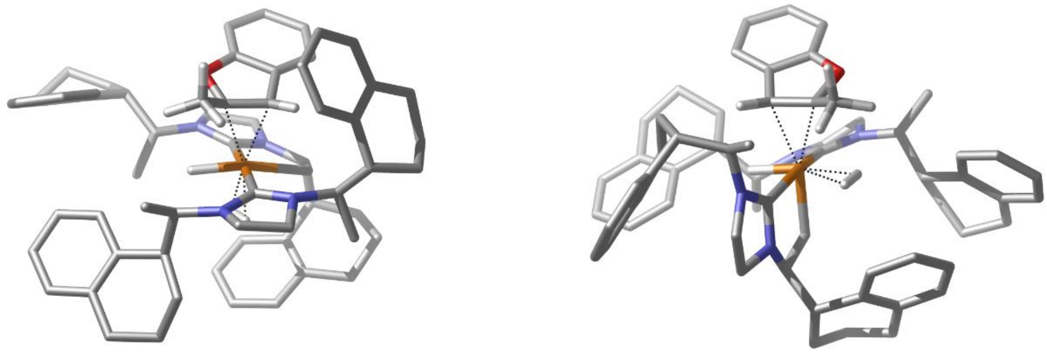

1-E$_{trans}$-bf(si) (17.6) **1-E$_{trans}$-bf(re)** (21.8)

Figure S6: The energetically lowest lying conformers of substrate complexes **1-E$_{trans}$-bf(si)** and **1-E$_{trans}$-bf(re)**. Relative Gibbs-free energies in parentheses are in kcal/mol.

Table S3: Energy data computed for the optimized structures by using the range-separated ωB97X-D exchange-correlational functional.[3]

Struct	E^0	E^{lb}	G^0	$G^0_{toluene}$	$G^0_{n\text{-}hexane}$	$G_{toluene}$	$G_{n\text{-}hexane}$	ΔG_{tol}	$\Delta G_{n\text{-}hex}$
1-E$_{trans}$	-2413.3030	-2413.8204	-2412.2399	-2413.3465	-2413.3433	-2412.7977	-2412.7945	**7.8**	**6.5**
1-E$_{trans}$-bf(si)	-2836.1815	-2836.8020	-2834.9782	-2836.2289	-2836.2259	-2835.6432	-2835.6402	**17.6**	**15.8**
1-E$_{trans}$-bf(re)	-2836.1761	-2836.7964	-2834.9712	-2836.2241	-2836.2211	-2835.6365	-2835.6335	**21.8**	**20.0**

[3]Notations correspond to the notation of Table S1. The Gibbs free energy of **1-E$_{trans}$** is relative to **1-C$_{trans.}$** The reference for complexes **1-E$_{trans}$-bf(si)** and **1-E$_{trans}$-bf(re)** is non-interacting **1-C$_{trans}$** and substrate **bf**.

S8.2 Other forms of coordination complexes 1-C$_{trans}$-bf

We explored several other forms of interaction of substrate **bf** with catalyst **1-C$_{trans}$.** The approach of substrate **bf** by its benzene part to the Ru centre of the catalyst is presented in Figure S7. A stable complex could be localized with a relatively low free energy in which the **bf** is bonded by non-covalent forces to the catalyst. The coordination to the metal centre of the benzene moiety with activated H atoms lies very high in free energy (at 24.9 kcal/mol).

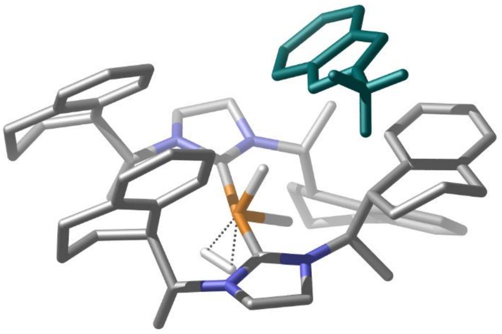

1-C$_{trans}$-bf(bz) (8.8)

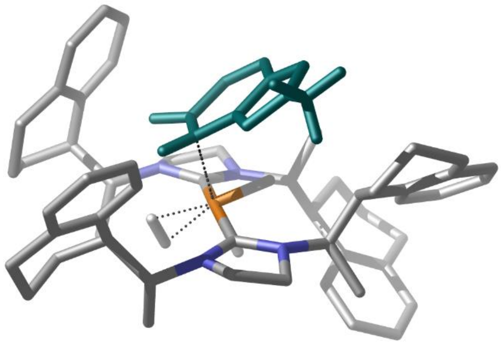

1-C$_{trans}$-bf(bz-activated) (24.9)

Figure S7: Computed structures of substrate complexes **1-C$_{trans}$-bf(bz)** and **1-C$_{trans}$-bf(bz-activated)**. Relative Gibbs free energies relative to the non-interacting **1-C$_{trans}$** and **bf** are shown in parentheses (in kcal/mol).

In conclusion, while the localization of the **bf** via the benzene core in close proximity to the complex is feasible by stabilizing non-covalent interactions, the activation of the benzene core by the Ru centre is very unlikely.

S8.3 Solvent coordination to the catalyst

The computed structure for the interacting solvent molecule with catalyst **1-C$_{trans}$** is depicted in Figure S8. The stability of this complex is comparable with that of complex **1-C$_{trans}$-bf(si)**, however the solvent molecule does not interacts with the ruthenium centre and the H atoms are not activated for further hydrogen transfer.

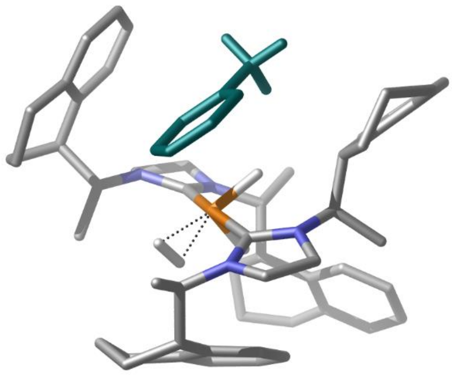

1-C$_{trans}$-toluene (8.3)

Figure S8: Computed structures of solvent-catalyst complex **1-C$_{trans}$-toluene**. Relative Gibbs free energies relative to the non-interacting **1-C$_{trans}$** and **toluene** are shown in parentheses (in kcal/mol).

In conclusion the presented substrate complexes and solvent-catalyst complex do not represent resting states of the reaction pathways as well as they do not open up feasible reaction pathways.

S9. Reaction pathways for catalyst form 1-C″

Catalyst form **1-C″** is less stable than **1-C$_{trans}$** by 7-8 kcal/mol (see Figure S3). In order to assess the feasibility of reaction pathways based on catalyst **1-C″** we explored the coordination of **bf** to **1-C″$_{trans}$** and **1-C″$_{cis}$**. The estimated free energy of the most stable complex **1-C″$_{cis}$-bf(si)** is 9.5 kcal/mol with respect to the **1-C$_{trans}$** + **bf** + H_2 reference state (see Figure S9). All other complexes are above 14.6 kcal/mol (see the free energy values in Table S4). The most stable localized **HT** transition state was found at 16.5 kcal/mol which is significantly higher than the respective barrier of pathway *trans(si).* These large free energy values of structures involving **1-C″**-type catalysts can be related to the reduced number of ligands on the Ru centre.

Based on these computations we can conclude that reaction pathways for the catalyst configuration **1-C″** are not feasible.

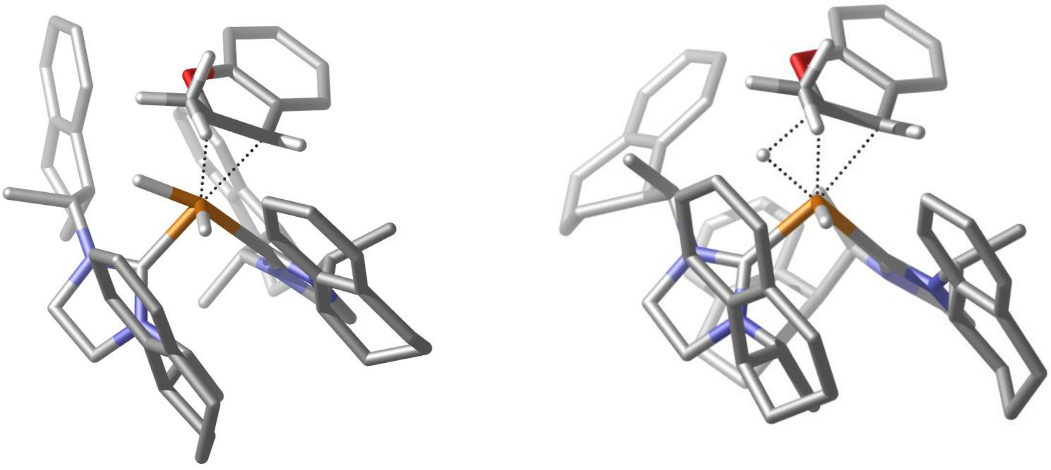

1-C″$_{cis}$-bf(si) (9.5) **TS^{HT}_{cis}(si)** (16.5)

Figure S9: Optimized, energetically lowest lying structures of coordinated complex **1-C″$_{cis}$-bf(si)** and transition state **TS^{HT}_{cis}(si)**. Relative Gibbs free energies in parentheses are in kcal/mol with respect to the **1-C$_{trans}$** + **bf** + H_2 reference state.

Table S4: Energy data computed for transition states corresponding to the transformation *cis/trans* by using the range-separated ωB97X-D exchange-correlational functional.[4]

Struct	*E^0*	*E^{lb}*	*G^0*	*$G^0_{toluene}$*	*$G^0_{n\text{-}hexane}$*	*$G_{toluene}$*	*$G_{n\text{-}hexane}$*	*ΔG_{tol}*	*$\Delta G_{n\text{-}hex}$*
**1-C″$_{trans}$-bf(si)**	-2836.1853	-2836.8042	-2834.9811	-2836.2362	-2836.2324	-2835.6480	-2835.6442	**14.6**	**13.3**
**1-C″$_{trans}$-bf(re)**	-2836.1619	-2836.7821	-2834.9591	-2836.2149	-2836.2102	-2835.6294	-2835.6247	**26.3**	**29.1**
**1-C″$_{cis}$-bf(si)**	-2836.1963	-2836.8147	-2834.9903	-2836.2467	-2836.2425	-2835.6561	-2835.6519	**9.5**	**12.1**
**1-C″$_{cis}$-bf(re)**	-2836.1827	-2836.8013	-2834.9748	-2836.2300	-2836.2264	-2835.6378	-2835.6342	**21.0**	**23.2**
**TS^{HT}_{cis}(si)**	-2836.1825	-2836.8022	-2834.9793	-2836.2316	-2836.2278	-2835.6450	-2835.6412	**16.5**	**18.8**

[4]Notations correspond to the notation of Table S1. The reference state is **1-C$_{trans}$** + **bf** + H_2.

S10. Comparison of pathways *cis/trans*

In order to obtain a detailed understanding of the asymmetric hydrogenation reaction of **bf** we computed and analysed the reaction pathways corresponding to both *cis* and *trans*-isomers (referring to the position of carbene ligands) of the active catalyst. The optimized structures of the coordinated complexes show that the substrate binding to the ruthenium centre occurs in η^2–manner for both the *si*- or *re*-face coordination of the C=C double bond of **bf**. The position of hydrogens suitable for hydride- and proton transfer in the coordinated complexes are highlighted in Figure S10.

The first elementary step of the reaction involves the transfer of a hydride ligand from the ruthenium catalyst to carbon centre 2 (C2) of the coordinated substrate. Note that the hydride ligands for both coordinated complexes are pre-organized for an easy hydride transfer to C2.

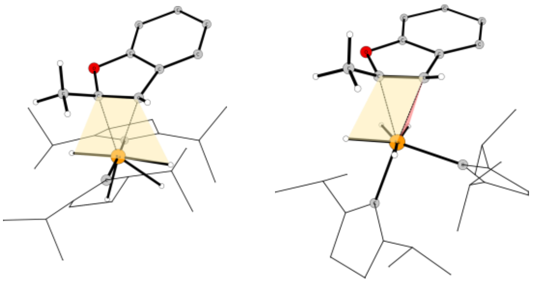

1-C$_{trans}$-bf(si) (5.4) **1-C$_{cis}$-bf(si)** (9.3)

Figure S10. Optimized structures of coordination complexes **1-C$_{trans}$-bf(si)** and **1-C$_{cis}$-bf(si)**. The arrangements of the pre-organized hydride ligands are highlighted in yellow, the red shape shows the less accessible hydride ligand. The naphthyl-groups of the catalyst have been omitted for clarity. Relative Gibbs free energies in parentheses are in kcal/mol with respect to the **1-C$_{trans}$** + **bf** + H_2 reference state.

The substrate in the resulting intermediates **int$^1_{trans}$(si)** and **int$^1_{cis}$(si)** is bound to the ruthenium centre in η^2–manner via an agostic interaction with the newly formed C-H bond. In spite of several analogies of the two complexes, the *cis* intermediate lies considerably higher in free energy. The following reaction step, the proton transfer, is also very favourable for catalyst **1-C$_{trans}$** due to the easy availability of the hydride ligand. However, the computations predict the PT transition state for the *cis* counterpart at 21.9 kcal/mol. The high barrier of the proton transfer **TS$^{PT}_{cis}$(si)** is the consequence of the specific geometry of the cis catalyst isomer. The hydride ligand required for the proton transfer in complex **int$^1_{cis}$(si)** lies almost perpendicular to the C-C double bond of **bf**. A complex geometry rearrangement takes place during the proton transfer and the result is the elevated energy of this step of the reaction.

The *re*-face coordination of the substrate to the catalyst provides reaction pathways towards the (*S*)-product isomer. The proton transfer step **TS$^{PT}_{cis}$(re)** has similar structural characteristics to **TS$^{PT}_{cis}$(si)** discussed above, and is energetically similarly destabilized. The *cis(si)* and *cis(re)* pathways are compared to *trans(si)* in the free energy profile in Figure S12. These results confirm the unfeasibility of reaction pathways *cis(si)* and *cis(re)* as compared to *trans(si)*.

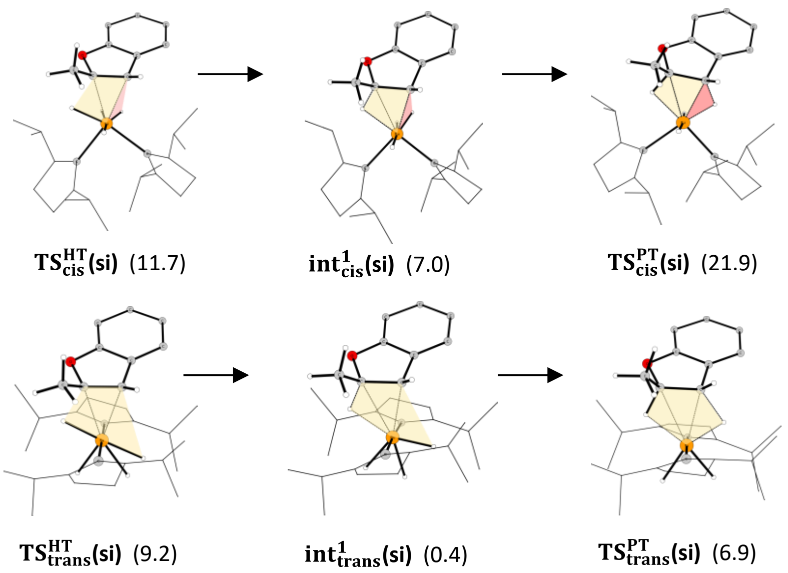

Figure S11: Optimized structures of stationary points on the reaction pathway corresponding to catalyst **1-C$_{trans}$** and **1-C$_{cis}$** towards the product enantiomer *R*. The arrangements of pre-organized hydride ligands are highlighted in yellow, the red shapes show less accessible hydride ligands. Relative Gibbs free energies in parentheses are in kcal/mol with respect to the **1-C$_{trans}$** + **bf** + H_2 reference state.

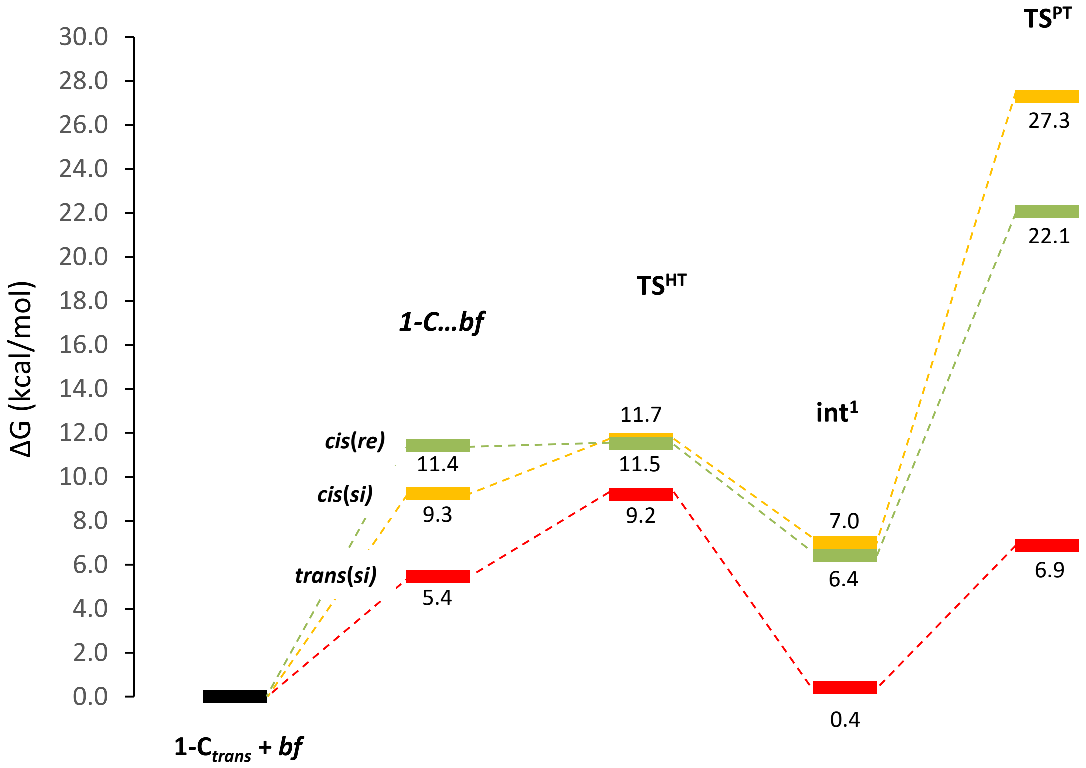

Figure S12: Diagram for pathways *trans(si)*, *cis(si)* and *cis(re)*. The numbers are relative Gibbs free energies in kcal/mol with respect to the **1-C$_{trans}$** + **bf** + H_2 reference state.

To examine the role of the bulky NHC ligands in the *cis* and *trans* reaction pathways, a truncated model system has been considered. In this model the naphthyl groups were substituted by H atoms and transition states **HT** and **PT** were re-optimized for the *cis*- and *trans*- isomers of the catalyst, respectively. The optimized geometries of the model systems are similar to the original structures. The

results for the model system show that the **HT** step of the reaction is of comparable energy or even more favourable for the *cis*- and *trans*-isomer of the catalyst. At the same time, the **PT** transition state lies high in energy for the *cis* isomer, suggesting that this step of the reaction is unfavourable due to the *cis* arrangement of carbene ligands of the catalyst with and without naphthyl substituents.

Table S5: Energy data computed for the computed transition states of the truncated model system.[5]

model	TS^{HT}	TS^{PT}
trans(si)	0.0	-0.2
trans(re)	-0.4	-1.7
cis(si)	-4.6	8.9
cis(re)	-0.9	5.1

[5]The numbers are relative Gibbs free energies in kcal/mol relative to **model-TS$^{HT}_{trans}$(si)**.

S11. Transition states corresponding to the *cis/trans*-transformation of catalyst 1-C and coordinated complex 1-C-bf(si)

On the reaction pathway towards the minor product isomer, the **TS$^{HT}_{cis}$(re)** transition state is lower in energy by 2.2 kcal/mol compared to the *trans* counterpart **TS$^{HT}_{trans}$(re)**. The calculations predicted that the pure *cis*- pathways is unfeasible. However, a suitable crossing between the *cis* and *trans*-pathways could lead to an alternative mixed *cis/trans* pathway towards the minor product.

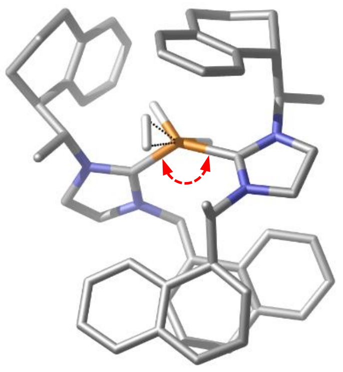

TS(cis/trans)$^{1\text{-}C}$ (11.9)

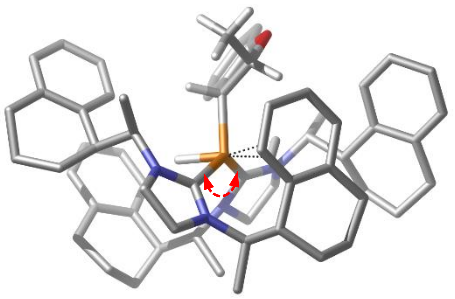

TS(cis/trans)$^{int1(re)}$ (20.4)

Figure S13: TS structures for the *cis/trans* transformation of catalyst **1-C** and for the intermediate following the HT step of the reaction (**int^1(re)**). The Gibbs-free energies are in kcal/mol, relative to the optimized catalyst **1-C$_{trans}$** for **TS(cis/trans)$^{1\text{-}C}$** and relative to the **1-C$_{trans}$ + bf** reactant state for **TS(cis/trans)$^{int1(re)}$**, respectively.

For this purpose, we examined the *cis/trans* transformation of catalyst **1-C** as well as the crossing of the two pathways after the **HT** step in detail. The structures are depicted in Figure S13. The barrier connecting isomers **1-C$_{cis}$** (10.2 kcal/mol) and **1-C$_{trans}$** (0.0 kcal/mol) is at 11.9 kcal/mol, which is comparable to the transition state of the hydrogen transfer steps of the reaction. However, the equilibrium is strongly shifted toward the *trans*-isomer of catalyst **1-C**.

Table S6: Energy data computed for transition states corresponding to the *cis/trans* transformation by using the range-separated ωB97X-D exchange-correlational functional.[6]

Struct	E^0	E^{lb}	G^0	$G^0_{toluene}$	$G^0_{n\text{-}hexane}$	$G_{toluene}$	$G_{n\text{-}hexane}$	ΔG_{tol}	$\Delta G_{n\text{-}hex}$
**TS(cis/tr)$^{1\text{-}C}$**	-2414.4814	-2414.9989	-2413.4047	-2414.5272	-2414.5234	-2413.9650	-2413.9611	**11.9**	**14.3**
**TS(cis/tr)$^{bf(re)}$**	-2837.3703	-2837.9899	-2836.1453	-2837.4212	-2837.4172	-2836.8127	-2836.8087	**20.4**	**19.2**

[6]Notations correspond to the notation of Table S1.

The calculated free energy of 20.4 kcal/mol corresponding to the *cis/trans* transformation of the catalyst in complex **int^1(re)** lies too high compared to all of the hydrogen transfer steps for the *trans* pathway. These results corroborate the hypothesis that the form of the active catalyst is **1-C$_{trans}$** and the reaction proceeds along the pathways *trans(re)* and *trans(si)*.

S12. Reaction pathway for the *trans*-dihydride catalyst 1-C$_{trans}$-H$_2$*trans*

The starting structure for the most favourable reaction pathway is *cis*-dihydride substrate complex **1-C$_{trans}$-bf(si).** Here, *cis*-dihydride refers to the *cis* arrangement of the two hydride ligands, while the *trans* subscript denotes the relative position of the two carbene ligands. The computed *trans*-dihydride counterpart **1-C$_{trans}$–H$_2$*trans*-bf(si)** lies 2.3 kcal/mol above **1-C$_{trans}$-bf(si)** in free energy (see Figure S14). The conformational search for the coordinated complex **1-C$_{trans}$–H$_2$*trans*-bf(si)** revealed that all ten structures optimized at the DFT level of theory were higher in energy (in the range of 10.4 – 18.0 kcal/mol relative to separated **1-C$_{trans}$** and **bf**). The following H$_2$*trans* HT transition state structure and intermediate are very close in energy to **TS^{HT}(si)** and **int^1(si)** (*cis*-dihydride pathway), respectively. Following the reaction pathway by the Intrinsic Reaction Coordinate method we find that in the structure of the PT transition state the dihydrogen ligand is split and the located structure is identical to **TS^{PT}(si)** (see Figure S15).

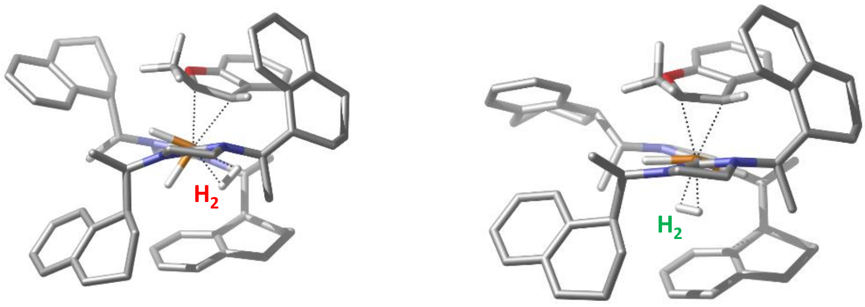

1-C$_{trans}$-bf(si) (5.4) **1-C$_{trans}$-H$_2$trans-bf(si) (7.7)**

Figure S14: Optimized structures of *cis*- and *trans*-dihydride substrate complexes. The numbers in the parentheses are relative Gibbs free energies in kcal/mol relative to non-interacting **1-C$_{trans}$** and **bf**. All H atoms of the NHC ligands are omitted for clarity.

The transition state corresponding to *cis*-dihydride/*trans*-dihydride isomerization for the coordinated complex lies at 11.2 kcal/mol in free energy, which is higher than the HT and PT transition states of the *trans(si)* pathway. Accordingly, complex **1-C$_{trans}$–H$_2$*trans*-bf(si)** will form with a certain probability, but the reaction will predominantly follow pathway *trans(si)*. The free energy diagrams for the *trans(si)* and *H$_2$trans-trans(si)* pathways are compared in Figure S16.

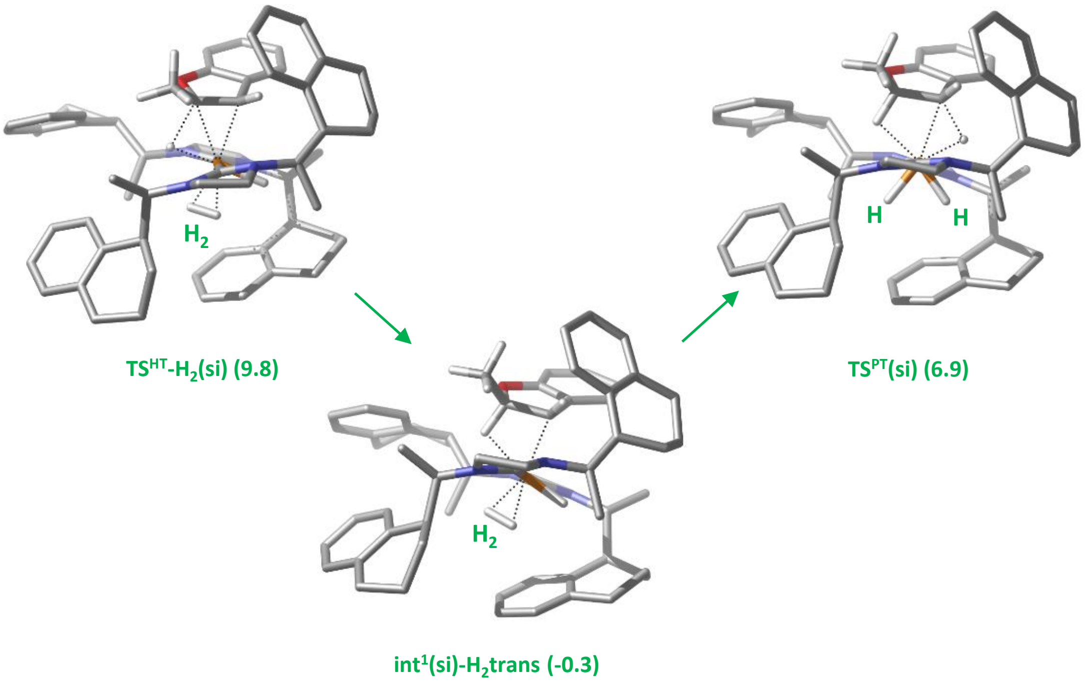

Figure S15: Optimized structures of elementary steps for trans-H_2trans(si) pathway. The numbers in the parentheses are relative Gibbs free energies in kcal/mol relative to non-interacting **1-C$_{trans}$** and **bf**. All H atoms of the NHC ligands are omitted for clarity.

The transition state corresponding to *cis*-dihydride/*trans*-dihydride isomerization for the coordinated complex lies at 11.2 kcal/mol in free energy, which is higher than the HT and PT transition states of the *trans(si)* pathway. Accordingly, complex **1-C$_{trans}$–H_2*trans*-bf(si)** will form with a certain probability, but the reaction will predominantly follow pathway *trans(si)*. The free energy diagrams for the *trans(si)* and *H_2trans-trans(si)* pathways are compared in Figure S16.

In an attempt to locate the trans-dihydride isomer of the substrate complex leading to the minor reaction product, the structure **1-C$_{trans}$–H_2trans-bf(re)** shown in Figure S17 was found which lies at 16 kcal/mol. The transition state corresponding to the *cis*-dihydride/*trans*-dihydride transformation of the minor substrate complex could not be located.

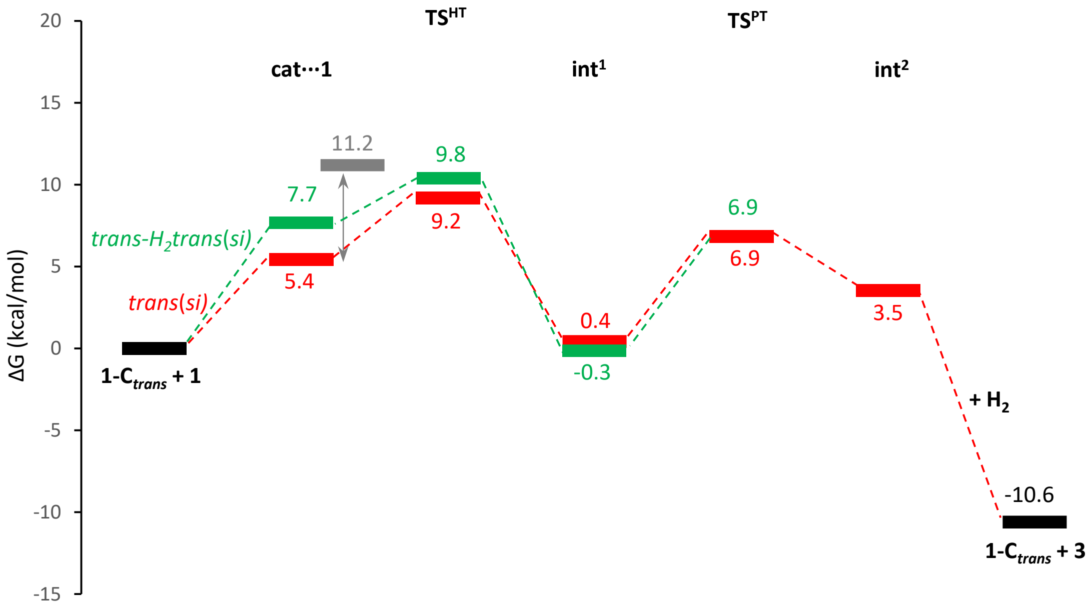

Figure S16: Free energy diagrams for pathways *trans(si)* and *H_2trans-trans(si)* the numbers are relative Gibbs free energies in kcal/mol. The grey bar corresponds to the transition state of cis-dihydride/trans-dihydride isomerization.

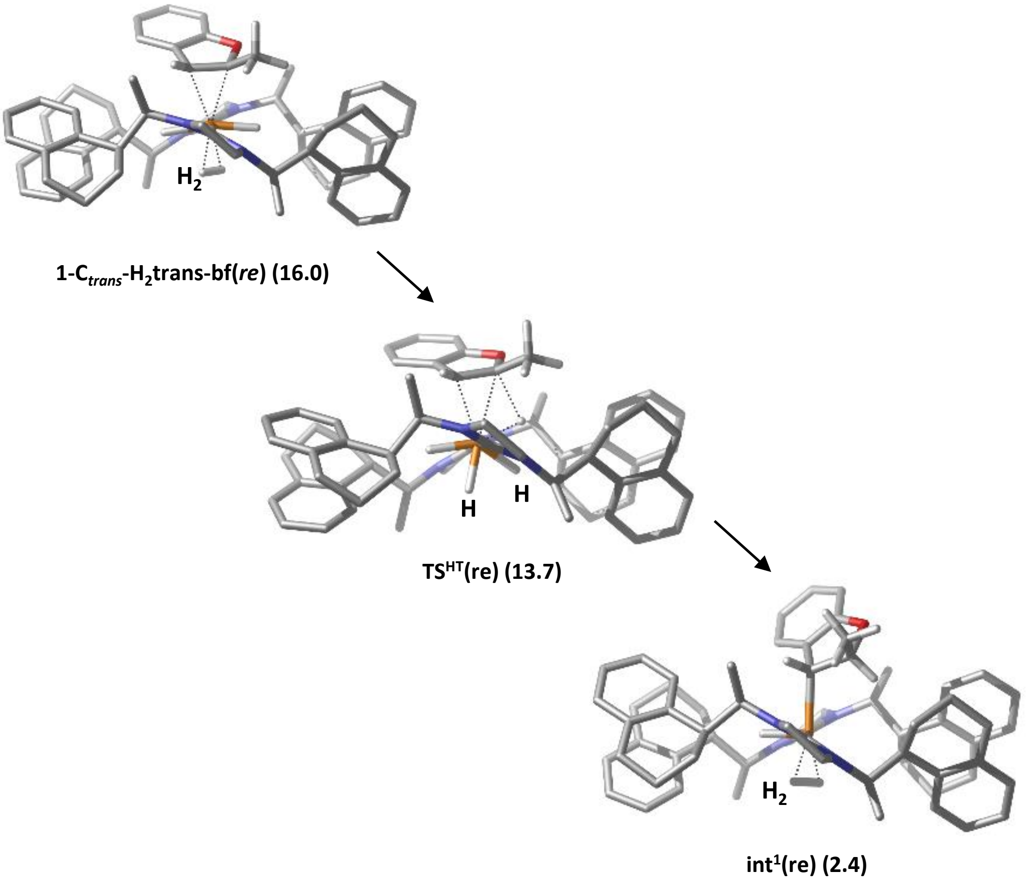

Figure S17: Optimized structures for the trans-dihydride substrate complex and the following **HT** transition state and first intermediate on the route to the minor product. The numbers in parentheses are relative Gibbs free energies in kcal/mol relative to non-interacting **1-C$_{trans}$** and **bf**. All H atoms of the NHC ligands are omitted for clarity.

Furthermore, a scan calculation starting from **1-C$_{trans}$–H$_2$trans-bf(re)** along the IRC to locate the transition state of the hydride transfer led to the original **TS^{HT}(re)** (see Figure S17). This indicates that the transformation between complexes **1-C$_{trans}$–H$_2$trans-bf(re)** and **1-C$_{trans}$-bf(re)** proceeds most probably barrierless or via a very small barrier. Five computed structures of the conformational search for complex **1-C$_{trans}$–H$_2$trans-bf(re)** were above 18.7 kcal/mol in free energy. As shown in Figure S17 the structure of the first intermediate **int^1(re)** is a *cis*-dihydride isomer. This suggests that the splitting and bond forming of the dihydrogen ligand happens in a dynamic manner along the reaction pathway.

In conclusion, the *cis*-dihydride reaction pathways *trans(si)* and *trans(re)* are energetically favourable compared to the studied *trans*-dihydride pathways.

S13. Stability of "pocket" and "flat" conformers of 1-C$_{trans}$

With the aim of elucidating the origin of stereoselectivity we conducted calculations for the different conformations of catalyst **1-C$_{trans}$**, taken from the coordinated complexes **1-C$_{trans}$-bf(si)**, and **1-C$_{trans}$-bf(re)**. For this purpose, we re-optimized the catalyst structures based on the geometry of the coordinated complexes but without the substrate **bf**. As expected, the optimized catalyst **1-C$_{trans}$(si)** of the major pathway preserved the closed, "pocket"-type form, which is slightly less stable than **1-C$_{trans}$** (by 2.4 kcal/mol). At the same time the "flat" configuration of the catalyst corresponding to **1-C$_{trans}$-bf(re)** remained unchanged, but lying 7.6 kcal/mol above **1-C$_{trans}$** (see Figure S18).

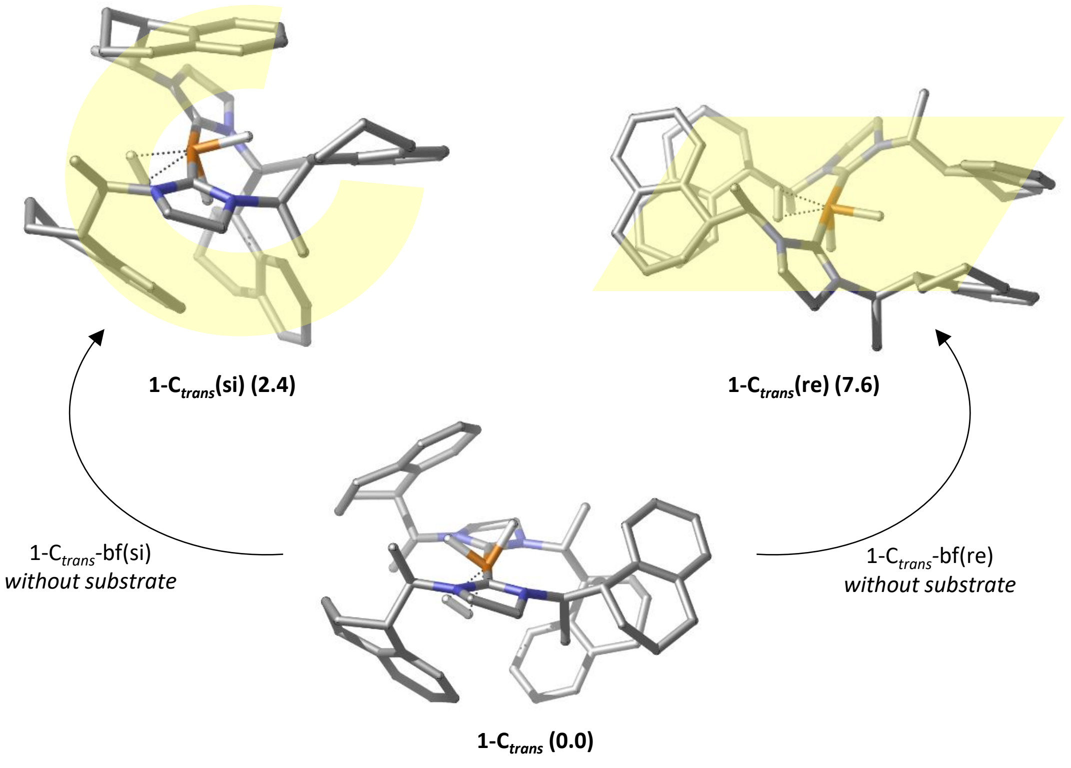

Figure S18: Optimized structures of catalyst **1-C$_{trans}$** and re-optimized from the coordinated complexes **1-Ctrans-bf(si)** and **1-Ctrans-bf(re)**. The numbers in parentheses are Gibbs free energies relative to **1-C$_{trans}$** in kcal/mol.

The difference in energy between the "pocket" and "flat" configurations of the catalyst suggests that the stereoselectivity of the reaction is already induced at the level of the catalyst. The stability of the "pocket" catalyst foreshadows the preference for this form along the reaction pathway towards the

major product isomer. At the same time the chiral pocket formed by the aromatic groups of the ligands acts as a binding site for the approaching substrate.

S14. “Pocket” configuration for 1-C_{trans}-bf(re)

In order to assess the exclusive presence of the “pocket” catalyst along the major pathway, corresponding to the *si*-face approach, we probed the possibility of *re*-face coordination of **bf** into the chiral pocket. For this purpose, we first computed the *re*-analogue of the “pocket” complex **1-C_{trans}-bf(si)**. The optimization lead to a stable form lying at 16.2 kcal/mol with relative to the **1-C_{trans}** + **bf** reference state. The high endergonicity comes from the steric hindrance between the methyl group of the substrate and the naphthyl-group of the catalyst forming the pocket. The repulsive interaction is shown by the blue dotted arrow in Figure S19. The overlay structure of the computed structures (see Figure S17, bottom) indicates that indeed the ligand groups of the pocket opened up to allow the *re*-face coordination.

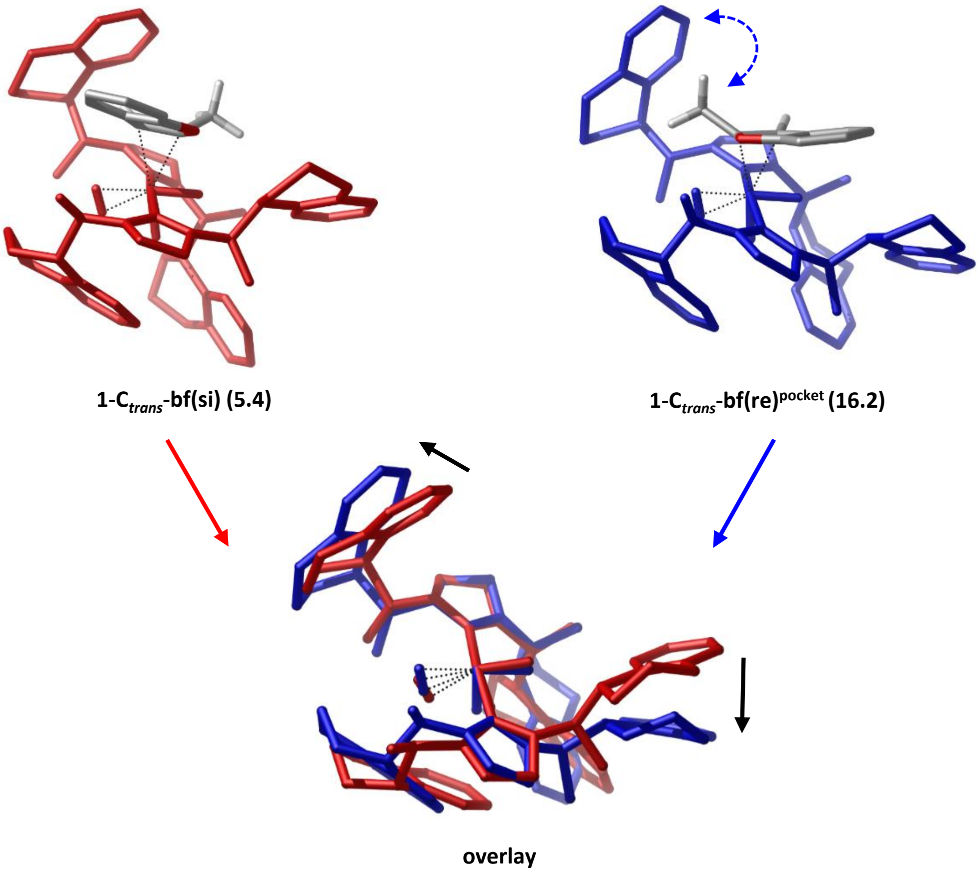

Figure S19: Optimized structures of coordinated complexes **1-C_{trans}-bf(si)** and **1-C_{trans}-bf(re)^{pocket}** (top). Overlay structure of **1-C_{trans}-bf(si)** and **1-C_{trans}-bf(re)^{pocket}** (bottom). The changes in the geometry of the catalyst are illustrated by black arrows. The substrate and the hydrogen atoms of the ligand moieties are omitted for clarity. Relative Gibbs free energies (in kcal/mol) relative to **1-C_{trans}** and **bf** are given in parentheses.

With the aim of by-passing the steric repulsion between the ligand and the methyl group of the substrate in the pocket, we optimized a modified **1-C_{trans}-bf(re)^{pocket}** complex, in which the **bf** in the pocket is rotated by 180° around an axis perpendicular to the plane of **bf**. However, the computed free energy is predicted to be extremely high (27.3 kcal/mol) with respect to the **1-C_{trans}** and **bf** reference.

Closer inspection of the structure revealed several short H-H distances between the hydrogens of **bf** and the catalyst indicating repulsive interactions (shown in blue lines in Figure S20). The chiral pocket is also highly distorted and the interaction with the coordinating **bf** is un-favourable.

This analysis confirms that substrate coordination into the chiral pocket is privileged only for *si*-face attack, which leads to the major product isomer.

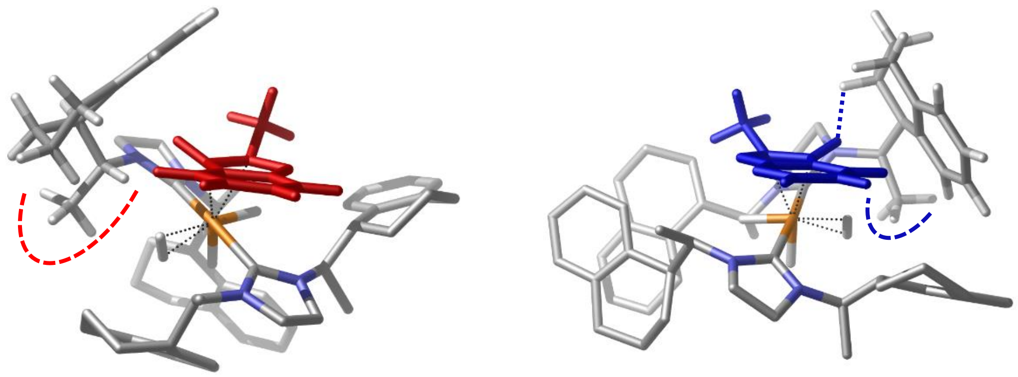

1-C$_{trans}$-bf(si) (5.4) **1-C$_{trans}$-bf$_{rot}$ (re)^{pocket} (27.3)**

Figure S20: Optimized structures of coordinated complexes **1-C$_{trans}$-bf(si)** and **1-C$_{trans}$-bf$_{rot}$(re)^{pocket}**. The blue dotted lines indicate the short H…H distance and the sterically hindered methyl group of the catalyst, while the red dotted line is the analogous methyl group of the latter. Relative Gibbs free energies (in kcal/mol) relative to **1-C$_{trans}$** and **bf** reactant state are given in parentheses.

Other possible conformations of **1-C$_{trans}$-bf(re)^{pocket}** were explored computationally. The results indicate that all such located conformers are in the free energy range of 24.8–33.1 kcal/mol relative to **1-C$_{trans}$** and **bf** reactant state. The energies are collected in Table S7.

Table S7: Energy data computed for the optimized conformations of **1-C$_{trans}$-bf(re)^{pocket}** (denoted conf_nr) by using the range-separated ωB97X-D exchange-correlational functional.[7]

Struct	E^0	E^{lb}	G^0	$G^0_{toluene}$	$G^0_{n\text{-}hexane}$	$G_{toluene}$	$G_{n\text{-}hexane}$	ΔG_{tol}	$\Delta G_{n\text{-}hex}$
**conf_1**	-2837.3762	-2837.9959	-2836.1536	-2837.4252	-2837.4215	-2836.8193	-2836.8157	**16.2**	**14.9**
**conf_2**	-2837.3572	-2837.9788	-2836.1372	-2837.4050	-2837.4018	-2836.8036	-2836.8003	**26.1**	**24.5**
**conf_3**	-2837.3585	-2837.9795	-2836.1370	-2837.4054	-2837.4021	-2836.8019	-2836.7986	**27.1**	**25.6**
**conf_4**	-2837.3544	-2837.9757	-2836.1348	-2837.4029	-2837.3997	-2836.8016	-2836.7985	**27.3**	**25.6**
**conf_5**	-2837.3555	-2837.9770	-2836.1329	-2837.4038	-2837.4006	-2836.7998	-2836.7965	**28.5**	**26.9**
**conf_6**	-2837.3545	-2837.9760	-2836.1321	-2837.4026	-2837.3994	-2836.7986	-2836.7954	**29.2**	**27.6**
**conf_7**	-2837.3520	-2837.9742	-2836.1306	-2837.3999	-2837.3968	-2836.7977	-2836.7946	**29.8**	**28.1**
**conf_8**	-2837.3568	-2837.9779	-2836.1328	-2837.4032	-2837.4000	-2836.7973	-2836.7941	**30.0**	**28.4**
**conf_9**	-2837.3553	-2837.9760	-2836.1322	-2837.4024	-2837.3992	-2836.7970	-2836.7939	**30.2**	**28.5**
**conf_10**	-2837.3552	-2837.9757	-2836.1312	-2837.4020	-2837.3987	-2836.7954	-2836.7921	**31.2**	**29.6**
**conf_11**	-2837.3577	-2837.9773	-2836.1332	-2837.4029	-2837.3998	-2836.7951	-2836.7919	**31.4**	**29.7**
**conf_12**	-2837.3429	-2837.9651	-2836.1232	-2837.3914	-2837.3882	-2836.7908	-2836.7876	**34.1**	**32.5**
**conf_13**	-2837.3469	-2837.9686	-2836.1246	-2837.3940	-2837.3907	-2836.7903	-2836.7870	**34.4**	**32.8**

[7]Notations correspond to the notation of Table S1. The Gibbs free energies are relative to ***1-C$_{trans}$*** and **bf** reactant state

S15. Interaction energies and dispersion interaction

Computational studies were performed with the aim to compare the interaction energies of substrate **bf** and catalyst **1-C$_{trans}$** in the hydride transfer transition state structures of the major and minor pathways. The dispersion interaction was also estimated and compared by using several different computational approaches. For this studies the structures as optimized at the ωB97X-D/6-31G(d,p) + SDD (augmented by *f* polarization functions for the Ru centre) level of DFT were used.

S15.1 Computational details

The electronic energies E^{lb} computed at ωB97X-D/6-311++G(2d,p) + SDD (augmented by *2f,1g* polarization functions for the Ru centre) level of theory were used for calculating the interaction energies by using the Gaussian g09 program package. The total interaction energy between the molecules of the transition state complex were computed according to equation 2:

$$\Delta E_{DFT}^{int} = E_{complex}^{lb} - E_{cat}^{lb} - E_{subtr}^{lb} \quad (2)$$

as the relative energy of the complex and the sum of catalyst and substrate energy taken in the geometry of the transition state complexes.

For estimating the dispersion interaction different approaches were computed and compared. The D2 version of dispersion correction was collected as implemented in the framework of ωB97X-D in the Gaussian 09 package.[S5] Single point energy calculations were used by using the B3LYP-D3/6-311++G(2d,p) + SDD (augmented by *2f,1g* polarization functions for the Ru centre) level of theory in order to estimate the D3 dispersion term [S3, S10].

Further computations were performed with ORCA 5.0.1. program package [S7]. Ahlrick's type triple-ζ basis set with additional polarization functions def2-TZVPP were used [S4] for the Hartree-Fock London Dispersion (HFLD) method [S13] with "verytight" SCF setting for the HF part. For the correlation part the "state of the art" DLPNO-CC method [S14] with the auxiliary set def2-TZVPP/C was used. For the ruthenium the built-in ECP parameters def2-ECP (replacing 28 core electrons) were used as obtained from TURBOMOL 7.0.2 [S9].

The D3 [S6,S10] and D4 [20] dispersion interactions were also calculated at the B3LYP/def2-TZVPP level of theory by using ORCA 5.0.1. program.

S15.2 Total and dispersion interaction energies

The total interaction energy and the contribution of dispersion interaction calculated at different levels of theory are shown in Tabel S8.

Table S8: Interaction energies computed at ωB97X-D/6-311++G(2d,p) + SDD(+*2f,1g*) level and dispersion interactions calculated by different theoretical models for the hydride transfer transition state of **bf** hydrogenation reaction for the *re* and *si* pathways (the numbers are in kcal/mol).[8]

	ΔE^int^	***ΔE^disp^***	***ΔE^disp^***			
Struct	**ωB97X-D**	**ωB97X-D D2**	***HFLD***	***B3LYP D3***	***B3LYP D4***	***B3LYP-D3 D3***
**TS^{HT}(re)**	-44.9	-25.0	-47.7	-31.2	-29.4	-29.2
**TS^{HT}(si)**	-56.5	-27.8	-54.0	-35.3	-33.6	-32.7
**difference**	-11.6	-2.8	-6.2	-4.1	-4.2	-3.5

[8]Notations: The numbers in the column ωB97X-D/D2 are the D2 dispersion interactions computed as implemented in the ωB97X-D functional by using the Gaussian 09 program. The column HFLD represents the results computed for the HFLD method, by using the ORCA 5.0.1. program. The numbers in the columns B3LYP D3 and B3LYP D4 represent the dispersion interaction D3 and D4, respectively computed with the B3LYP functional by using the ORCA 5.0.1. The column titled B3LYP-D3/D3 contains the results for the dispersion interactions calculated by using the D3 method as implemented in the Gaussian 09 program.

According to our calculations the interaction energy of catalyst with the substrate **bf** in the **TS^{HT}(si)** structure is larger by 11.6 kcal/mol than in the **TS^{HT}(re)**. The dispersion interaction computed by using the D2 method shows that a part of the stabilizing forces in the pocket conformation is due to dispersion interactions. Moreover, all models used in our studies predicted larger dispersion interactions for the TS^{HT}**(si)** structure. Qualitatively the same trend is characteristic for the **TS^{HT}** of the reaction of **1-C** with the 2-methyl-thiobenzene (see Table S9).

Table S9: Interaction energies computed at ωB97X-D/6-311++G(2d,p) + SDD(+*2f,1g*) level and dispersion interactions calculated by different theoretical models for the hydride transfer transition state of 2-methyl-thiobenzene hydrogenation reaction for the *re* and *si* pathways (the numbers are in kcal/mol).[9]

	ΔE^{int}	***ΔE^{disp}***	***ΔE^{disp}***		
Struct	**ωB97X-D**	**ωB97X-D D2**	***HFLD***	***B3LYP D3***	***B3LYP D4***
**TS^{HT}(re)**	-46.7	-26.0	-52.5	-34.7	-32.3
**TS^{HT}(si)**	-54.8	-27.6	-55.6	-37.4	-34.9
**difference**	-8.1	-1.6	-3.2	-2.7	-2.7

[9]Notations correspond to the notations of Table S8.

S15.3 Truncated model of "pocket" complex

With the aim to evaluate the role of the "pocket" in the hydride transfer transition state structure, we considered a model system. In the truncated model the naphthyl ligand forming the pocket of the complex was substituted by an H atom and the model coordination complex as well as the model **TS^{HT}** was re-optimized. The free-energy diagram is presented in Figure S21. The reference state is the non-interacting optimized substrate and catalyst for the original system and the optimized substrate and truncated catalyst for the model system.

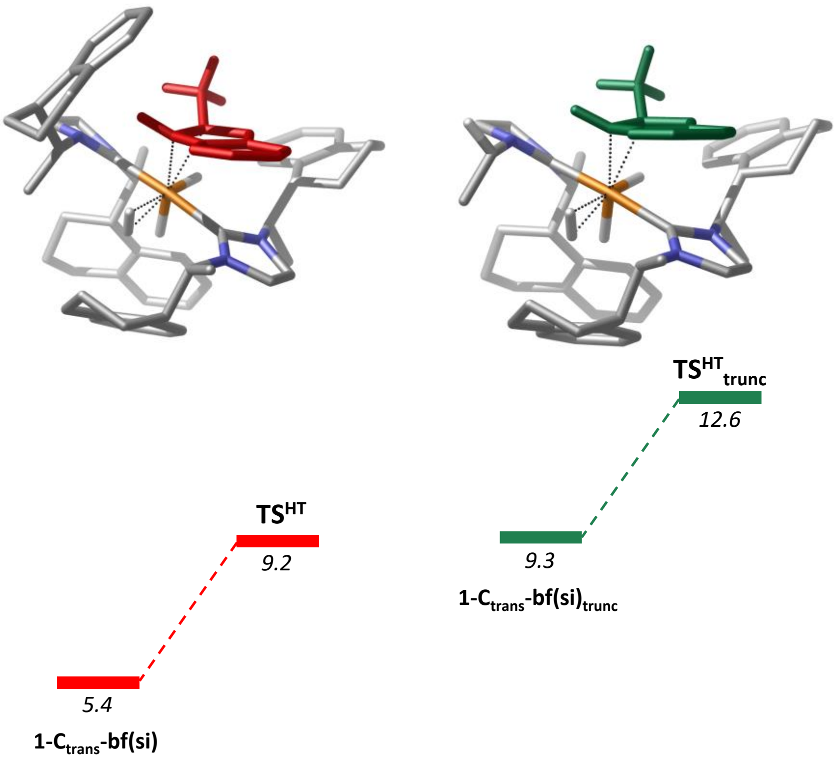

Figure S21: Optimized structures of **1-C$_{trans}$-bf(si)** and **1-C$_{trans}$-bf(si)$_{trunc}$** (upper part) and free energy diagrams showing the computed Gibbs free energies of coordination complexes and hydride transfer transition states for the original and truncated model systems relative to the un-interacting **bf** and the catalyst and truncated catalyst, respectively. (The numbers are in kcal/mol)

The results indicate that the lack of the "pocket" moiety shifts the model complexes higher in free-energy by 3-4 kcal/mol.

The interaction energies were computed according to the procedures presented in section S15.2. The strong interaction energy in the original system points to the presence of non-covalent attractive forces. The dispersion interaction estimated by the D2 model is weaker in the truncated model by 5.9 kcal/mol. The HFLD model predicts an even smaller dispersion energy for the model without the pocket by 7.8 kcal/mol. The computed energy values are in the Table S10.

Table S10: Interaction energies and dispersion interactions computed for the hydride transfer transition state of the original and truncated model system, respectively (the numbers are in kcal/mol). The dispersion interaction is calculated for the HFLD method, too.

Struct	ΔE^{int} ωB97X-D	ΔE^{disp} *D2/*ωB97X-D	ΔE^{disp} *HFLD*
**TS^{HT}(si)**	-56.5	-27.8	-54.0
**TS^{HT}(si)^{trunc}**	-52.3	-21.9	-46.2

All the results suggest that stabilization of the complexes arise due to attractive non-covalent interactions induced by the "pocket" configuration of the catalyst.

S15.4 Activation strain energy

For a deeper understanding of the stereoselectivity determining step of the reaction a study of the TS^{HT} structures was performed by using the Activations Strain Model. In this fragment-based model the relative energies of the system are related to the energies required to distort the reactants into the TS^{HT} geometries and their interaction energy in this structure. The electronic energies for this model were calculated by using the ωB97X-D/6-311++G(2d,p) + SDD (augmented by *2f,1g* polarization functions for the Ru centre) method. The energy diagram of this model and the related molecular structures are presented in Figure S22.

The total strain energy of the system is the sum of the distorted catalyst and **bf** energies. Our calculations show that the distortion energies are relatively large for the reactants (15-20 kcal/mol) and the total distortion energy is larger for the *si* transition state by 7.6 kcal/mol. However, the interaction energy between the reactants in structure **TS^{HT}(si)** is able to compensate the large strain energy. In turn, the interaction corresponding for the **TS^{HT}(re)** is weaker by 11.6 kcal/mol even if the distortion energies of the reactants were lower than for the *si* counterpart.

With the aim to understand the surprisingly high energy of the strained "pocket" conformation of the catalyst in the transition state structure, we compared the strained and the optimized structures of **1-C** as obtained from this conformation (see Figure S23). The optimized catalyst structure is a closed form while in the transition state structure the catalyst opens up to allow for the substrate coordination and the high interaction energy in this pocket compensates for the high distortion energy.

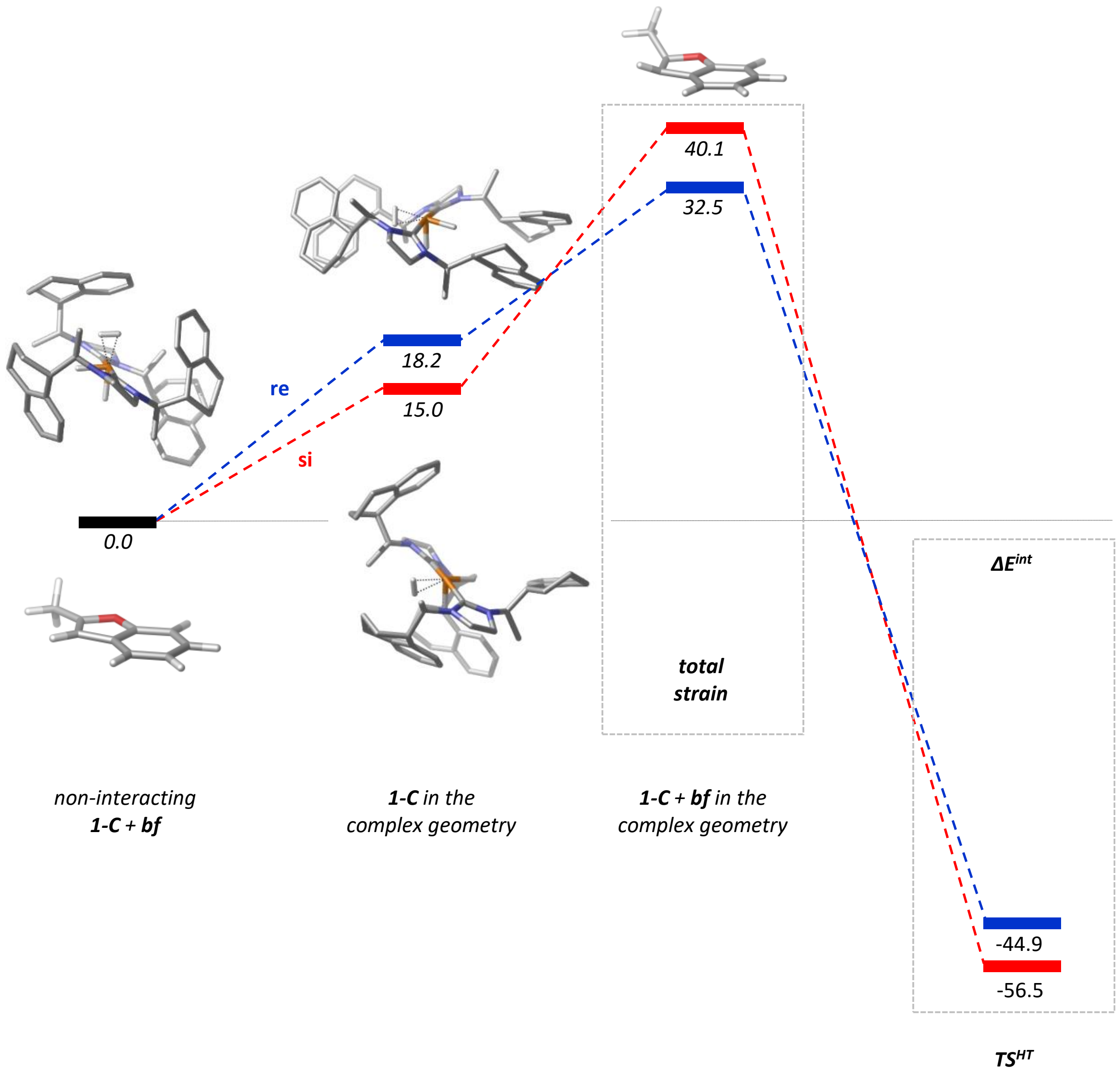

Figure S22: Diagram of the activation strain model performed for the hydride transfer transition states. The numbers are electronic energies (in kcal/mol) relative to the non-interacting **1-C$_{trans}$** + **bf**

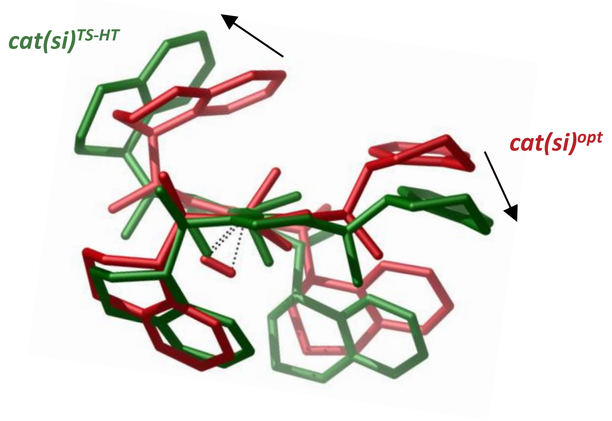

Figure S23: The strained (in green colour) and optimized (in red colour) structures of the "pocket" catalyst conformer.

All these results support the importance of the pocket conformation of the catalyst. Not only that the closed form of the catalyst is pre-organized to welcome the substrate, but the interaction with **bf** is stronger than for the "flat"-catalyst for the *trans(re)* pathway.

S16. Computed structures for different substrates

Assuming that the explored reaction mechanism and our findings on the stereoinducing factors can be extended to similar reactions, we performed computations for the rate- and selectivity determining **HT** step of the reaction for different substrates. Our aim was to investigate this step of the reaction by relying on an analogy to the **1-C$_{trans}$** + **bf** system and verify whether the energetic trend is transposed to other systems. We assessed this data knowing that the estimated energies might be further refined by possible stabilizing conformational changes. Performing a detailed conformational analysis and exploring the reaction pathway in detail for each substrate was outside of the scope for this work. However, the considered structures were fully optimized according to our computational protocol at the DFT level of theory.

The optimized structures of the systems are shown in Figure 7 of the manuscript, energy data are collected in Table S11.

Substrate **d** (2-methyl-5-phenylfuran) has two substituted double bonds susceptible to hydrogenation. The computations predict that the alkyl-substituted double bond coordinates first and is also reduced first (**TS^{HT}(si)^{Me}** and **TS^{HT}(re)^{Me}**).

Table S11: Energy data computed for the optimized HT transition states for si- and re-face attack of different substrates by using the range-separated ωB97X-D exchange-correlational functional. The number of the substrates is according to Figure 7 of the manuscript.[11]

Struct	***E^0***	***E^{lb}***	***G^0***	***$G^0_{toluene}$***	***$G^0_{n\text{-}hexane}$***	***$G_{toluene}$***	***$G_{n\text{-}hexane}$***	***ΔG_{tol}***	***$\Delta G_{n\text{-}hex}$***
**b**	-745.8391	-745.9538	-745.7284	-745.8510	-745.8503	-745.8521	-745.8514		
**TS^{HT}(si)**	-3160.3564	-3160.9842	-3159.1390	-3160.4064	-3160.4028	-3159.8138	-3159.8102	**14.0**	**12.6**
**TS^{HT}(re)**	-3160.3442	-3160.9722	-3159.1266	-3160.3918	-3160.3882	-3159.7992	-3159.7956	**23.1**	**21.7**
**c**	-614.5339	-614.6860	-614.3710	-614.5500	-614.5491	-614.5362	-614.5353		
**TS^{HT}(si)**	-3029.0683	-3029.7319	-3027.7929	-3029.1190	-3029.1154	-3028.5042	-3028.5005	**10.0**	**8.6**
**TS^{HT}(re)**	-3029.0584	-3029.7227	-3027.7874	-3029.1088	-3029.1051	-3028.4991	-3028.4953	**13.2**	**11.8**
**d**	-500.2451	-500.3703	-500.1001	-500.2579	-500.2572	-500.2350	-500.2343		
**TS^{HT}(si)^{Me}**	-2914.7656	-2915.4044	-2913.5163	-2914.8163	-2914.8126	-2914.2027	-2914.1991	**10.2**	**8.8**
**TS^{HT}(re)^{Me}**	-2914.7573	-2915.3958	-2913.5070	-2914.8075	-2914.8038	-2914.1928	-2914.1890	**16.4**	**15.1**
**TS^{HT}(si)^{Ph}**	-2914.7568	-2915.3959	-2913.5059	-2914.8066	-2914.8030	-2914.1918	-2914.1882	**17.0**	**15.6**
**TS^{HT}(re)^{Ph}**	-2914.7576	-2915.3956	-2913.5071	-2914.8069	-2914.8031	-2914.1915	-2914.1876	**17.2**	**16.0**

[11]Notations correspond to the notation of Table S1. The Gibbs free energies are relative to ***1-C$_{trans}$*** and the respective **substrate** reactant state.

S17. Energy data calculated by other methods

In order to assess the reliability of our calculations, the structures of the reaction steps have been re-optimized by using the B3LYP DFT functional corrected for dispersion by Grimme's D3 correction [S11]. Otherwise, the same working protocol was used: the 6-31G(d,p) basis set has been applied for optimization, entropy and solvent corrections, and the larger 311++G(2d,p) basis set for a more accurate electronic energy. Additional polarization functions were also added to the built-in SDD ECPs on the metal centre.

Table S12: Relative free energies computed using different DFT levels of theory.[12]

Struct	*ωB97X-D*	*B3LYP-D3*	*ωB97X-D/def2*
$\mathbf{1}\text{-}\boldsymbol{C_{trans}(si)}$	5.4	6.3	4.4
$\boldsymbol{TS^{HT}_{trans}(si)}$	9.2	11.4	7.5
$\boldsymbol{int^{1}_{trans}(si)}$	0.4	2.3	-1.6
$\boldsymbol{TS^{PT}_{trans}(si)}$	6.9	10.1	5.2
$\boldsymbol{int^{2}_{trans}(si)}$	3.5	4.2	1.8
$\mathbf{1}\text{-}\boldsymbol{C_{trans}(re)}$	8.3	10.0	7.6
$\boldsymbol{TS^{HT}_{trans}(re)}$	13.7	16.1	13.4
$\boldsymbol{int^{1}_{trans}(re)}$	2.4	6.4	1.5
$\boldsymbol{TS^{PT}_{trans}(re)}$	14.2	16.6	12.7
$\boldsymbol{int^{2}_{trans}(re)}$	0.5	10.8	0.2
$\mathbf{1}\text{-}\boldsymbol{C_{cis}(si)}$	9.3	11.8	7.8
$\boldsymbol{TS^{HT}_{cis}(si)}$	11.7	15.5	9.7
$\boldsymbol{int^{1}_{cis}(si`)}$	7.0	10.4	5.6
$\boldsymbol{TS^{PT}_{cis}(si)}$	27.3	30.5	21.3
$\mathbf{1}\text{-}\boldsymbol{C_{cis}(re)}$	11.4	14.0	10.4
$\boldsymbol{TS^{HT}_{cis}(re)}$	11.5	14.8	10.6
$\boldsymbol{int^{1}_{cis}(re)}$	6.4	10.2	5.3
$\boldsymbol{TS^{PT}_{cis}(re)}$	22.1	26.1	21.5

[12]The numbers are relative Gibbs free energies in kcal/mol. The reference is the sum of free reactants (***1-C**$_{trans}$* and ***bf***) calculated at the given level of theory.

The stationary points of the proposed reaction pathways have been re-optimized also by applying the ωB97X-D functional in conjunction with Ahlrich's basis sets def2-SVP/def2-TZVPP. These results show that the reaction pathways have the same characteristics for all theoretical methods used. All values are shown in Table S12.

S18. Energy data of calculated structures for the elementary steps of the reaction pathway

Table S13: Energy data of the optimized structures[13]

Struct	E^0	E^{lb}	G^0	$G^0_{toluene}$	$G^0_{n\text{-}hexane}$	$G_{toluene}$	$G_{n\text{-}hexane}$	ΔG_{tol}	$\Delta G_{n\text{-}hex}$
**H_2**	-1.1751	-1.1761	-1.1764	-1.1746	-1.1747	-1.1739	-1.1740		
**bf**	-422.8605	-422.9679	-422.7458	-422.8714	-422.8708	-422.8612	-422.8605		
**prod**	-424.0747	-424.1813	-423.9372	-424.0859	-424.0852	-424.0520	-424.0512		
$\mathbf{1\text{-}C_{trans}}$	-2414.4936	-2415.0104	-2413.4185	-2414.5454	-2414.5403	-2413.9840	-2413.9789	0.0	0.0
$\mathbf{1\text{-}C_{cis}}$	-2414.4889	-2415.0049	-2413.4089	-2414.5347	-2414.5307	-2413.9676	-2413.9637	10.2	9.5
$\mathbf{1\text{-}C'_{trans}}$	-2415.6938	-2416.2118	-2414.5992	-2415.7386	-2415.7349	-2415.1588	-2415.1552	-0.6	-1.5
$\mathbf{1\text{-}C'_{cis}}$	-2415.6866	-2416.2049	-2414.5909	-2415.7331	-2415.7294	-2415.1527	-2415.1491	3.2	2.4
$\mathbf{1\text{-}C''_{trans}}$	-2413.2925	-2413.8093	-2412.2347	-2413.3426	-2413.3384	-2412.7987	-2412.7944	7.2	6.6
$\mathbf{1\text{-}C''_{cis}}$	-2413.2898	-2413.8066	-2412.2311	-2413.3422	-2413.3372	-2412.7973	-2412.7923	8.0	7.9
$\mathbf{1\text{-}C_{trans} - bf(si)}$	-2837.3921	-2838.0121	-2836.1705	-2837.4410	-2837.4375	-2836.8364	-2836.8329	5.4	4.0
$\mathbf{1\text{-}C_{trans} - bf(re)}$	-2837.3901	-2838.0103	-2836.1661	-2837.4387	-2837.4349	-2836.8319	-2836.8281	8.3	7.0
$\mathbf{1\text{-}C_{cis} - bf(si)}$	-2837.3904	-2838.0100	-2836.1647	-2837.4394	-2837.4356	-2836.8304	-2836.8265	9.3	8.1
$\mathbf{1\text{-}C_{cis} - bf(re)}$	-2837.3896	-2838.0083	-2836.1632	-2837.4375	-2837.4338	-2836.8269	-2836.8231	11.4	10.2
$\mathbf{TS^{HT}_{trans}(si)}$	-2837.3839	-2838.0045	-2836.1637	-2837.4330	-2837.4294	-2836.8305	-2836.8268	9.2	7.8
$\mathbf{TS^{HT}_{trans}(re)}$	-2837.3780	-2837.9982	-2836.1585	-2837.42575	-2837.4221	-2836.8233	-2836.8197	13.7	12.3
$\mathbf{TS^{HT}_{cis}(si)}$	-2837.3840	-2838.0045	-2836.1626	-2837.4303	-2837.4273	-2836.8265	-2836.8235	11.7	9.9
$\mathbf{TS^{HT}_{cis}(re)}$	-2837.3849	-2838.0039	-2836.1631	-2837.43250	-2837.4289	-2836.8267	-2836.8231	11.5	10.2
$\mathbf{int^{1}_{trans}(si)}$	-2837.4013	-2838.0212	-2836.1780	-2837.4509	-2837.4472	-2836.8444	-2836.8408	0.4	-0.9
$\mathbf{int^{1}_{trans}(re)}$	-2837.4004	-2838.0201	-2836.1762	-2837.4488	-2837.4449	-2836.8412	-2836.8373	2.4	1.3
$\mathbf{int^{1}_{cis}(si)}$	-2837.3949	-2838.0154	-2836.1701	-2837.4412	-2837.4382	-2836.8339	-2836.8309	7.0	5.3
$\mathbf{int^{1}_{cis}(re)}$	-2837.3933	-2838.0131	-2836.1696	-2837.4417	-2837.4383	-2836.8349	-2836.8315	6.4	4.9
$\mathbf{TS^{PT}_{trans}(si)}$	-2837.3874	-2838.0070	-2836.1671	-2837.4380	-2837.4340	-2836.8342	-2836.8302	6.9	5.7
$\mathbf{TS^{PT}_{trans}(re)}$	-2837.3817	-2838.0001	-2836.1572	-2837.4317	-2837.4274	-2836.8225	-2836.8182	14.2	13.3
$\mathbf{TS^{PT}_{cis}(si)}$	-2837.3589	-2837.9787	-2836.1328	-2837.4109	-2837.4065	-2836.8016	-2836.7973	27.3	26.4
$\mathbf{TS^{PT}_{cis}(re)}$	-2837.3662	-2837.9863	-2836.1431	-2837.4159	-2837.4120	-2836.8100	-2836.8061	22.1	20.9
$\mathbf{int^{2}_{trans}(si)}$	-2837.3975	-2838.0170	-2836.1726	-2837.4479	-2837.4439	-2836.8395	-2836.8355	3.5	2.4
$\mathbf{int^{2}_{trans}(re)}$	-2837.3987	-2838.0171	-2836.1757	-2837.4520	-2837.4472	-2836.8444	-2836.8395	0.5	-0.1

[13]Notations correspond to the notation of Table S1. The Gibbs free energies of the catalyst configurations are relative to **1-C*trans*** (structures without substrate) or **1-C*trans*** + **bf** (structures with substrate).

S19. In Situ NMR Experiments

S19.1 Experimental Procedure

Inside an argon filled glovebox, 4.6 mg of **1-A** (5.4 µmol, synthesized as described in S15) was dissolved in 0.65 mL THF-d_8 (distilled over sodium/benzophenone and stored over 3 Å molecular sieves), yielding a clear, dark yellow solution. The solution was then transferred into a medium pressure J Young NMR tube and sealed. Subsequently, initial NMR spectra under argon atmosphere were recorded (t = 0 h).

Afterwards, 2 bar of H_2 pressure were applied to the J Young NMR tube, resulting in a partial H_2 pressure of 1 bar (due to the presence of 1 bar argon). After shaking to dissolve the added H_2, a slow colour change to orange could be observed. The reaction was monitored using ^{1}H NMR (Bruker AV 400, 400 MHz), showing consumption of dissolved H_2 as evidenced by a decrease in intensity for the H_2 signal at $\delta(^1H)$ = 4.55 ppm. Six hours after the first addition of H_2, the J Young NMR tube was re-pressurized with 2 bar H_2, and again after 36 h, shaking the NMR tube regularly to dissolve H_2. After the third pressurization, no further decrease of dissolved H_2 could be observed.

69.5 h after the first addition of H_2 no significant changes could be observed in the ^{1}H NMR spectra anymore. At this point, 0.15 mL of a 0.052 M solution of benzofuran in THF-d_8 (7.8 µmol benzofuran, ca. 1.5 equivalents relative to **1-A**) were added to the NMR tube while applying 2 bar of H_2 pressure. No significant colour change was observed upon addition of the substrate. Subsequently, the NMR tube was sealed and the reaction was monitored using ^{1}H NMR for an additional 119 h, especially following the hydride signals at $\delta(^1H)$ = −3.7 ppm and $\delta(^1H)$ = −3.8 ppm as well as the signals of 2,3-dihydrobenzofuran at $\delta(^1H)$ = 4.48 ppm and $\delta(^1H)$ = 3.15 ppm. After 119 h of reaction time, the colour of the reaction solution had changed to light orange.

S19.2 Results

Figure S19 shows the full ^{1}H NMR spectra recorded before (t = 0 h) and after addition of H_2 to the solution of **1-A**. In the hydride region it can be seen that 6.5 h after H_2 addition, there is a signal at $\delta(^1H)$ = −0.6 ppm and the signal at $\delta(^1H)$ = −14.9 ppm starts to appear. After 22.5 h, the signal at $\delta(^1H)$ = −0.6 ppm has disappeared and new signals at $\delta(^1H)$ = −9.6, −9.7 and −14.3 ppm have appeared. After 30 h, two new signals at $\delta(^1H)$ = −3.7 and −3.8 ppm have appeared. In subsequent spectra, the intensities of the $\delta(^1H)$ = −3.7, −3.8 and −9.7 ppm signals increase, while the intensity of the $\delta(^1H)$ = −14.9 ppm signal decreases.

After addition of benzofuran, the intensity of the $\delta(^1H)$ = −3.7 and −3.8 ppm signals decreases, accompanied by an appearance of two triplets at $\delta(^1H)$ = 4.48 and 3.15 ppm (see Figure S25). Using ^{1}H-^{1}H COSY, it could be confirmed that these signals are coupled (see Figure S26), suggesting that they belong to 2,3-dihydrobenzofuran. Regarding the other hydride signals, the $\delta(^1H)$ = −9.6 and −9.7 ppm signals increase over the course of the reaction, while the $\delta(^1H)$ = −14.9 ppm signal appears to remain mostly constant.

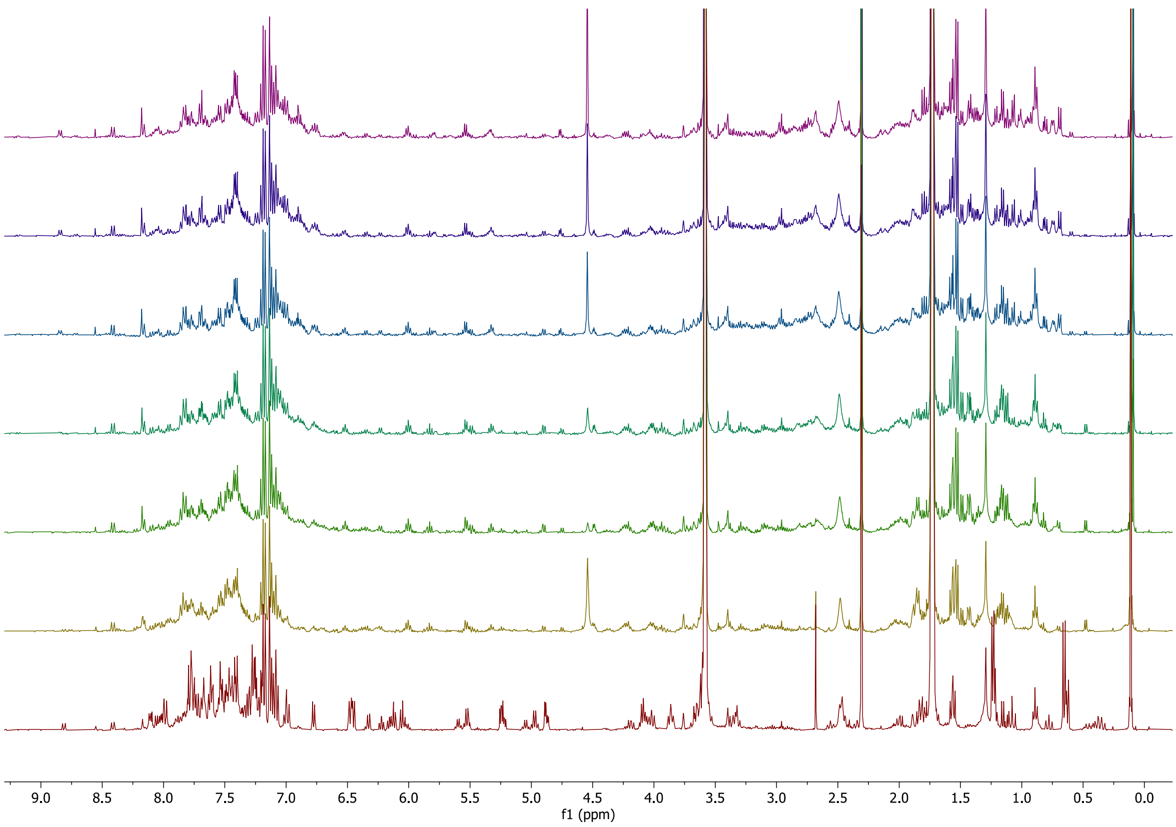

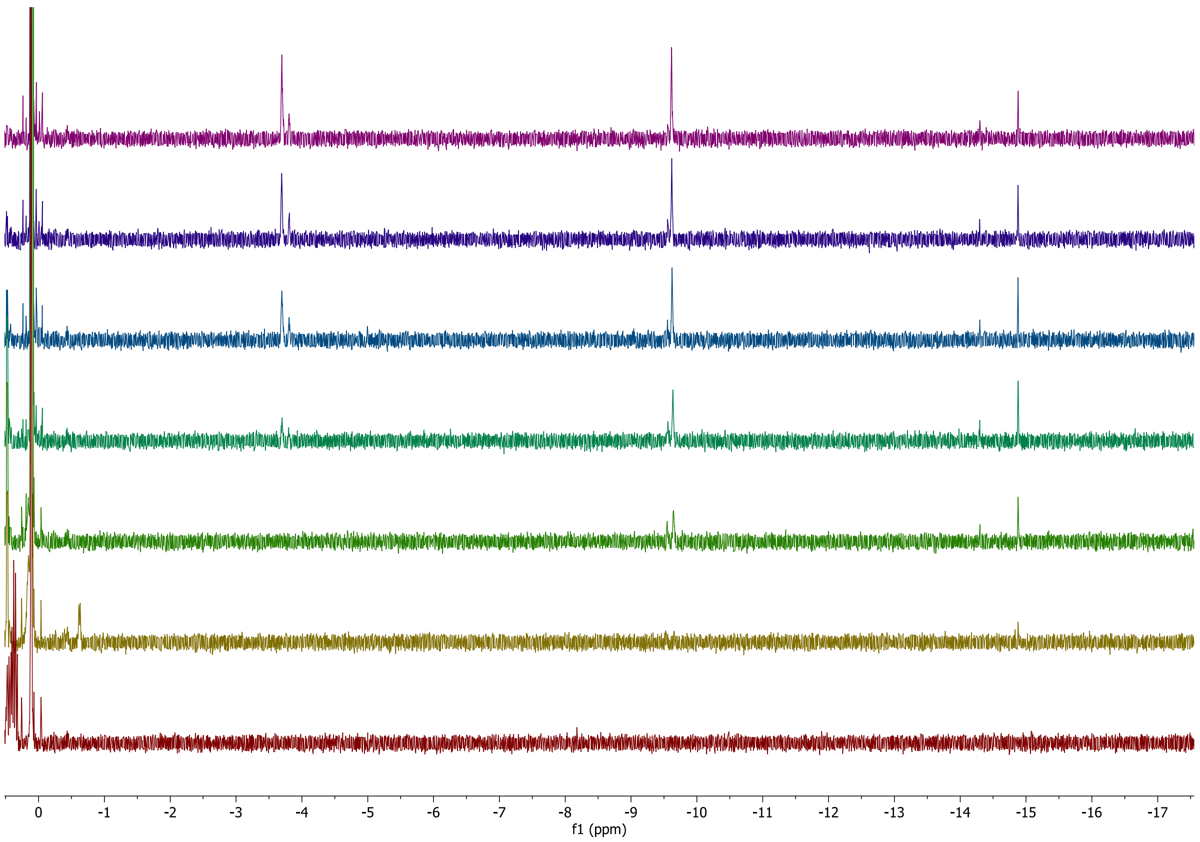

Figure S24: Stacked ^{1}H NMR spectra monitoring reaction of **1-A** with H_2, 0 to 9 ppm (top) and −17 to 0 ppm (bottom). For each stack of spectra, time progresses from bottom to top, with the timestamps being 0 h (argon atmosphere), 6.5 h, 22.5 h , 30 h, 45.5 h, 55 h, 69.5 h, relative to the time of first H_2 addition.

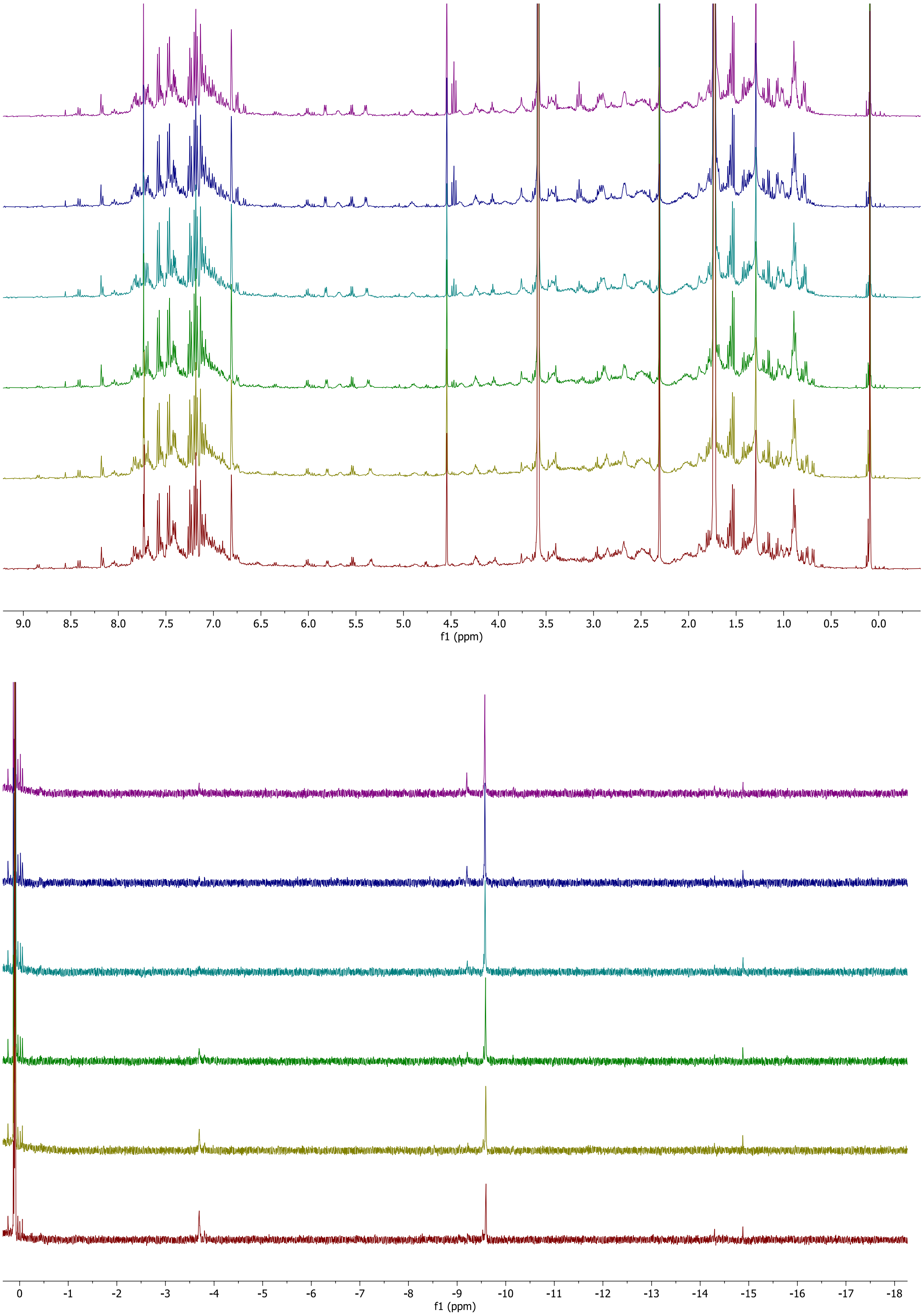

Figure S25 Stacked ^{1}H NMR spectra monitoring reaction after addition of benzofuran, 0 to 9 ppm (top) and −18 to 0 ppm (bottom). For each stack of spectra, time progresses from bottom to top, with the timestamps being 0.5 h, 1.5 h, 7 h, 24 h, 72 h, 119 h, relative to the time of benzofuran addition.

It should be noted that due to relatively poor mixing and H_2 diffusion inside the NMR tube, reactions involving H_2 are expected to proceed more slowly compared to a properly stirred reaction flask. Hence, hydride formation as well as substrate hydrogenation occur on timescales which are significantly slower (69.5 and 119 h, respectively) than the corresponding reactions in stirred flasks. Furthermore, use of THF as the solvent instead of less coordinating hexane could also slow the reaction due to coordination of THF to the ruthenium complexes.

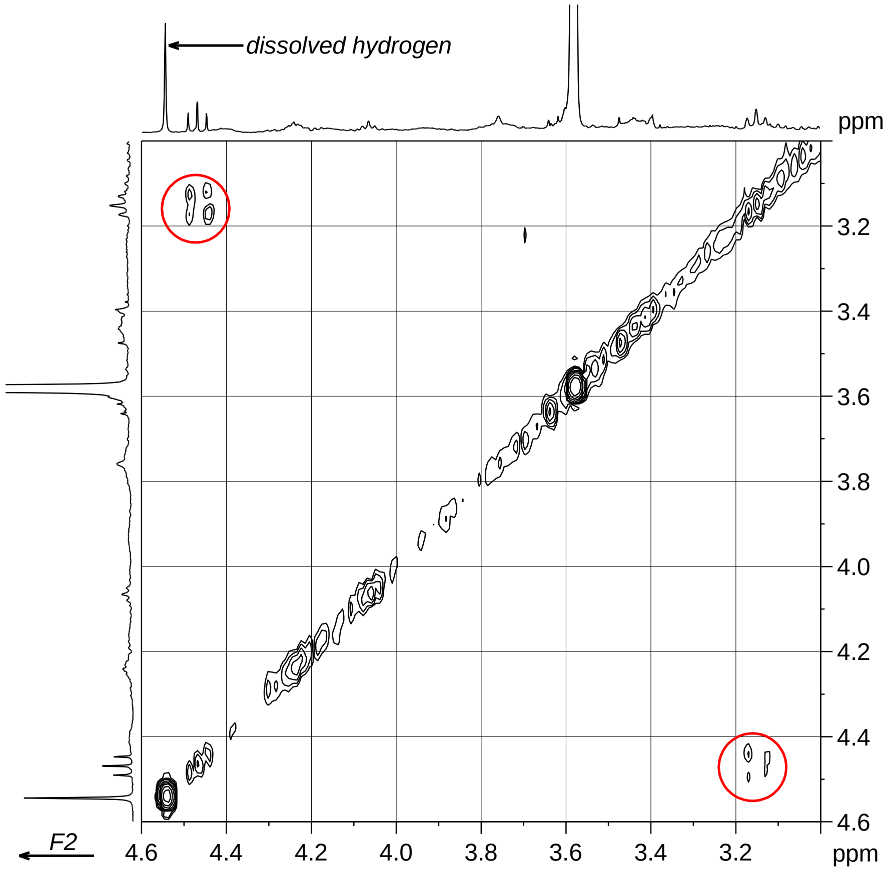

Figure S26 1H-1H COSY spectrum showing cross peak of aliphatic 2,3-dihydrobenzofuran signals, recorded 72 h after addition of benzofuran.

S20. Calculated NMR spectra

Magnetic shielding tensors have been computed by using the Gauge-Independent Atomic Orbital (GIAO) method as implemented in the Gaussian 09 software package. The calculations were performed by using the ωB97X-D/6-31G(d,p) level of theory by using THF as solvent. The reference state of TMS has been calculated at the same level of theory. The results obtained for the most stable catalyst forms, which are expected to be suitable for experimental identification, are presented in Figure S7.

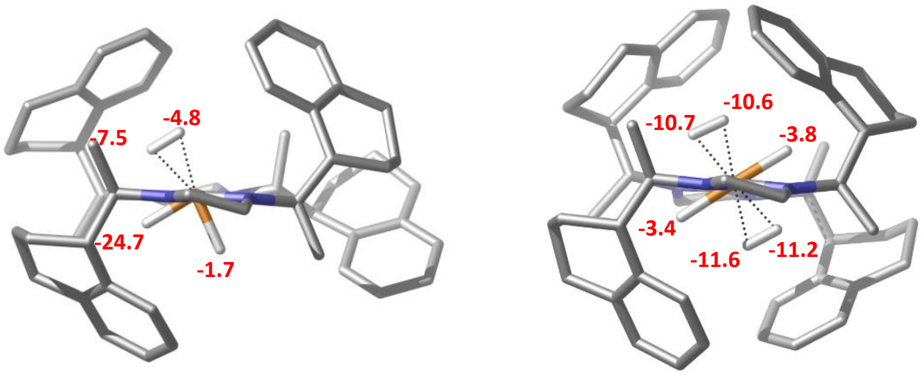

1-C$_{trans}$ **1-C′$_{trans}$**

Figure S27: ^{1}H-NMR shift values (in red) for the optimized structures of the catalyst **1-C*$_{trans}$*** and **1-C′*$_{trans}$*** in ppm.

As the chemical shifts were measured experimentally by using THF as the solvent, the presence of solvent molecules should also be taken into account in the calculations. In order to account for the effect of the solvent on the chemical shifts, coordination of a solvent molecule to the ruthenium centre has also been studied by explicitly considering the THF molecule. Therefore, the stability of the coordinated complex **1-C*$_{trans}$*-THF** is referenced to the loosely bound **1-C*$_{trans}$*···THF** system. (see Figure S28.)

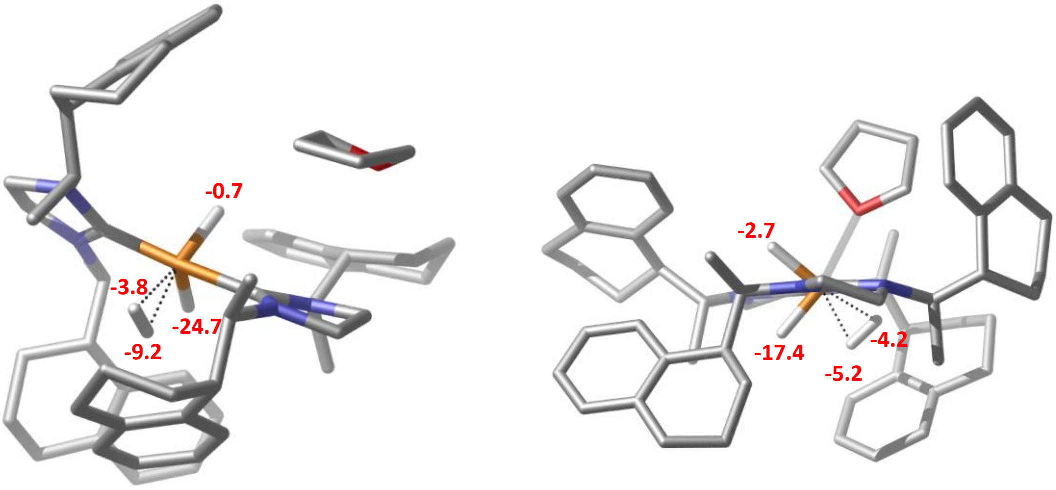

2-C$_{trans}$···THF **2-C$_{trans}$-THF**

Figure S28: ^{1}H-NMR shift values for the reference structure **1-C*$_{trans}$*···THF** and the coordinated complex **1-C*$_{trans}$*-THF** in ppm.

References

[S1] Y. Minenkov, A. Singstad, G. Occhipintia and V. R, Jensen, *Dalton Trans.,* 2012, **41**, 5526.

[S2] Gaussian 09, Revision E.01, M. J. Frisch, G. W. Trucks, H. B. Schlegel, G. E. Scuseria, M. A. Robb, J. R. Cheeseman, G. Scalmani, V. Barone, B. Mennucci, G. A. Petersson, H. Nakatsuji, M. Caricato, X. Li, H. P. Hratchian, A. F. Izmaylov, J. Bloino, G. Zheng, J. L. Sonnenberg, M. Hada, M. Ehara, K. Toyota, R. Fukuda, J. Hasegawa, M. Ishida, T. Nakajima, Y. Honda, O. Kitao, H. Nakai,

T. Vreven, J. A. Montgomery, Jr., J. E. Peralta, F. Ogliaro, M. Bearpark, J. J. Heyd, E. Brothers, K. N. Kudin, V. N. Staroverov, T. Keith, R. Kobayashi, J. Normand, K. Raghavachari, A. Rendell, J. C. Burant, S. S. Iyengar, J. Tomasi, M. Cossi, N. Rega, J. M. Millam, M. Klene, J. E. Knox, J. B. Cross, V. Bakken, C. Adamo, J. Jaramillo, R. Gomperts, R. E. Stratmann, O. Yazyev, A. J. Austin, R. Cammi, C. Pomelli, J. W. Ochterski, R. L. Martin, K. Morokuma, V. G. Zakrzewski, G. A. Voth, P. Salvador, J. J. Dannenberg, S. Dapprich, A. D. Daniels, O. Farkas, J. B. Foresman, J. V. Ortiz, J. Cioslowski, and D. J. Fox, Gaussian, Inc., Wallingford CT, 2013.

[S3] B3LYP functional: A. D. Becke, *J. Chem. Phys.,* 1993**, 98**, 5648-52.

[S4] Ahlrichs basis sets: a) F. Weigend and R. Ahlrichs, *Phys. Chem. Chem. Phys.,* 2005, **7**, 3297-305; b) F. Weigend, “Accurate Coulomb-fitting basis sets for H to Rn,” *Phys. Chem. Chem. Phys.*, 2006, **8***,* 1057-65.

[S5] D2 dispersion correction: S. Grimme, *J. Comput. Chem.*, 2006, **27**, 1787.

[S6] D3 dispersion correction S. Grimme, J. Antony, S. Ehrlich and H. Krieg, *J. Chem. Phys*., 2010, **132***,* 154104.

[S7] ORCA 5.0.1 program: a) F. Neese, “The ORCA program system” Wiley Interdisciplinary Reviews: Computational Molecular Science, 2012, Vol. 2, Issue 1, Pages 73–78; b) F. Neese, “Software update: the ORCA program system, version 4.0” Wiley Interdisciplinary Reviews: Computational Molecular Science, 2017, Vol. 8, Issue 1, p. e1327.

[S8] def2-TZVPP/C auxiliary basis set: A. Hellweg, C. Hattig, S. Hofener and W. Klopper, *Theor. Chem. Acc.*, 2007, **117**, 587.

[S9] def2-ECP pseudopotential: D. Andrae, U. Haeussermann, M. Dolg, H. Stoll, H. Preuss, *Theor. Chim. Acta,* 1990, **77,** 123-141.

[S10] atom-pairwise dispersion correction with the Becke-Johnson damping scheme (D3BJ): S. Grimme, S. Ehrlich, L. Goerigk, *J Comput Chem*, 2011, **32**, 1456–1465.

[S11] S.Grimme, J. Antony, S. Ehrlich and H. Krieg, *J.Chem.Phys*., 2010, **132**, 154104

[S12] E. Caldeweyher, C. Bannwarth, S. Grimme, *J.Chem.Phys*., 2017, **147**, 034112.

[S13] HFLD method: A. Altun, F. Neese, G. Bistoni, *J. Chem. Theory Comput.*, 2019, **15**, 5894.

[S14] DNPLO-CCSD(T) method: C. Riplinger and F. Neese, *J. Chem. Phys.,* 2013, **138**, 034106.

[S15] D. Paul, B. Beiring, M. Plois, N. Ortega, S. Kock, D. Schlüns, J. Neugebauer, R. Wolf and F. Glorius, *Organometallics*, 2016, **35**, 3641–3646.

S21. Cartesian coordinates of the calculated structures

ωB97X-D/6-31G(d,p) optimized geometries in standard XYZ format are shown. First line indicates the number of atoms, second line is the molecule name, the units for the Cartesian coordinates are Ångströms.

2
H₂
H 0.000000 -0.000000 0.132685
H 0.000000 -0.000000 0.875315

18
bf
C -0.014673 0.000000 -0.137298
C -0.154667 0.000000 1.255255
C 0.999125 -0.000000 2.049415
C 2.288358 -0.000000 1.536574
C 2.402836 -0.000000 0.152029
C 1.265765 -0.000000 -0.672520
C -1.254247 0.000000 2.187703
O 0.663236 -0.000000 3.368027
H 3.154449 -0.000000 2.188381
H 3.389252 -0.000000 -0.300280
H 1.394595 -0.000000 -1.750235
H -0.886813 0.000000 -0.783325
C -0.705350 0.000000 3.426129
H -2.310598 0.000000 1.962403
C -1.294545 0.000000 4.795660
H -0.497950 0.000000 5.541481
H -1.917876 0.884142 4.956009
H -1.917876 -0.884142 4.956009

20

Prod
C 0.010794 -0.099125 -0.133129
C -0.052250 0.011853 1.245685
C 1.122235 0.101326 1.990618
C 2.375755 0.083249 1.398457
C 2.426365 -0.031241 0.007917
C 1.263155 -0.125263 -0.753819
C -1.209623 0.120925 2.204078
O 0.904071 0.213250 3.325868
H 3.274540 0.152522 2.000410
H 3.393227 -0.052261 -0.485535
H 1.329842 -0.218238 -1.832727
H -0.897875 -0.165467 -0.724596
C -0.499955 -0.035485 3.569016
H -1.975690 -0.645082 2.057847
C -0.980631 0.902730 4.655962
H -0.397073 0.768875 5.569964
H -0.881498 1.941690 4.328476
H -2.032627 0.706689 4.883957
H -1.697952 1.100629 2.122822
H -0.579778 -1.077286 3.907419

109
1-A
C 0.033758 0.565606 -0.654740
C -0.398394 2.865660 -0.710515
H 0.015120 3.449983 0.118797
H -1.253414 3.404879 -1.127249
C 0.659692 2.516170 -1.765928
H 0.264269 2.620350 -2.784962
H 1.565140 3.122287 -1.676579
C -1.756139 1.264907 0.785510
H -1.316194 1.587962 1.740791
C -1.964571 -0.255825 0.802055
C 1.845575 0.355613 -2.325829
H 2.601867 1.090665 -2.611682
C 1.152702 -0.145089 -3.599399
H 0.393116 -0.883189 -3.336251
H 1.883720 -0.617742 -4.261881
H 0.673097 0.675997 -4.142883
C -3.022803 2.076330 0.583623
C -3.459668 2.884259 1.605570
H -2.879724 2.928798 2.524249
C -4.634523 3.661763 1.493655
H -4.949810 4.281241 2.327635
C -5.358788 3.639950 0.334033
H -6.259039 4.239515 0.229796
C 2.576751 2.642552 1.191941
C -5.684431 2.813316 -1.969738
H -6.575740 3.430895 -2.043102
C -5.288113 2.036391 -3.026197
H -5.861192 2.031541 -3.948251
C -4.132624 1.232836 -2.909924
H -3.826743 0.606910 -3.743171
C -3.397642 1.222941 -1.751770
H -2.528019 0.581185 -1.680771
C 3.496894 2.220848 0.189994
H 3.695335 1.166542 0.036387
C 1.364307 4.487629 2.271355
H 1.167668 5.552750 2.354498
C -3.772328 2.029887 -0.639859
C -4.944482 2.832077 -0.758369
C 1.889756 1.724971 2.054660
C 0.366030 -1.365593 1.770633
C 0.103042 -2.092113 3.966608
H 0.293118 -3.012228 4.523263
H -0.754931 -1.576048 4.424295
C 0.965164 2.205678 2.947402
H 0.415557 1.506063 3.569580
C 1.335351 -1.193955 3.883014
H 1.350655 -0.410842 4.644858
H 2.255127 -1.783702 3.972548
C -1.291337 -3.160111 2.120664
H -2.191950 -2.748259 2.599306
C -1.124967 -4.613176 2.567826
H -0.203665 -5.037243 2.158227
H -1.967539 -5.205676 2.201918
H -1.094498 -4.707754 3.658060
C 2.224588 0.248789 1.957172
H 2.249421 -0.009085 0.900365
C 3.607111 -0.056968 2.549580
H 3.642896 0.154740 3.622673
H 4.365131 0.564260 2.071334
H 3.869360 -1.103428 2.371679
C -1.465069 -3.001216 0.615879
C -0.442208 -3.470719 -0.279055
C -0.528980 -2.937964 -1.601622
C -1.628701 -2.089165 -1.874130
C -4.158103 -2.258730 -1.851606
H -4.214957 -1.833518 -2.849698
C -5.326613 -2.612420 -1.172795
H -6.292015 -2.458295 -1.644686
C -5.251816 -3.154383 0.102510
H -6.157788 -3.429898 0.633372
C 0.696150 3.589698 3.058482
H -0.041976 3.933770 3.776281
C -4.007551 -3.329128 0.717002
H -3.964789 -3.741810 1.721470
C -2.845615 -2.954517 0.057049
C 4.124494 3.126367 -0.626599
H 4.815617 2.768534 -1.383901
C -2.919660 -2.431074 -1.248579
C 2.524147 -0.772098 -1.566286
C 1.806476 -1.556076 -0.669360
C 2.566378 -2.547903 0.034531
H 2.061885 -3.159255 0.778323
C 3.908270 -2.742326 -0.136335
H 4.435931 -3.497912 0.442178
C 6.025567 -2.147347 -1.261285
H 6.531590 -2.908263 -0.672354
C 6.721714 -1.393957 -2.169724
H 7.787208 -1.543748 -2.311820
C 6.034612 -0.424998 -2.931729
H 6.574786 0.161761 -3.668834
C 4.687399 -0.222579 -2.759816
H 4.201610 0.514111 -3.389459
C 2.312200 4.036538 1.314046
C 3.930475 -0.969965 -1.809094
C 4.633707 -1.961140 -1.067377
C 2.977674 4.949697 0.453061
H 2.764859 6.010167 0.557680
C 3.867491 4.510091 -0.492454
H 4.369495 5.219608 -1.142644
N -0.790489 1.542567 -0.254346
N 0.946399 1.110391 -1.473819
N -0.157506 -2.343461 2.557185
N 1.211109 -0.634793 2.532205
Ru -0.275579 -1.342192 -0.109239
H -2.150913 -0.555390 1.840596
H -2.873721 -0.505989 0.253360
H 0.263026 -3.122548 -2.319111
H 0.383430 -4.105409 0.018905
H -1.666618 -1.593923 -2.841632

111
1-B
Ru 1.431704 -0.280000 -0.687458
C 3.285414 0.246095 -1.690702
C 2.181727 0.789602 -2.381267
C 3.437589 0.664153 -0.324471
C 1.382657 1.689212 -1.535256
H 3.914617 -0.514441 -2.138207
H 1.961617 0.546283 -3.415859
H 4.203012 0.166695 0.262304
C 3.231469 2.098988 -0.040044
C 2.178408 2.687398 -0.761507
C 3.992776 2.869539 0.828992
H 4.799624 2.406236 1.391513
C 3.718930 4.234250 0.978905
H 4.302629 4.829557 1.674977
C 2.717324 4.829076 0.223921
H 2.516973 5.892301 0.320734
C 1.957451 4.055403 -0.664382
H 1.176333 4.530342 -1.252650
C 2.680257 -2.923623 -2.115410
C 1.719309 -2.510844 -1.113251
C 3.995206 -3.018455 -1.830070
C 2.138907 -2.234792 0.213308
H 2.310636 -3.190222 -3.102489
H 0.730813 -2.915703 -1.250880
H 4.709752 -3.348138 -2.579538
C 4.489528 -2.673714 -0.512676
C 3.584343 -2.280989 0.501591
C 5.868118 -2.708541 -0.235054
H 6.550758 -3.009371 -1.025939
C 4.110574 -1.914394 1.753901
H 3.434138 -1.537998 2.513786
C 5.469461 -1.956264 2.009216
H 5.845072 -1.655016 2.982542
C 6.359634 -2.362975 1.008921
H 7.427105 -2.392703 1.203625
C 0.767201 -0.175402 1.247971
N 0.943107 -1.271373 2.047952
C 0.211625 -1.179679 3.299776
C 0.019717 0.330828 3.411443
N 0.170427 0.761682 2.017510
H -0.743413 -1.720390 3.232508
H 0.777694 -1.595512 4.136741
H 0.797077 0.783853 4.038042
H -0.956459 0.615217 3.814670
C -0.521131 -0.206742 -1.422537
N -1.675297 -0.953817 -1.395945
C -2.808099 -0.274242 -2.036740
C -2.286128 1.142307 -2.229913
N -0.861218 0.970749 -1.997037
H -3.056548 -0.752019 -2.992874

H -3.692506 -0.314239 -1.396114
H -2.704510 1.841418 -1.496548
H -2.484803 1.533505 -3.231335
C 0.023442 2.124407 -2.032943
C 1.160258 -2.525611 1.345121
C -0.158309 2.119911 1.610183
H 0.418839 2.295892 0.703430
C 0.335359 3.102090 2.682957
H 0.177157 4.136362 2.383458
H 1.409148 2.959751 2.832484
H -0.189165 2.956922 3.632214
C -1.644892 2.268707 1.273252
C -2.165478 3.478437 0.700667
C -3.886756 1.300847 1.182128
C -3.561271 3.567361 0.415302
C -2.507552 1.219347 1.473140
C -4.409684 2.459192 0.678069
H -2.115702 0.285515 1.847888
H -4.514525 0.433503 1.364565
C -1.678757 -2.416277 -1.466413
H -0.983983 -2.763207 -0.698657
C -1.160055 -2.878685 -2.832889
H -0.179994 -2.431551 -3.008885
H -1.815782 -2.554365 -3.646079
H -1.068053 -3.967692 -2.876865
C -3.056583 -2.967560 -1.109182
H -3.454490 -3.754791 -3.052437
C -3.826941 -3.616457 -2.044464
C -4.876873 -3.307764 0.521974
C -5.112093 -4.120554 -1.739226
C -3.576501 -2.808846 0.218701
C -5.630262 -3.965335 -0.485157
H -0.385013 2.862478 -1.330358
C 0.034594 2.769487 -3.422900
H 0.312504 2.037998 -4.187132
H 0.770587 3.577608 -3.441094
H -0.938413 3.195872 -3.687565
H 0.207695 -2.816701 0.873946
C 1.532345 -3.676345 2.279419
H 1.718537 -4.569227 1.677307
H 0.707238 -3.900304 2.963494
H 2.428684 -3.476270 2.867839
H -5.469196 2.543351 0.452755
H -5.681672 -4.626733 -2.511949
H -6.618641 -4.343276 -0.239427
C -5.404826 -3.142609 1.828367
C -2.849320 -2.167307 1.257240
H -1.867361 -1.759259 1.041617
C -4.681139 -2.512021 2.805335
H -5.093211 -2.386377 3.801323
C -3.389856 -2.023635 2.511495
H -2.830819 -1.511377 3.288833
C -1.347939 4.586369 0.342335
H -0.277532 4.534188 0.493898
C -4.081484 4.752441 -0.169326
C -1.877253 5.710509 -0.238102
H -1.222477 6.532667 -0.510069
C -3.263903 5.803982 -0.488238
H -3.674533 6.700989 -0.940718
H -5.148179 4.803808 -0.370636
H -6.398552 -3.527763 2.039566

123

1-D

C -0.623062 0.387889 0.868104
C 0.132793 1.334179 2.874813
C -1.398289 1.370874 2.857475
C 1.786172 0.357173 1.191615
C 1.709373 -0.362342 -0.157551
C -3.087064 0.675649 1.066415
C -4.061581 1.669572 1.691037
C 2.607729 -0.404603 2.263948
C 1.880768 -1.691081 2.683305
C 2.806501 -2.667808 3.399233
C 3.927220 -3.089814 2.453809
C 1.018160 4.174750 -0.212538
C 5.961567 -2.051886 1.406562
C 6.673041 -0.986840 0.873305
C 6.073226 0.268559 0.812316
C 4.772894 0.429807 1.269250
C 2.219913 3.790528 -0.819314
C -0.223116 4.907989 1.900957
C 4.035936 -0.642246 1.785938
C 4.650706 -1.898999 1.869258
C -0.246507 4.325182 -1.050055
C 0.158928 0.853730 -2.203359
C 1.200891 1.531693 -4.175061
C -1.250989 5.240885 -0.337838
C 0.326417 2.666932 -3.645079
C 1.272949 -0.933341 -3.468704
C 1.134854 -1.456955 -4.899510
C -0.859680 2.942508 -1.402287
C -2.274985 3.010748 -1.986303
C 0.577625 -1.795182 -2.426973
C -0.861394 -1.914384 -2.464684
C -1.441927 -2.405936 -1.256060
C -0.523598 -2.754821 -0.219996
C 1.335175 -4.463550 0.061046
C 2.532623 -5.017314 -0.407707
C 3.114181 -4.530048 -1.568083
C -1.503383 4.751428 1.086123
C 2.499175 -3.490209 -2.276313
C 1.316349 -2.932619 -1.813105
C 3.397087 3.666374 -0.094870
C 0.726987 -3.425024 -0.629795
C -3.493211 -0.810435 1.255473
C -3.494274 -1.251487 2.728051
C -4.317036 -2.516724 2.948970
C -5.768179 -2.246313 2.558311
C -7.020576 -2.034138 0.382216
C -7.117873 -1.675432 -0.955436
C -6.031044 -1.068881 -1.579319
C -4.878117 -0.805456 -0.851197
C 1.016720 4.434093 1.167290
C -4.782879 -1.127371 0.510226
C -5.864801 -1.775112 1.124119
C 2.208934 4.306956 1.888541
C 3.393603 3.930699 1.272685
N 0.430729 0.569874 1.677268
N -1.713300 0.915316 1.500548
N 0.738454 0.424546 -3.354239
N 0.020068 2.196697 -2.293369
Ru -0.335326 -0.574468 -0.907348
H 0.585385 2.330048 2.802460
H 0.527596 0.846693 3.771627
H -1.825333 0.698521 3.609710
H -1.795058 2.374146 3.039753
H 2.221172 1.357717 1.063311
H -3.076546 0.865116 -0.007526
H -4.116538 1.593585 2.780902
H -5.061814 1.480087 1.293717
H -3.783299 2.697046 1.438843
H 1.025530 -1.432363 3.317660
H 3.232225 -2.197236 4.295891
H 4.647049 -3.738135 2.966164
H 6.427268 -3.032386 1.473771
H 7.689713 -1.130604 0.520035
H 6.617736 1.119339 0.412518
H 4.313416 1.411180 1.219022
H 2.234655 3.593797 -1.885838
H -0.087435 5.969422 2.149495
H 1.049548 1.348395 -5.240889
H 2.270938 1.722595 -3.999832
H -0.833789 6.254920 -0.297379
H 0.840995 3.630773 -3.641840
H -0.594188 2.768475 -4.236214
H 2.341267 -0.897384 -3.205261
H 1.473365 -2.495036 -4.941638
H 1.732563 -0.879595 -5.612689
H 0.088396 -1.427393 -5.217810
H -0.903136 2.339769 -0.495349
H -3.001550 3.302933 -1.223615
H -2.554705 2.015839 -2.344626
H -2.351131 3.722350 -2.816406
H 0.882700 -4.835906 0.977196
H 3.008762 -5.821220 0.146115
H 4.049933 -4.946498 -1.927814
H -1.804608 3.696628 1.065892
H 2.970395 -3.113596 -3.180168
H 4.312121 3.362431 -0.593334
H -3.917637 -3.335454 2.337023
H -6.381288 -3.144453 2.687671
H -7.852152 -2.540330 0.866892
H -8.024826 -1.888176 -1.512883
H -6.076938 -0.809525 -2.632663
H -4.021984 -0.359678 -1.351906
H 2.199962 4.517486 2.955747
H 4.306343 3.839579 1.853356
H 2.343851 0.176694 -0.870640
H 2.145735 -1.361106 -0.071402
H -2.519167 -2.461673 -1.141042
H -1.474545 -1.575140 -3.292501
H -0.938870 -3.040401 0.744823
H -2.458649 -1.405986 3.050090
H -1.797312 0.050002 -1.163847
H 1.467913 -2.181032 1.794411
H 2.245491 -3.547575 3.732770
H 3.497310 -3.678861 1.634336
H 2.686985 0.241096 3.153964
H -3.914746 -0.465489 3.369020
H -4.254996 -2.833401 3.995129
H -6.185409 -1.485719 3.233455
H -2.697866 -1.368168 0.752319
H 0.028045 4.816151 -1.993110
H -2.180035 5.312228 -0.907798
H -2.318922 5.310004 1.556241

H -0.306804 4.384973 2.861019

129
1-F
C -0.543404 -1.078087 1.995545
C 0.033430 -2.670014 3.618654
H 0.206091 -2.598130 4.696637
H 0.633517 -3.505516 3.230845
C -1.447211 -2.833410 3.257638
H -1.716653 -3.868603 3.026311
H -2.102185 -2.482122 4.066941
C 1.693600 -0.827605 2.915894
H 1.939478 -0.585879 3.960190
C 1.615574 0.463331 2.074514
C -2.785528 -1.880161 1.311576
H -2.585789 -1.123533 0.553799
C -3.949183 -1.378890 2.166924
H -4.231753 -2.103143 2.939751
H -4.828400 -1.189751 1.547156
H -3.664314 -0.441604 2.655377
C 0.438825 2.353285 0.031369
C 1.613773 4.059637 -1.092577
H 1.612409 4.326463 -2.154249
H 2.583433 4.349985 -0.670679
C 0.449893 4.692063 -0.322128
H 0.773247 5.485041 0.360839
H -0.302953 5.122568 -0.997407
C -1.159099 3.732743 1.372207
H -0.952206 4.694910 1.864009
C -1.056470 2.674564 2.468302
H -1.393200 1.664417 2.138834
H -1.709225 2.924185 3.307504
H -0.034976 2.632801 2.846548
N 0.364407 -1.427152 2.937394
N -1.564771 -1.968935 2.087077
N 1.377447 2.630277 -0.905103
N -0.084438 3.553998 0.412462
Ru -0.153139 0.570914 0.895787
H 1.670932 1.320444 2.765444
H 2.523432 0.536972 1.470216
C 2.376869 1.656302 -1.320731
H 1.979560 0.707445 -0.959625
C 3.720690 1.911002 -0.630858
H 4.372535 1.041504 -0.731653
H 4.247288 2.771194 -1.061509
H 3.569607 2.089346 0.436407
C 0.372195 0.266441 -3.195880
C 1.170883 1.509977 -3.590448
C 2.518177 1.578745 -2.860274
C 1.205600 -1.033409 -3.152259
H -0.466416 0.142383 -3.889016
H -0.064532 0.437532 -2.208760
H 0.573982 2.402214 -3.369978
H 1.373668 1.511977 -4.668156
H 3.059195 2.472888 -3.201989
H 1.057472 -1.497478 -2.169715
H 0.827508 -1.757025 -3.883146
C 2.695615 -0.878334 -3.369777
C 3.337085 0.358969 -3.227958
C 4.718274 0.442765 -3.420791
C 5.472991 -0.682795 -3.727660
C 4.838845 -1.915981 -3.860179
C 3.462817 -2.002206 -3.690275
H 5.204995 1.409817 -3.320186
H 6.546074 -0.598265 -3.868397
H 5.413396 -2.804665 -4.102551
H 2.965188 -2.962756 -3.803752
H 0.556503 -0.388902 -0.103862
H -1.752074 0.633073 0.270525
C 2.764351 -1.886266 2.475045
C 2.414422 -2.665936 1.193781
C 2.936477 -2.018392 -0.082323
C 4.446399 -1.846918 -0.031693
H 2.799959 -2.622603 3.291306
H 2.843537 -3.674097 1.273867
H 1.329239 -2.787695 1.116855
H 2.661036 -2.625879 -0.950667
H 2.455843 -1.046812 -0.207706
H 4.794045 -1.278535 -0.901932
H 4.927711 -2.833206 -0.102709
C 4.143721 -1.243618 2.423167
C 4.905337 -1.180799 1.245962
C 6.136119 -0.516521 1.263572
C 6.631115 0.068186 2.420704
C 5.891309 -0.018464 3.597577
C 4.665897 -0.670096 3.588782
H 6.710647 -0.462791 0.341377
H 7.587883 0.581515 2.407912
H 6.267258 0.419893 4.517153
H 4.094194 -0.735347 4.511285
C -4.309237 2.871756 2.267529
C -3.656476 4.167152 1.775315
C -2.568514 3.941328 0.709451
C -4.937067 2.109775 1.107339
H -5.072549 3.106222 3.016933
H -3.575276 2.227931 2.758946
H -3.237989 4.730944 2.618162
H -4.441857 4.799355 1.342985
H -2.462951 4.885656 0.158163
H -5.260059 1.116062 1.432598
H -5.846824 2.634977 0.783495
C -4.025814 1.988388 -0.093360
C -2.978072 2.896030 -0.316723
C -2.309276 2.852281 -1.545163
C -2.601349 1.891936 -2.500885
C -3.580201 0.938445 -2.237832
C -4.291399 0.999596 -1.047325
H -1.535430 3.582284 -1.757212
H -2.060471 1.883208 -3.441510
H -3.809718 0.163671 -2.961039
H -5.078141 0.271405 -0.859071
C -1.207637 -2.954129 -1.084814
C -1.837176 -3.856568 -0.026068
C -3.096183 -3.210782 0.568769
C -2.189550 -2.617263 -2.216606
H -0.319181 -3.437060 -1.505097
H -0.853241 -2.035436 -0.608023
H -1.105182 -4.062531 0.760558
H -2.119678 -4.820970 -0.466926
H -3.550463 -3.910478 1.284594
H -1.943058 -1.631225 -2.630472
H -2.065155 -3.330797 -3.041277
C -3.657322 -2.631871 -1.830912
C -4.099232 -2.943619 -0.537454
C -5.473272 -2.986970 -0.278313
C -6.408212 -2.694646 -1.262694
C -5.970501 -2.374589 -2.546044
C -4.609539 -2.362337 -2.819899
H -5.809779 -3.257642 0.719596
H -7.469091 -2.728653 -1.035247
H -6.686152 -2.151185 -3.331181
H -4.266464 -2.139247 -3.828291
H -1.238039 0.377597 -0.395860

131
1-C$_{trans}$
Ru -0.472072 -0.100394 -
0.202387
C -0.630435 -2.025742 0.494154
C -0.010677 -4.242649 0.982984
C -1.465459 -4.040172 1.386517
H 0.075667 -4.850957 0.071796
H 0.580117 -4.722971 1.768486
H -2.124400 -4.816041 0.984991
H -1.588085 -4.024610 2.479024
C -0.233327 1.761485 -1.011217
C 0.923119 3.727484 -1.582830
C -0.541551 3.883729 -1.972198
H 1.169303 4.315334 -0.683895
H 1.601699 4.029952 -2.384290
H -0.976237 4.823441 -1.618732
H -0.673309 3.834151 -3.061866
N 0.989287 2.298799 -1.312013
N -1.141073 2.723730 -1.323764
N -1.748998 -2.730819 0.814145
N 0.412139 -2.870763 0.731854
C -2.582040 2.598343 -1.205508
H -2.743117 1.571078 -0.870531
C 2.204762 1.648657 -0.843213
H 1.981314 0.580641 -0.952529
C 1.800339 -2.562342 0.433917
H 1.805812 -1.511513 0.123102
C -3.102576 -2.212984 0.776281
H -2.984031 -1.175976 0.459545
C -3.939430 -2.959415 -0.305154
C -3.326828 -2.704920 -1.669040
C -5.415300 -2.543578 -0.300272
C -3.789756 -1.656597 -2.473652
C -5.547135 -1.075406 -0.696129
H -5.959075 -3.169309 -1.018940
C -5.017711 -0.842250 -2.113538
H -6.588095 -0.742245 -0.629026
H -5.809037 -1.094269 -2.832792
C 2.665880 -2.714034 1.725798
C 4.170226 -2.834572 1.452328
C 2.374996 -1.573653 2.692775
C 4.772814 -1.484410 1.077375
H 4.660186 -3.199655 2.364645
C 3.300122 -0.541451 2.913915
C 4.676538 -0.549408 2.278018
H 5.815812 -1.593668 0.767426
H 5.404740 -0.869014 3.036997
C 2.276570 -3.395419 -0.755320
H 2.368829 -4.458332 -0.503340
H 1.552346 -3.281589 -1.567649
C -3.725898 -2.227924 2.173528

H -3.967914 -3.242308 2.512255
H -3.027269 -1.777934 2.885480
H -4.648668 -1.642145 2.196570
C -2.240721 -3.466777 -2.105505
H -1.894326 -4.290796 -1.486800
C -3.129313 -1.378565 -3.672762
C -2.031309 -2.121189 -4.081800
H -1.523837 -1.877079 -5.009841
C -1.589383 -3.182016 -3.296490
H -0.737431 -3.778553 -3.607968
H -3.486730 -0.557880 -4.291179
C 1.145224 -1.526507 3.361527
C 2.956832 0.511656 3.768072
H 3.676881 1.311602 3.926168
C 1.722709 0.561359 4.399841
C 0.811674 -0.472195 4.198777
H 1.475046 1.396000 5.049429
H -0.157481 -0.456539 4.687140
C 3.398534 1.962915 -1.790682
C 4.196193 3.236810 -1.446839
C 4.304313 0.749412 -1.969058
C 5.359041 2.986638 -0.485912
H 4.605957 3.651273 -2.377020
C 5.668165 0.762980 -1.649509
C 6.344624 1.998154 -1.097445
H 5.866899 3.930486 -0.261688
H 7.101695 1.703597 -0.362143
C 2.447562 1.921622 0.640815
H 3.299960 1.344158 0.998156
H 2.638795 2.980893 0.845170
H 1.570853 1.613757 1.218996
C -3.245935 2.770280 -2.572880
H -2.755969 2.109136 -3.293435
H -4.304125 2.499564 -2.534526
H -3.181718 3.801443 -2.939735
C -3.110563 3.576875 -0.110904
C -2.597170 3.140308 1.248675
C -4.639158 3.692616 -0.085669
C -3.384196 2.337148 2.085665
C -5.263966 2.366991 0.338951
H -5.020111 4.008906 -1.061016
C -4.822988 1.986613 1.754546
H -6.356623 2.416334 0.293560
H -4.985404 0.915699 1.926961
C 3.752467 -0.410817 -2.531027
H 2.692686 -0.429356 -2.778515
C 6.437288 -0.382208 -1.886649
H 7.493214 -0.366065 -1.626430
C 4.530653 -1.531721 -2.781898
C 5.884343 -1.522239 -2.449733
C -1.306031 3.486039 1.658680
H -0.700748 4.114164 1.011044
C -2.846842 1.885424 3.294930
C -1.555039 2.215228 3.679830
H -1.155096 1.846494 4.619003
C -0.777861 3.025784 2.856441
H 0.236922 3.284879 3.141908
H -3.460588 1.262075 3.941671
H 6.502158 -2.395880 -2.633192
H 4.082318 -2.409053 -3.237513
H 4.361972 -3.583341 0.680152
H 2.350359 -3.649918 2.205165
H 4.240676 -1.057602 0.218224
H 4.964887 0.471483 1.999535
H -5.868514 -2.725936 0.678847
H -4.982215 -0.465554 0.016712
H -4.801783 0.221740 -2.266341
H -4.918868 4.477080 0.628829
H -4.961313 1.589775 -0.369645
H -5.465252 2.506943 2.478020
H 2.930981 2.121477 -2.770220
H 3.532514 4.003865 -1.036764
H 4.988167 2.593964 0.465321
H 6.888235 2.492223 -1.914969
H -3.888461 -4.035235 -0.088239
H -2.703313 4.572139 -0.333340
H 0.432450 -2.329290 3.207384
H 3.245382 -3.045364 -1.112532
H -0.855546 0.497384 1.387688
H -1.601950 0.513098 0.920538
H -1.825683 -0.259007 -0.933830
H 0.140842 -0.730862 -1.601028

131

1-C$_{cis}$

Ru -1.411058 0.346054 0.068829
C 0.259645 1.622476 0.546908
C 2.136878 2.588806 1.585270
C 1.313281 3.697965 0.931475
H 2.237844 2.726455 2.667199
H 3.138043 2.526305 1.145568
H 0.911421 4.414279 1.659185
H 1.903584 4.255718 0.199753
C -0.696289 -1.196107 -0.866436
C 0.109652 -2.457698 -2.670551
C -0.387974 -3.411347 -1.604084
H 1.066580 -2.761707 -3.094444
H -0.615322 -2.351727 -3.490434
H 0.450054 -3.908600 -1.096317
H -1.054217 -4.186035 -1.995505
N 0.199637 -1.216987 -1.911646
N -1.084666 -2.504925 -0.704897
N 0.238803 2.954649 0.282736
N 1.366268 1.384645 1.282007
C -1.930517 -3.046499 0.344168
H -2.094105 -2.216629 1.026524
C 0.974739 -0.083894 -2.392769
H 0.483959 0.780606 -1.948574
C 1.670352 0.099240 1.907366
H 1.036226 -0.591996 1.356762
C -0.847665 3.654596 -0.386791
H -1.257017 2.918532 -1.081222
C -1.996403 4.079381 0.580675
C -2.316014 3.024205 1.625813
C -3.268014 4.443006 -0.206007
C -3.406985 2.151008 1.487933
C -3.954980 3.196900 -0.770112
H -3.959514 4.961045 0.470881
C -4.407937 2.273001 0.356989
H -4.818805 3.487641 -1.376916
H -4.627668 1.277115 -0.038658
C 3.160484 -0.294054 1.725414
C 4.097494 0.283779 2.796554
C 3.375044 -1.807225 1.587964
C 5.540053 -0.111363 2.503619
H 4.005388 1.370857 2.841258
C 4.579250 -2.402994 2.008607
C 5.689959 -1.617456 2.676245
H 5.796532 0.180604 1.476551
H 6.656343 -1.959747 2.290136
C 1.195815 0.060745 3.359159
H 1.652522 0.847554 3.968644
H 1.448788 -0.905167 3.808700
C -0.308050 4.839879 -1.187269
H 0.070947 5.640269 -0.541864
H 0.497638 4.518583 -1.853461
H -1.094282 5.266058 -1.812748
C -1.554212 2.971615 2.804227
H -0.733167 3.673076 2.928885
C -3.672369 1.235441 2.519922
C -2.910996 1.193367 3.672813
H -3.142325 0.475112 4.452886
C -1.843712 2.079284 3.820778
H -1.241395 2.068999 4.723995
H -4.510546 0.554027 2.401985
C 2.422277 -2.625800 0.964169
C 4.779944 -3.772621 1.816061
H 5.717961 -4.212493 2.146755
C 3.819612 -4.571709 1.215537
C 2.633906 -3.987785 0.785538
H 3.995341 -5.634027 1.078614
H 1.867472 -4.595014 0.313870
C 2.418558 -0.161169 -1.828477
C 3.175325 -1.431575 -2.237003
C 3.240095 1.106197 -2.056081
C 4.623939 -1.410754 -1.761715
H 2.669966 -2.297876 -1.802958
C 4.626647 1.058213 -2.283384
C 5.369520 -0.253977 -2.413582
H 4.648508 -1.309439 -0.671130
H 5.511108 -0.472255 -3.481508
C 0.886876 0.069140 -3.908844
H -0.158067 0.192733 -4.207621
H 1.301647 -0.786558 -4.451112
H 1.442394 0.957713 -4.219911
C -1.180615 -4.141936 1.109121
H -0.197552 -3.774093 1.415296
H -1.718470 -4.422309 2.016782
H -1.043468 -5.050920 0.510939
C -3.310130 -3.541940 -0.196272
C -3.975580 -2.537046 -1.123698
C -4.258267 -3.901503 0.959302
C -5.008455 -1.697973 -0.685223
C -4.760335 -2.641989 1.667753
H -3.771458 -4.577298 1.667411
C -5.597069 -1.801279 0.705872
H -5.359997 -2.910366 2.543764
H -5.750761 -0.793276 1.108313
C 2.630105 2.364988 -1.970017
H 1.564846 2.429240 -1.780257
C 5.347147 2.250147 -2.410429
H 6.420126 2.194848 -2.579218
C 3.354590 3.542199 -2.108918
C 4.727652 3.488556 -2.328245
C -3.546754 -2.429341 -2.449701
H -2.784790 -3.112023 -2.813026

C -5.544064 -0.755299 -1.567298
C -5.071800 -0.623680 -2.865328
H -5.492022 0.128417 -3.525893
C -4.069194 -1.478466 -3.314445
H -3.701369 -1.408582 -4.333559
H -6.346091 -0.108313 -1.218149
H 5.307725 4.400148 -2.433212
H 2.846373 4.500931 -2.049269
H 3.815520 -0.096853 3.785986
H 3.478413 0.149151 0.770468
H 6.233194 0.414215 3.168084
H 5.688155 -1.855013 3.749027
H -3.036465 5.149967 -1.008252
H -3.274962 2.645655 -1.427702
H -5.340809 2.661335 0.790356
H -5.117986 -4.448595 0.551674
H -3.912950 -2.050228 2.032762
H -6.597858 -2.247150 0.618372
H 2.260563 -0.230028 -0.747108
H 3.162746 -1.548974 -3.329745
H 5.114616 -2.360765 -1.996464
H 6.373766 -0.148458 -1.988182
H -1.653147 4.974554 1.118757
H -3.119257 -4.455588 -0.778436
H 1.495958 -2.202135 0.589043
H 0.110890 0.184468 3.374487
H -1.375587 1.145301 -1.386691
H -2.686209 0.109804 -0.845329
H -2.619702 -0.549227 0.547370
H -1.136302 -0.267809 1.586492

133

1-C'$_{trans}$

Ru -0.007647 -0.029521 0.082233
C 0.474109 -1.364210 -1.450547
C 0.291411 -3.092433 -3.041172
C 1.719305 -2.564618 -3.048268
H 0.225918 -4.094132 -2.594951
H -0.150083 -3.140732 -4.040928
H 2.465000 -3.362099 -2.983379
H 1.934897 -1.970730 -3.946455
C -0.392052 1.201028 1.721152
C -1.485069 2.364448 3.452343
C 0.007591 2.343388 3.738725
H -1.823821 3.362747 3.145198
H -2.084653 2.048232 4.310793
H 0.408697 3.328968 3.994738
H 0.256079 1.655873 4.559248
N -1.586617 1.424446 2.339555
N 0.537710 1.844862 2.477909
N 1.722582 -1.716202 -1.863044
N -0.380921 -2.112852 -2.196339
C 1.949776 1.941400 2.172290
H 2.093648 1.226075 1.363769
C -2.896660 1.128315 1.777377
H -2.681060 0.605566 0.843761
C -1.821721 -1.966511 -2.230069
H -2.045578 -1.329235 -1.376984
C 2.962520 -1.136925 -1.383132
H 2.647726 -0.482900 -0.571591
C 3.904676 -2.217617 -0.776679
C 3.210971 -2.982178 0.341923
C 5.215803 -1.600031 -0.267170
C 3.440695 -2.674830 1.689716
C 4.975036 -0.764469 0.990487
H 5.914694 -2.411135 -0.025726
C 4.477707 -1.659452 2.122318
H 5.894380 -0.252746 1.294753
H 5.332225 -2.209687 2.541263
C -2.308448 -1.233008 -3.522700
C -3.778383 -0.801662 -3.394752
C -1.448270 -0.025055 -3.846742
C -3.929459 0.303837 -2.349435
H -4.123034 -0.437175 -4.370479
C -1.802173 1.256221 -3.409866
C -3.111359 1.545769 -2.708068
H -4.984521 0.573992 -2.232292
H -3.702676 2.196025 -3.367384
C -2.494308 -3.325612 -2.037414
H -2.320904 -3.994464 -2.888814
H -2.102774 -3.801451 -1.133882
C 3.612759 -0.299156 -2.488144
H 4.075895 -0.921858 -3.263371
H 2.847015 0.326162 -2.958583
H 4.384489 0.360823 -2.085616
C 2.307242 -4.003982 0.034173
H 2.150266 -4.275267 -1.005094
C 2.738432 -3.368899 2.678805
C 1.826616 -4.363514 2.358202
H 1.295181 -4.890588 3.145580
C 1.614002 -4.689282 1.021475
H 0.916849 -5.476440 0.749810
H 2.921797 -3.121129 3.722110
C -0.268158 -0.182076 -4.579793
C -0.939597 2.324365 -3.667848
H -1.219633 3.312036 -3.312653
C 0.258093 2.146684 -4.345455
C 0.591634 0.880403 -4.821676
H 0.916310 2.992281 -4.525297
H 1.506148 0.725378 -5.386693
C -3.685551 0.175686 2.724218
C -5.173210 0.058235 2.372032
C -3.029537 -1.190759 2.696314
C -5.357094 -0.613207 1.013919
H -5.667703 -0.540312 3.147546
C -3.494737 -2.185838 1.825098
C -4.764245 -2.024612 1.010036
H -6.417670 -0.659188 0.746119
H -4.576936 -2.348175 -0.020517
C -3.629472 2.426064 1.424278
H -4.487471 2.222661 0.778344
H -3.997976 2.960120 2.307916
H -2.945475 3.082398 0.876957
C 2.785504 1.472822 3.363583
H 2.441075 0.482806 3.677206
H 3.841037 1.389516 3.094121
H 2.713572 2.157003 4.217197
C 2.360476 3.366252 1.681049
C 1.342026 3.985584 0.737613
C 3.744701 3.331544 1.012109
C 1.517775 3.974138 -0.651601
C 3.675729 2.648670 -0.353883
H 4.471274 2.822934 1.652856
C 2.759343 3.409803 -1.309872
H 4.677488 2.560497 -0.787571
H 2.460796 2.761177 -2.142630
C -1.899283 -1.445184 3.478448
H -1.542566 -0.670788 4.151552
C -2.796430 -3.394404 1.742490
H -3.162219 -4.165999 1.068574
C -1.206328 -2.643890 3.380012
C -1.655294 -3.623740 2.498296
C 0.199190 4.601715 1.260265
H 0.083442 4.664125 2.338253
C 0.538049 4.547968 -1.467840
C -0.610360 5.117112 -0.938987
H -1.359773 5.547977 -1.596334
C -0.778240 5.151015 0.443676
H -1.656385 5.616140 0.881639
H 0.692123 4.543588 -2.543681
H -1.114107 -4.558532 2.397883
H -0.307575 -2.806219 3.965877
H -4.414424 -1.655168 -3.139569
H -2.226348 -1.942390 -4.358997
H -3.589374 -0.069390 -1.379180
H -2.899727 2.121914 -1.800332
H 5.692860 -1.004646 -1.050339
H 4.233107 0.015181 0.787562
H 4.074663 -1.053970 2.939882
H 4.105043 4.360245 0.886461
H 3.300173 1.628118 -0.234722
H 3.312658 4.247350 -1.756147
H -3.614446 0.579012 3.743131
H -5.650983 1.042433 2.384349
H -4.871933 -0.005165 0.245098
H -5.506312 -2.725731 1.415371
H 4.158700 -2.930334 -1.574248
H 2.421755 4.013878 2.567779
H -0.023591 -1.163305 -4.976991
H -3.575453 -3.219467 -1.920403
H 0.283965 1.300307 -0.914215
H 1.084769 1.086206 -0.575885
H 1.359303 -0.544840 0.899758
H -0.389136 -1.370350 1.060486
H -1.406936 0.473745 -0.671337
H -1.138992 -1.162455 0.657640

133

1-C'$_{cis}$

Ru -0.185585 -1.131076 -
1.025732
C 0.026845 0.921194 -0.814018
C -0.443875 3.181550 -1.202612
C 0.971191 3.072275 -0.682370
H -0.458676 3.325962 -2.292433
H -1.012473 3.988197 -0.742191
H 1.687825 3.626864 -1.288763
H 1.051940 3.422992 0.356999
C -1.411105 -1.423444 0.720115
C -2.153053 -1.582412 2.961054
C -3.366933 -1.579963 2.038411
H -2.209223 -0.803657 3.726660
H -2.029568 -2.546042 3.467555
H -3.965051 -0.666200 2.142553
H -4.032016 -2.431518 2.215218
N -1.049624 -1.339324 2.030046

N -2.755301 -1.642074 0.716801
N 1.189773 1.629046 -0.754474
N -0.972050 1.863092 -0.856940
C -3.545115 -2.064099 -0.426608
H -2.921015 -1.816189 -1.285550
C 0.336900 -1.353511 2.500766
H 0.886105 -1.856053 1.706327
C -2.374293 1.565229 -1.118005
H -2.484282 0.526205 -0.811519
C 2.541132 1.078183 -0.649626
H 2.436054 0.143005 -0.105054
C 3.117224 0.756473 -2.054838
C 4.461097 0.043825 -1.962944
C 3.140075 1.990774 -2.967816
C 5.571742 0.437319 -2.725057
C 4.033038 1.780722 -4.185567
H 2.113446 2.221059 -3.271424
C 5.471721 1.573961 -3.719163
H 3.695986 0.901316 -4.748379
H 5.836042 2.503340 -3.258743
C -3.375849 2.355976 -0.221069
C -3.102803 2.108013 1.272966
C -3.573827 3.836416 -0.530391
C -2.111535 3.099947 1.877574
H -4.050232 2.176026 1.824179
C -3.163637 4.848246 0.351590
C -2.594057 4.536900 1.718333
H -1.973514 2.887704 2.941957
H -3.383335 4.731927 2.457760
C -2.672734 1.615573 -2.616455
H -2.544569 2.614438 -3.041838
H -3.701431 1.293980 -2.812239
C 3.449758 1.968881 0.197190
H 3.682915 2.925500 -0.278969
H 2.997881 2.167071 1.172102
H 4.390423 1.444206 0.376480
C 4.591351 -1.074668 -1.129119
H 3.731432 -1.421667 -0.565561
C 6.777443 -0.258350 -2.595526
C 6.901528 -1.342936 -1.738520
H 7.847145 -1.869865 -1.656231
C 5.791955 -1.761602 -1.009641
H 5.857105 -2.627985 -0.357605
H 7.629708 0.056356 -3.193454
C -4.221015 4.213106 -1.714909
C -3.341544 6.188064 -0.008649
H -3.008286 6.962233 0.678803
C -3.941585 6.544846 -1.207575
C -4.399833 5.545216 -2.061334
H -4.066695 7.591679 -1.466221
H -4.900066 5.801142 -2.990107
C 0.897947 0.079240 2.629434
C 0.086567 0.936491 3.601464
C 2.394217 0.118067 2.925491
C 0.727651 2.307223 3.779618
H -0.931075 1.029959 3.213730
C 2.952070 1.112363 3.746590
C 2.091913 2.147299 4.442026
H 0.843495 2.783025 2.796359
H 1.947118 1.848172 5.489551
C 0.506544 -2.199421 3.762278
H 0.180455 -3.227202 3.579614
H -0.042916 -1.808003 4.623608
H 1.565141 -2.223489 4.029992
C -4.843929 -1.257270 -0.501584
H -4.635955 -0.196198 -0.345604
H -5.314529 -1.359880 -1.481335
H -5.571127 -1.576875 0.254261
C -3.823770 -3.602095 -0.432313
C -2.584785 -4.428363 -0.132496
C -4.429172 -4.038214 -1.776517
C -1.822098 -5.004362 -1.157018
C -3.385846 -3.962382 -2.892229
H -5.305546 -3.432738 -2.023865
C -2.228952 -4.923289 -2.614281
H -3.842466 -4.207230 -3.856401
H -1.357582 -4.646696 -3.218390
C 3.262006 -0.788175 2.302087
H 2.858386 -1.555952 1.649467
C 4.338388 1.166413 3.916476
H 4.757239 1.947170 4.547131
C 4.638318 -0.723486 2.473811
C 5.184133 0.262108 3.289799
C -2.182550 -4.623730 1.193023
H -2.802677 -4.230951 1.993618
C -0.657949 -5.706731 -0.827160
C -0.244401 -5.851402 0.489133
H 0.668064 -6.393206 0.717702
C -1.023137 -5.315172 1.512359
H -0.735758 -5.448549 2.551177
H -0.064668 -6.140576 -1.628579
H 6.258540 0.331386 3.427212
H 5.280810 -1.426629 1.954125
H -2.722706 1.088550 1.399007
H -4.337152 1.880130 -0.456984
H -1.135451 2.964278 1.403451
H -1.784545 5.238159 1.951334
H 3.521044 2.866750 -2.425295
H 3.971499 2.641182 -4.859970
H 6.132688 1.371229 -4.568820
H -4.783846 -5.072279 -1.681630
H -3.000196 -2.940862 -2.980433
H -2.514102 -5.934805 -2.934377
H 0.763763 0.519626 1.631493
H 0.019090 0.449288 4.584023
H 0.089251 2.965437 4.378472
H 2.624927 3.103927 4.468267
H 2.407191 0.044983 -2.494034
H -4.559691 -3.805769 0.359033
H -4.602280 3.441042 -2.375155
H -1.996324 0.920663 -3.120400
H 1.197992 -1.570074 -0.066948
H -0.463954 -2.838301 -1.140661
H -0.213565 -2.578579 -1.926627
H -1.526536 -0.835762 -1.859723
H 0.405558 -0.779176 -2.508452
H 1.464749 -1.445976 -0.922174

129

1-C″$_{trans}$

Ru -0.679577 -0.940671 -
0.156224
C -2.359687 0.133938 -0.619999
C -3.672154 2.078327 -0.850444
C -4.400657 0.899903 -1.485318
H -4.142103 2.376199 0.098129
H -3.635789 2.951567 -1.506081
H -5.448179 0.838240 -1.188518
H -4.347740 0.945815 -2.582598
C 1.050028 -1.889427 0.322012
C 2.564721 -2.838063 1.879573
C 3.108992 -3.034942 0.458570
H 3.289516 -2.334765 2.526323
H 2.271274 -3.784305 2.354970
H 4.038861 -2.480689 0.305862
H 3.305340 -4.085897 0.232174
N 1.400715 -2.001147 1.634423
N 2.034266 -2.495149 -0.388901
N -3.615718 -0.230018 -0.985802
N -2.352265 1.499376 -0.620009
C 1.881406 -2.824274 -1.802518
H 0.885967 -2.444215 -2.044147
C 0.670488 -1.304653 2.675169
H 1.056824 -1.677861 3.630764
C -1.179781 2.263177 -0.218212
H -0.632577 1.602562 0.467910
C -4.032629 -1.611450 -1.156381
H -3.116740 -2.188473 -1.004579
C -5.029060 -2.096890 -0.057789
C -6.309378 -1.281923 0.008859
C -4.347416 -2.205837 1.318596
C -6.544348 -0.357075 1.038786
C -4.264981 -0.872858 2.064573
H -4.918855 -2.913586 1.933846
C -5.651957 -0.268040 2.259198
H -3.794454 -1.026895 3.042194
H -6.163846 -0.798894 3.074366
C -0.260042 2.540566 -1.436476
C -1.006630 3.227486 -2.583984
C 1.046302 3.242611 -1.079199
C -0.052105 3.565707 -3.724148
H -1.798330 2.559031 -2.935838
C 1.644491 4.166175 -1.952451
C 0.979053 4.583270 -3.247158
H 0.454166 2.649508 -4.054941
H 1.744576 4.747747 -4.013245
C -1.563582 3.506758 0.581166
H -2.140722 4.219068 -0.016041
H -0.661661 4.017290 0.925199
C -4.495522 -1.860593 -2.591839
H -5.357845 -1.248796 -2.872364
H -3.674663 -1.630312 -3.277455
H -4.769807 -2.911082 -2.727498
C -7.271897 -1.418788 -0.999894
H -7.126066 -2.175663 -1.765002
C -7.681441 0.455301 0.978406
C -8.599209 0.347537 -0.057745
H -9.471426 0.993423 -0.083808
C -8.402400 -0.614073 -1.045634
H -9.126355 -0.738067 -1.845033
H -7.849233 1.179853 1.772133
C 1.736327 2.901668 0.090854
C 2.890176 4.712104 -1.627686
H 3.345210 5.418946 -2.317802
C 3.555583 4.367661 -0.459203
C 2.970755 3.451557 0.409709

H 4.524363 4.802287 -0.233122
H 3.473088 3.147749 1.323132
C 0.951996 0.224731 2.568508
C 0.052604 1.097168 3.445395
C 2.415590 0.532489 2.818632
C 0.440107 0.954329 4.916191
H 0.178024 2.144299 3.137405
C 2.844330 1.057458 4.045941
C 1.867786 1.459000 5.135588
H -0.250690 1.510683 5.557283
H 2.246219 1.127740 6.109013
C -0.821439 -1.635302 2.587596
H -1.345771 -1.336540 3.500325
H -1.363076 -1.089449 1.777478
H -0.964975 -2.706291 2.439482
C 1.845242 -4.342952 -1.998283
H 1.090517 -4.779262 -1.337191
H 1.574081 -4.580987 -3.030866
H 2.805436 -4.824089 -1.788361
C 2.881055 -2.113355 -2.775095
C 4.302753 -2.050827 -2.246553
C 2.390238 -0.724199 -3.210116
C 4.841415 -0.860868 -1.729526
C 2.626345 0.336702 -2.141118
H 1.325130 -0.773482 -3.458629
C 4.111689 0.463968 -1.819377
H 2.253116 1.306375 -2.481420
H 4.252039 1.038196 -0.896390
C 3.363937 0.275407 1.819947
H 3.029598 -0.092527 0.854684
C 4.210972 1.275842 4.250899
H 4.540239 1.683589 5.203953
C 4.718164 0.483073 2.039702
C 5.146400 0.983382 3.267640
C 5.092631 -3.206693 -2.241908
H 4.697512 -4.114018 -2.690554
C 6.126457 -0.886276 -1.176752
C 6.879491 -2.052584 -1.128507
H 7.870517 -2.044127 -0.685191
C 6.362506 -3.221695 -1.680328
H 6.947951 -4.135961 -1.680528
H 6.546086 0.040732 -0.791051
H 6.202618 1.151658 3.454222
H 5.430171 0.252184 1.254055
H -1.487259 4.153527 -2.237195
H 0.012820 1.538405 -1.798730
H -0.600598 3.959159 -4.586147
H 0.482131 5.552220 -3.097397
H -3.343313 -2.623721 1.188622
H -3.627935 -0.179095 1.512547
H -5.573667 0.775982 2.583609
H 2.926598 -0.436393 -4.124924
H 2.048541 0.079752 -1.247214
H 4.598959 1.052016 -2.609779
H 0.735708 0.448488 1.514969
H -1.001087 0.852504 3.282682
H 0.362517 -0.098637 5.215310
H 1.849796 2.556673 5.176484
H -5.300724 -3.115567 -0.365702
H 2.895235 -2.744555 -3.673034
H 1.312439 2.175681 0.772581
H -2.160213 3.230794 1.455588
H -1.216230 -2.339535 -0.592655
H -0.301145 -0.730039 -1.667599

129

1-C″$_{cis}$

Ru 0.792186 -0.980910 -0.768131
C 0.329166 -2.136908 0.862897
C -0.243480 -3.297768 2.822906
C 0.127880 -4.317870 1.737029
H 0.507969 -3.264652 3.622333
H -1.213083 -3.513648 3.277940
H 0.874771 -5.041000 2.082799
H -0.749797 -4.878696 1.386771
C 0.245240 0.817061 -0.486582
C -0.709287 2.963573 -0.587327
C 0.551295 3.039898 0.280711
H -0.576654 3.510562 -1.530330
H -1.576716 3.377022 -0.066852
H 1.249099 3.802585 -0.069827
H 0.312445 3.269997 1.328307
N -0.869778 1.534141 -0.833141
N 1.093883 1.699424 0.138750
N 0.648070 -3.447865 0.694329
N -0.252733 -2.038995 2.076134
C 2.379475 1.282006 0.665703
H 2.428392 0.190314 0.507284
C -1.856393 1.023854 -1.780427
H -1.568209 -0.019495 -1.892570
C -0.819990 -0.792817 2.568106
H -0.196846 -0.019513 2.114717
C 1.220347 -3.906679 -0.556561
H 1.174837 -3.050142 -1.256676
C 2.708705 -4.321055 -0.391580
C 3.513939 -3.205954 0.250425
C 3.348176 -4.738023 -1.723958
C 4.294782 -2.336897 -0.525059
C 3.514933 -3.532002 -2.649431
H 4.334103 -5.171392 -1.513996
C 4.456075 -2.501961 -2.023164
H 3.912371 -3.850157 -3.618172
H 5.496083 -2.802487 -2.210219
C -2.251830 -0.599553 2.010243
C -3.192517 -1.763642 2.339200
C -2.852322 0.759335 2.349416
C -4.618529 -1.473324 1.879989
H -2.819171 -2.666909 1.845335
C -4.228207 0.922820 2.577220
C -5.170940 -0.259916 2.619268
H -4.622827 -1.284385 0.801041
H -6.141567 0.032941 2.204883
C -0.702085 -0.681790 4.085227
H -1.283733 -1.448520 4.606330
H -1.071142 0.292119 4.413731
C 0.344726 -4.989826 -1.187038
H 0.413563 -5.933052 -0.633389
H -0.696159 -4.655435 -1.191595
H 0.636632 -5.180919 -2.221030
C 3.478905 -3.031863 1.638773
H 2.890044 -3.719759 2.239234
C 5.011849 -1.320132 0.115799
C 4.957484 -1.148085 1.491347
H 5.525372 -0.350772 1.962380
C 4.181691 -2.011573 2.262228
H 4.136829 -1.897015 3.340829
H 5.622866 -0.653185 -0.487674
C -2.041628 1.900433 2.342389
C -4.742818 2.208088 2.776475
H -5.811654 2.322115 2.941263
C -3.925834 3.330147 2.764665
C -2.559868 3.171222 2.549815
H -4.349917 4.317492 2.918163
H -1.898800 4.033314 2.536299
C -3.293821 1.054097 -1.191971
C -4.019685 2.412877 -1.275544
C -4.131464 -0.099288 -1.737795
C -4.916196 2.554095 -2.504307
H -4.636956 2.531918 -0.375819
C -5.385010 0.086671 -2.336325
C -5.983245 1.466322 -2.495236
H -5.386575 3.543159 -2.514074
H -6.584801 1.502193 -3.410217
C -1.707216 1.658109 -3.162052
H -2.370784 1.152296 -3.869612
H -1.952284 2.725454 -3.176718
H -0.676825 1.527619 -3.505793
C 2.430794 1.485780 2.181691
H 1.629324 0.910214 2.653125
H 3.382770 1.131878 2.587220
H 2.308572 2.536568 2.458046
C 3.615073 1.851825 -0.099615
C 3.802014 3.359517 0.005681
C 3.650433 1.380040 -1.561140
C 3.575350 4.213367 -1.086534
C 2.787768 2.232517 -2.487743
H 3.313856 0.339853 -1.619071
C 3.246397 3.687264 -2.468659
H 2.840521 1.836708 -3.506541
H 2.496579 4.334780 -2.937966
C -3.647940 -1.408859 -1.592503
H -2.680267 -1.571260 -1.123200
C -6.108099 -1.027037 -2.778280
H -7.075972 -0.868581 -3.248582
C -4.375645 -2.506440 -2.029262
C -5.617891 -2.316017 -2.630322
C 4.231407 3.922361 1.214528
H 4.470852 3.264724 2.044869
C 3.712336 5.594408 -0.911014
C 4.092272 6.141124 0.307032
H 4.188463 7.216965 0.416452
C 4.370229 5.294264 1.376295
H 4.699419 5.698871 2.328418
H 3.523290 6.248838 -1.758931
H -6.196623 -3.164558 -2.982409
H -3.973312 -3.506957 -1.901030
H -3.199120 -1.959618 3.421132
H -2.103365 -0.611013 0.920883
H -5.260561 -2.343086 2.053149
H -5.352745 -0.526555 3.670207
H 2.762001 -5.524908 -2.206451
H 2.544834 -3.063945 -2.851918
H 4.322871 -1.529759 -2.509210
H 4.691452 1.414192 -1.912327
H 1.741897 2.150259 -2.186043
H 4.154607 3.787395 -3.079722

H -3.153597 0.848568 -0.128537
H -3.293836 3.229830 -1.252909
H -4.321195 2.473608 -3.420539
H -6.676647 1.652792 -1.662719
H 2.718902 -5.185438 0.287673
H 4.467757 1.392519 0.414413
H -0.979731 1.793153 2.153431
H 0.342950 -0.776233 4.395794
H -0.532177 -1.470681 -1.419373
H 1.214249 -0.482750 -2.310885

149

1-C$_{trans}$-bf(si)

Ru 0.535465 0.094636 0.079835
C 0.853276 1.866582 -0.956621
C 0.478606 3.750345 -2.310009
C 1.883395 3.825115 -1.747164
H 0.482986 3.487425 -3.377233
H -0.075205 4.682035 -2.186405
H 2.628181 4.087321 -2.502354
H 1.948131 4.559612 -0.930942
C 0.056661 -1.631719 1.216556
C -1.115502 -3.539298 1.977483
C 0.223916 -3.473908 2.699600
H -1.970420 -3.513737 2.662780
H -1.195291 -4.445623 1.369332
H 0.116238 -3.399411 3.789614
H 0.842609 -4.348940 2.480529
N -1.087166 -2.359746 1.123047
N 0.827860 -2.267204 2.145351
N 2.062353 2.467828 -1.239195
N -0.089742 2.661252 -1.521071
C 2.089248 -1.818235 2.711689
H 2.521917 -1.141586 1.979113
C -2.312531 -1.917398 0.469636
H -1.970139 -1.175431 -0.243802
C -1.537969 2.453275 -1.456785
H -1.664913 1.373981 -1.396800
C 3.299885 2.147329 -0.538959
H 3.192394 1.106545 -0.241081
C 4.541173 2.228284 -1.474401
C 4.458511 1.147575 -2.539180
C 5.853956 2.099655 -0.688306
C 5.132412 -0.074299 -2.383796
C 6.007987 0.693183 -0.110272
H 6.688700 2.296125 -1.372688
C 6.101832 -0.325821 -1.245395
H 6.901027 0.630954 0.519428
H 7.118990 -0.298403 -1.659881
C -2.097699 3.053507 -0.143287
C -1.718844 4.526319 0.044259
C -3.590008 2.802087 0.035337
C -2.405424 5.115635 1.270969
H -0.630930 4.602196 0.145048
C -4.435018 3.762345 0.615911
C -3.916065 5.113802 1.061543
H -2.149108 4.510810 2.150048
H -4.434930 5.419296 1.976954
C -2.234394 2.930781 -2.729248
H -2.204635 4.017305 -2.858083
H -3.283919 2.631843 -2.693414
C 3.427229 2.999000 0.729279
H 3.735031 4.027608 0.510985
H 2.458628 3.025543 1.238047
H 4.157680 2.571837 1.421176
C 3.636466 1.327093 -3.655729
H 3.123289 2.275116 -3.786400
C 4.928297 -1.088774 -3.323580
C 4.084649 -0.909065 -4.410785
H 3.928539 -1.717328 -5.117904
C 3.444610 0.314626 -4.586039
H 2.788358 0.474464 -5.435693
H 5.432544 -2.041900 -3.185414
C -4.141304 1.567587 -0.331151
C -5.790556 3.470150 0.791783
H -6.432619 4.221770 1.245968
C -6.326230 2.249770 0.404285
C -5.491172 1.290123 -0.159104
H -7.383163 2.046356 0.547807
H -5.878829 0.325400 -0.470320
C -3.029116 -3.017905 -0.365017
C -3.820646 -4.054229 0.471095
C -3.881322 -2.299293 -1.410096
C -5.144277 -4.484511 -0.159037
H -3.199044 -4.936431 0.650475
C -5.280058 -2.352796 -1.421476
C -6.001647 -3.255028 -0.450363
H -4.963703 -5.026461 -1.095545
H -6.201204 -2.722763 0.490856
C -3.220559 -1.196155 1.466458
H -4.198011 -0.983914 1.024622
H -3.380565 -1.771024 2.385472
H -2.752009 -0.246269 1.734789
C 3.055635 -2.997900 2.879670
H 2.995372 -3.657200 2.013739
H 4.086409 -2.643659 2.949274
H 2.839934 -3.577825 3.784546
C 1.889973 -1.074690 4.076877
C 0.872113 0.055105 4.027294
C 3.225467 -0.563297 4.639953
C 1.264131 1.402138 3.990740
C 3.699247 0.677520 3.883221
H 3.983547 -1.349440 4.623899
C 2.715271 1.819244 4.120004
H 4.701175 0.972361 4.211840
H 2.922230 2.659825 3.451015
C -3.228843 -1.533154 -2.382852
H -2.144369 -1.537070 -2.400011
C -5.988156 -1.610734 -2.372334
H -7.074432 -1.663132 -2.376958
C -3.938300 -0.787748 -3.314175
C -5.331266 -0.814931 -3.301832
C -0.494116 -0.242432 4.037694
H -0.809837 -1.280360 4.097984
C 0.286547 2.397512 3.903147
C -1.065105 2.084603 3.885818
H -1.809830 2.871999 3.821771
C -1.458052 0.752823 3.971684
H -2.510970 0.491062 3.979295
H 0.600052 3.438520 3.861679
H -5.900248 -0.236245 -4.022701
H -3.405106 -0.193481 -4.050708
H -2.009314 5.117831 -0.836422
H -1.584726 2.489251 0.648336
H -2.051222 6.133639 1.464925
H -4.170581 5.865314 0.300694
H 5.911830 2.858446 0.096297
H 5.153940 0.456863 0.537427
H 5.961085 -1.342908 -0.863182
H 3.081270 -0.295933 5.694454
H 3.772990 0.460051 2.809539
H 2.865462 2.201791 5.139442
H -2.234630 -3.534274 -0.915794
H -4.050941 -3.635892 1.457592
H -5.667380 -5.169915 0.516249
H -6.975230 -3.540391 -0.862437
H 4.541484 3.210171 -1.964821
H 1.503430 -1.830919 4.774392
H -3.510087 0.796173 -0.760542
H -1.780820 2.466638 -3.610621
H -0.392303 0.900181 1.129736
H 1.481162 0.808754 1.383695
H 2.045426 0.302827 1.043650
C 0.601370 -0.973949 -1.819093
C 1.922188 -1.065333 -1.290763
C 2.125068 -2.478178 -0.984152
C 3.201117 -3.233800 -0.539398
C 3.032839 -4.607727 -0.343522
C 1.797074 -5.215181 -0.571744
C 0.708536 -4.475608 -1.043253
C 0.913410 -3.125779 -1.271784
H 4.155568 -2.758607 -0.329024
H 3.869077 -5.207891 0.002742
H 1.683420 -6.282062 -0.404995
H -0.247496 -4.941338 -1.259380
O 0.006828 -2.271022 -1.799336
H 2.725221 -0.448882 -1.666730
C 0.223194 -0.245399 -3.081839
H 0.390465 -0.899630 -3.945269
H -0.823304 0.071245 -3.078632
H 0.851194 0.638454 -3.197101
H -0.920373 0.067592 -0.509611

149

TS$^{HT}_{trans}$(si)

Ru 0.510313 0.089287 0.065591
C 0.829233 1.819134 -1.032211
C 0.450573 3.653925 -2.444370
C 1.864401 3.736701 -1.898639
H 0.441243 3.342839 -3.498940
H -0.092784 4.595520 -2.358225
H 2.603542 3.967079 -2.669554
H 1.942555 4.497270 -1.107835
C 0.102748 -1.562613 1.302432
C -0.944526 -3.507309 2.128963
C 0.319235 -3.256560 2.939924
H -1.833916 -3.617685 2.755292
H -0.844206 -4.405082 1.508360
H 0.104301 -3.015143 3.990565
H 0.990734 -4.118263 2.922294
N -1.023156 -2.324939 1.279907
N 0.911971 -2.115686 2.250143
N 2.037295 2.395178 -1.346852
N -0.115907 2.604659 -1.602086
C 2.176041 -1.590444 2.743317
H 2.533038 -0.917365 1.964822

C -2.286273 -1.919382 0.677119
H -2.002941 -1.105369 0.015643
C -1.558029 2.400411 -1.479207
H -1.670663 1.332421 -1.300570
C 3.275204 2.064684 -0.650920
H 3.131881 1.037712 -0.313606
C 4.505187 2.088118 -1.603133
C 4.381233 0.990627 -2.646635
C 5.824455 1.937812 -0.831764
C 5.027138 -0.244614 -2.478633
C 5.944879 0.538858 -0.227923
H 6.655318 2.095964 -1.530757
C 6.001307 -0.501730 -1.345676
H 6.841546 0.463440 0.395226
H 7.014417 -0.506952 -1.770965
C -2.096235 3.130601 -0.220176
C -1.776900 4.630406 -0.215604
C -3.571604 2.836718 0.019965
C -2.537419 5.358174 0.887656
H -0.697130 4.763697 -0.088296
C -4.481985 3.835117 0.399835
C -4.035905 5.265575 0.616261
H -2.306563 4.896078 1.856173
H -4.608053 5.704509 1.441071
C -2.288165 2.740307 -2.775864
H -2.239763 3.803309 -3.032949
H -3.342042 2.472133 -2.673102
C 3.439524 2.953333 0.586488
H 3.748759 3.972999 0.330158
H 2.486338 2.998736 1.122196
H 4.185878 2.540474 1.269587
C 3.555349 1.173435 -3.759961
H 3.065047 2.131984 -3.901437
C 4.792204 -1.267272 -3.402209
C 3.946683 -1.082658 -4.487142
H 3.768438 -1.896362 -5.182813
C 3.334432 0.153356 -4.675461
H 2.678655 0.317859 -5.524755
H 5.274795 -2.229946 -3.253539
C -4.038940 1.523342 -0.105958
C -5.824120 3.499626 0.601026
H -6.519771 4.281294 0.898464
C -6.280388 2.199237 0.431660
C -5.376039 1.201141 0.081099
H -7.329649 1.964596 0.583375
H -5.703174 0.175190 -0.059106
C -2.953776 -3.010273 -0.208611
C -3.586400 -4.177153 0.585635
C -3.934116 -2.316587 -1.153287
C -4.849782 -4.747755 -0.054722
H -2.847819 -4.973360 0.716723
C -5.313393 -2.562895 -1.140287
C -5.877203 -3.633278 -0.236985
H -4.618074 -5.189295 -1.031877
H -6.129075 -3.211606 0.746582
C -3.215218 -1.325565 1.738149
H -4.206340 -1.127263 1.320957
H -3.339871 -1.979159 2.608012
H -2.791049 -0.378027 2.077561
C 3.198936 -2.722938 2.902805
H 3.098035 -3.430596 2.079726
H 4.217718 -2.328819 2.878012
H 3.072376 -3.260604 3.849935
C 2.000243 -0.807786 4.090579
C 1.032189 0.363079 3.995564
C 3.350103 -0.334219 4.651582
C 1.488739 1.688635 3.907175
C 3.886275 0.852286 3.851559
H 4.071518 -1.153419 4.679624
C 2.954954 2.044496 4.045505
H 4.900413 1.111634 4.172352
H 3.207061 2.857809 3.358483
C -3.428479 -1.396061 -2.077565
H -2.356332 -1.237149 -2.114488
C -6.146308 -1.858435 -2.015288
H -7.214495 -2.061628 -2.000623
C -4.260649 -0.695847 -2.939292
C -5.634565 -0.918842 -2.900757
C -0.346460 0.130941 3.987655
H -0.714237 -0.887225 4.078532
C 0.562081 2.722546 3.746119
C -0.802778 2.472408 3.708388
H -1.506736 3.289303 3.582385
C -1.259856 1.166907 3.850129
H -2.324341 0.956907 3.842419
H 0.926529 3.744281 3.662920
H -6.299542 -0.376404 -3.565328
H -3.837573 0.020116 -3.637809
H -2.051095 5.088470 -1.176894
H -1.540250 2.675698 0.611738
H -2.225558 6.406251 0.946988
H -4.274323 5.863269 -0.275022
H 5.915131 2.710341 -0.064037
H 5.089687 0.337558 0.429834
H 5.839010 -1.508480 -0.945528
H 3.203738 -0.017235 5.692075
H 3.948546 0.591688 2.786461
H 3.110024 2.445361 5.057246
H -2.150865 -3.406074 -0.839118
H -3.858783 -3.839411 1.592495
H -5.254455 -5.547277 0.574845
H -6.809890 -4.020373 -0.660399
H 4.526868 3.059797 -2.113862
H 1.581350 -1.528336 4.806494
H -3.345443 0.730460 -0.360915
H -1.868559 2.168780 -3.610038
H -0.727833 0.811622 0.818499
H 1.061622 0.935719 1.407185
H 1.800533 0.528590 1.081261
C 0.477940 -1.090031 -1.874704
C 1.807832 -1.141651 -1.334136
C 2.017038 -2.538855 -0.971217
C 3.088460 -3.268686 -0.474555
C 2.923619 -4.633603 -0.216068
C 1.699725 -5.262651 -0.440044
C 0.615889 -4.550929 -0.967678
C 0.817366 -3.212445 -1.243135
H 4.035773 -2.778278 -0.268538
H 3.757978 -5.209462 0.173820
H 1.589715 -6.321556 -0.227535
H -0.332534 -5.032096 -1.184167
O -0.095019 -2.384457 -1.824813
H 2.595068 -0.550233 -1.778022
C 0.152487 -0.404202 -3.179855
H 0.474014 -1.048825 -4.003458
H -0.916094 -0.198833 -3.287638
H 0.699415 0.536419 -3.243911
H -0.651000 -0.351458 -0.987505

149

int$^1_{trans}$(si)

Ru 0.507138 0.135299 0.111883
C 0.905786 1.782495 -1.091544
C 0.597207 3.545167 -2.610958
C 2.003864 3.615452 -2.042880
H 0.596585 3.185741 -3.649827
H 0.078137 4.503959 -2.577141
H 2.762951 3.783995 -2.809922
H 2.091411 4.413295 -1.290922
C 0.125691 -1.461792 1.406879
C -0.878301 -3.419208 2.254924
C 0.365740 -3.108364 3.080417
H -1.776328 -3.523168 2.870352
H -0.748305 -4.337670 1.671983
H 0.129649 -2.869892 4.126626
H 1.073581 -3.941055 3.074746
N -0.967560 -2.272776 1.357253
N 0.920745 -1.948156 2.392579
N 2.128715 2.301602 -1.416277
N -0.010768 2.551539 -1.729699
C 2.145753 -1.349076 2.900373
H 2.466096 -0.663259 2.117293
C -2.232727 -1.903232 0.730376
H -1.961787 -1.123725 0.022901
C -1.457930 2.374366 -1.631065
H -1.601103 1.316207 -1.417335
C 3.353378 1.953953 -0.704324
H 3.167472 0.948923 -0.327276
C 4.582826 1.894412 -1.652959
C 4.407269 0.772066 -2.662552
C 5.889290 1.702826 -0.868779
C 4.978066 -0.491835 -2.444146
C 5.937920 0.319093 -0.220490
H 6.730837 1.798016 -1.566476
C 5.943016 -0.757049 -1.305350
H 6.830375 0.218704 0.405330
H 6.952930 -0.817214 -1.734386
C -2.011893 3.163613 -0.415315
C -1.697282 4.662760 -0.488311
C -3.487855 2.874785 -0.169466
C -2.466818 5.443713 0.571436
H -0.618969 4.805860 -0.360516
C -4.403090 3.887794 0.156443
C -3.963631 5.329592 0.298786
H -2.237333 5.035102 1.563959
H -4.541285 5.808756 1.096832
C -2.156584 2.666803 -2.956352
H -2.070489 3.712376 -3.267585
H -3.219869 2.436709 -2.856469
C 3.534683 2.880730 0.500850
H 3.859538 3.887038 0.211822
H 2.578636 2.949105 1.028243
H 4.271295 2.479813 1.201527
C 3.609803 0.964496 -3.794662
H 3.183355 1.945663 -3.980681
C 4.689517 -1.532289 -3.331662

C 3.872838 -1.335669 -4.436569
H 3.655279 -2.161623 -5.106341
C 3.342907 -0.071401 -4.679668
H 2.714254 0.103276 -5.547403
H 5.110585 -2.516177 -3.141483
C -3.949958 1.554674 -0.227615
C -5.743911 3.557091 0.373338
H -6.443376 4.350108 0.628584
C -6.194285 2.247491 0.272737
C -5.285608 1.236237 -0.024380
H -7.242513 2.016503 0.436341
H -5.608356 0.202894 -0.110253
C -2.904630 -3.036299 -0.097861
C -3.505244 -4.175038 0.758876
C -3.917522 -2.391708 -1.045410
C -4.766403 -4.795681 0.163323
H -2.749656 -4.950074 0.915148
C -5.292225 -2.660982 -0.991846
C -5.818589 -3.708907 -0.040886
H -4.542401 -5.269379 -0.800435
H -6.062372 -3.256746 0.931059
C -3.164676 -1.254924 1.756866
H -4.156570 -1.081914 1.330548
H -3.284535 -1.864729 2.658392
H -2.742563 -0.291210 2.048784
C 3.224903 -2.420045 3.096321
H 3.186752 -3.137974 2.276085
H 4.219719 -1.967911 3.098133
H 3.102913 -2.960682 4.042560
C 1.895596 -0.542837 4.217325
C 0.895970 0.587757 4.033454
C 3.206899 0.002419 4.802944
C 1.317846 1.915352 3.862226
C 3.744663 1.149317 3.947993
H 3.947168 -0.793926 4.909707
C 2.770615 2.322780 4.003983
H 4.731792 1.466261 4.300199
H 3.022043 3.074076 3.249291
C -3.454034 -1.496163 -2.015633
H -2.387644 -1.320435 -2.094302
C -6.158416 -2.003231 -1.870897
H -7.221745 -2.225692 -1.822941
C -4.318717 -0.841880 -2.881568
C -5.686597 -1.087506 -2.802182
C -0.474838 0.314125 4.025019
H -0.812028 -0.707662 4.179637
C 0.364788 2.909388 3.623704
C -0.992225 2.618547 3.587875
H -1.717663 3.405092 3.402925
C -1.415505 1.312315 3.810813
H -2.474156 1.073893 3.811291
H 0.701136 3.933570 3.476573
H -6.377217 -0.581586 -3.469267
H -3.924826 -0.144560 -3.615005
H -1.965902 5.068605 -1.474078
H -1.461874 2.760416 0.445890
H -2.160252 6.494920 0.576057
H -4.200907 5.878107 -0.623816
H 6.013224 2.493788 -0.124681
H 5.073995 0.182046 0.442225
H 5.741320 -1.744177 -0.876256
H 3.008457 0.378307 5.814802
H 3.870813 0.816064 2.909949
H 2.883050 2.822361 4.976763
H -2.112247 -3.448869 -0.730072
H -3.771500 -3.796126 1.752648
H -5.143567 -5.578347 0.829927
H -6.750373 -4.128626 -0.434004
H 4.654657 2.848153 -2.192608
H 1.475731 -1.251265 4.945048
H -3.254372 0.750542 -0.438592
H -1.742248 2.038511 -3.751136
H -0.890871 0.760820 0.598924
H 0.372968 1.112242 1.331131
H 1.802392 0.466020 0.925154
C 0.272234 -1.191545 -1.914744
C 1.629667 -1.116317 -1.266100
C 1.898380 -2.512434 -0.885861
C 2.982975 -3.145871 -0.298595
C 2.905415 -4.510977 0.004633
C 1.749839 -5.236586 -0.272157
C 0.652555 -4.621348 -0.889267
C 0.769378 -3.279549 -1.196736
H 3.871808 -2.574242 -0.047025
H 3.753244 -5.006800 0.468755
H 1.701358 -6.293228 -0.027491
H -0.244791 -5.176581 -1.143398
O -0.187639 -2.549602 -1.840696
H 2.392478 -0.624275 -1.862068
C 0.125493 -0.665362 -3.331412
H 0.751673 -1.258835 -4.001342
H -0.912735 -0.715058 -3.674968
H 0.465140 0.372433 -3.367535
H -0.532640 -0.594423 -1.303655

149

TS^{PT}_{trans}(si)

Ru 0.550726 0.079538 -0.007344
C 0.922793 1.689274 -1.242637
C 0.597027 3.339618 -2.884083
C 1.987184 3.499826 -2.289515
H 0.634543 2.913998 -3.897070
H 0.046948 4.280377 -2.928362
H 2.762887 3.624086 -3.049920
H 2.031645 4.363114 -1.610116
C 0.134713 -1.404017 1.388962
C -0.954722 -3.140777 2.551575
C 0.471520 -3.007968 3.076095
H -1.699118 -2.855629 3.306346
H -1.170266 -4.160589 2.229853
H 0.515418 -2.906382 4.165901
H 1.089034 -3.867023 2.785906
N -0.967714 -2.213063 1.422009
N 0.937887 -1.804856 2.402492
N 2.135566 2.248770 -1.550231
N -0.000325 2.384200 -1.955539
C 2.174873 -1.167491 2.822163
H 2.412702 -0.470937 2.019144
C -2.228969 -1.825828 0.789743
H -1.927481 -1.110064 0.026334
C -1.444617 2.235480 -1.793642
H -1.590145 1.197030 -1.495105
C 3.319143 2.038395 -0.727415
H 3.110156 1.111744 -0.193682
C 4.596861 1.844434 -1.587345
C 4.471756 0.607551 -2.460172
C 5.857962 1.747885 -0.716508
C 5.029403 -0.618406 -2.064768
C 5.848687 0.459164 0.105221
H 6.737335 1.746723 -1.372829
C 5.895750 -0.751617 -0.826610
H 6.701419 0.431782 0.790820
H 6.931245 -0.898995 -1.162676
C -1.940124 3.113763 -0.612951
C -1.561985 4.591316 -0.763427
C -3.419621 2.910096 -0.313461
C -2.229879 5.439905 0.313146
H -0.472978 4.687849 -0.699187
C -4.263048 3.977982 0.033260
C -3.743262 5.397040 0.127093
H -1.963951 5.043956 1.301885
H -4.250343 5.916466 0.947738
C -2.184539 2.449094 -3.111076
H -2.112522 3.477650 -3.478485
H -3.243570 2.221520 -2.970505
C 3.442762 3.150430 0.318082
H 3.792077 4.096733 -0.110902
H 2.462667 3.304024 0.779676
H 4.140351 2.866185 1.109341
C 3.761255 0.668437 -3.663364
H 3.340930 1.618870 -3.978538
C 4.831663 -1.746721 -2.867273
C 4.107516 -1.677966 -4.049618
H 3.959674 -2.570150 -4.649647
C 3.576805 -0.456301 -4.456868
H 3.016506 -0.382117 -5.383720
H 5.251094 -2.697908 -2.549007
C -3.957916 1.618056 -0.329374
C -5.609023 3.727423 0.314905
H -6.250846 4.563100 0.585286
C -6.135421 2.443872 0.260935
C -5.298436 1.379224 -0.058477
H -7.185853 2.274411 0.477470
H -5.680543 0.364115 -0.105728
C -2.956391 -2.984994 0.051439
C -3.542431 -4.064075 0.995704
C -3.987108 -2.371098 -0.894483
C -4.899591 -4.611321 0.557906
H -2.829055 -4.889045 1.086339
C -5.368021 -2.545777 -0.729896
C -5.879043 -3.456928 0.358969
H -4.803298 -5.166384 -0.383297
H -5.981847 -2.904210 1.303693
C -3.125508 -1.055716 1.761020
H -4.087693 -0.829386 1.295395
H -3.325853 -1.601997 2.688155
H -2.634292 -0.113349 2.009904
C 3.298437 -2.200926 2.948823
H 3.248246 -2.907029 2.118488
H 4.277625 -1.716333 2.918990
H 3.232991 -2.766347 3.886254
C 1.971176 -0.358522 4.143350
C 0.908735 0.719389 3.994821
C 3.289193 0.256945 4.636275
C 1.255106 2.059426 3.765840
C 3.716684 1.412787 3.731789

H 4.071884 -0.502691 4.706110
C 2.690977 2.538577 3.826465
H 4.707377 1.782785 4.016441
H 2.864093 3.290012 3.050652
C -3.537140 -1.595939 -1.968671
H -2.469380 -1.494708 -2.125497
C -6.252455 -1.925882 -1.617230
H -7.321072 -2.077310 -1.484840
C -4.420421 -0.971847 -2.839234
C -5.791499 -1.130302 -2.658581
C -0.444878 0.381621 4.070110
H -0.720969 -0.649056 4.278914
C 0.244967 2.998828 3.546077
C -1.095791 2.643388 3.588777
H -1.867276 3.387393 3.413996
C -1.442418 1.327762 3.878785
H -2.486484 1.040584 3.949202
H 0.523368 4.032168 3.349680
H -6.494911 -0.648496 -3.330136
H -4.038620 -0.367561 -3.656761
H -1.868804 4.976178 -1.747035
H -1.386971 2.725763 0.253993
H -1.871267 6.473773 0.270722
H -4.005542 5.943319 -0.790121
H 5.952716 2.626004 -0.072835
H 4.946971 0.418247 0.728441
H 5.627384 -1.664024 -0.281985
H 3.138557 0.640705 5.653343
H 3.793936 1.066306 2.693538
H 2.830969 3.056794 4.785978
H -2.196337 -3.448670 -0.583521
H -3.667649 -3.651571 2.003587
H -5.274054 -5.312664 1.311041
H -6.877036 -3.824098 0.098267
H 4.704340 2.721306 -2.240493
H 1.628604 -1.073450 4.905062
H -3.320150 0.771764 -0.557895
H -1.788091 1.781772 -3.883087
H -0.656017 0.910823 0.698100
H 1.201845 0.922796 1.141340
H 2.066459 -0.506640 -0.142172
C 0.194550 -1.319728 -1.967806
C 1.591930 -1.296718 -1.360014
C 1.790841 -2.715123 -0.951845
C 2.856561 -3.376433 -0.365829
C 2.719379 -4.723132 -0.015496
C 1.520994 -5.394346 -0.255649
C 0.444440 -4.747909 -0.868103
C 0.617204 -3.418185 -1.216146
H 3.780592 -2.842743 -0.165712
H 3.548577 -5.247660 0.449102
H 1.423833 -6.438977 0.023902
H -0.486026 -5.263527 -1.080435
O -0.320553 -2.661148 -1.842654
H 2.339494 -0.912038 -2.049506
C 0.091892 -0.861324 -3.411101
H 0.708646 -1.502130 -4.046716
H -0.942664 -0.897967 -3.766205
H 0.456598 0.165990 -3.486668
H -0.567074 -0.676212 -1.382646

149

int$^{2}_{trans}$(si)

Ru 0.556581 0.263632 0.101195
C 0.853576 2.129543 -0.681310
C 0.520175 4.076278 -1.956961
C 1.867361 4.169034 -1.256028
H 0.634833 3.870054 -3.031375
H -0.075699 4.982735 -1.844269
H 2.672397 4.498176 -1.919252
H 1.819919 4.863498 -0.404825
C 0.298639 -1.629780 0.943385
C -0.564670 -3.768730 1.408003
C 0.908099 -3.727627 1.808652
H -1.229357 -3.826392 2.280500
H -0.775809 -4.613070 0.750269
H 1.084636 -4.087257 2.827292
H 1.527907 -4.318616 1.121278
N -0.729435 -2.494997 0.710398
N 1.218564 -2.312860 1.667732
N 2.053228 2.794066 -0.800715
N -0.070032 2.926208 -1.279738
C 2.380747 -1.716326 2.295041
H 2.461780 -0.741244 1.813482
C -2.044527 -2.009816 0.296593
H -1.818216 -1.068654 -0.206128
C -1.505315 2.667335 -1.234461
H -1.590768 1.584363 -1.132348
C 3.181401 2.461851 0.059917
H 2.963781 1.443593 0.391285
C 4.530966 2.478339 -0.703479
C 4.545143 1.503746 -1.869287
C 5.713797 2.178756 0.230813
C 5.153567 0.243859 -1.758565
C 5.673557 0.725126 0.701297
H 6.647156 2.351403 -0.319697
C 5.847741 -0.217610 -0.490690
H 6.459554 0.535817 1.439068
H 6.917939 -0.317811 -0.715341
C -2.116586 3.284625 0.054189
C -1.891336 4.801504 0.145486
C -3.573645 2.879608 0.231253
C -2.812205 5.457481 1.168774
H -0.840765 4.991722 0.390898
C -4.585580 3.809694 0.512938
C -4.261099 5.272296 0.725820
H -2.665249 4.995868 2.153443
H -4.951694 5.693942 1.464226
C -2.202275 3.075005 -2.528767
H -2.155190 4.151393 -2.721957
H -3.256543 2.793460 -2.471225
C 3.188111 3.365127 1.298548
H 3.539811 4.378314 1.071895
H 2.172482 3.422778 1.699985
H 3.828214 2.959108 2.083484
C 3.965344 1.865597 -3.090425
H 3.501851 2.843050 -3.182107
C 5.169886 -0.604541 -2.871611
C 4.586523 -0.236413 -4.076001
H 4.606512 -0.916530 -4.921455
C 3.976566 1.011692 -4.185662
H 3.517664 1.319238 -5.120105
H 5.653003 -1.574972 -2.784991
C -3.914339 1.525065 0.144404
C -5.905148 3.368468 0.644581
H -6.681825 4.097126 0.866294
C -6.235912 2.026871 0.508536
C -5.228364 1.097089 0.269499
H -7.268684 1.706141 0.606282
H -5.462391 0.042627 0.165118
C -2.749532 -2.920674 -0.746976
C -3.272929 -4.260671 -0.169703
C -3.817900 -2.097912 -1.464211
C -4.627058 -4.701121 -0.723469
H -2.534784 -5.047073 -0.355572
C -5.187680 -2.373164 -1.356254
C -5.643492 -3.572913 -0.562970
H -4.541320 -4.955603 -1.787067
H -5.732920 -3.316927 0.502395
C -2.911557 -1.654323 1.504856
H -3.903881 -1.329368 1.179667
H -3.042933 -2.491551 2.197096
H -2.435798 -0.837566 2.052673
C 3.642824 -2.531516 2.014133
H 3.716710 -2.750357 0.947868
H 4.538512 -1.981060 2.313636
H 3.640524 -3.484457 2.556240
C 2.178193 -1.493401 3.829477
C 0.789861 -0.994090 4.179478
C 3.229077 -0.518311 4.385050
C 0.520361 0.372945 4.299936
C 2.994048 0.893324 3.847993
H 4.242098 -0.852814 4.138941
C 1.604739 1.425859 4.211893
H 3.763344 1.574791 4.227662
H 1.298884 2.183161 3.482425
C -3.413445 -1.030823 -2.273919
H -2.352411 -0.834947 -2.386563
C -6.108254 -1.573421 -2.039540
H -7.167747 -1.803387 -1.955796
C -4.332904 -0.234488 -2.942361
C -5.693300 -0.502409 -2.820192
C -0.244544 -1.908260 4.405523
H -0.024610 -2.972162 4.364972
C -0.786193 0.785650 4.578751
C -1.812059 -0.128290 4.768561
H -2.819091 0.217999 4.979421
C -1.534405 -1.491735 4.699034
H -2.319377 -2.222649 4.868233
H -0.993527 1.850635 4.653227
H -6.423893 0.113154 -3.335175
H -3.987573 0.590689 -3.557875
H -2.085793 5.277721 -0.825648
H -1.552234 2.810907 0.868748
H -2.576290 6.522135 1.270024
H -4.424451 5.833130 -0.205603
H 5.722652 2.866617 1.079507
H 4.721791 0.521299 1.205864
H 5.500646 -1.224017 -0.226996
H 3.158610 -0.514443 5.479692
H 3.092960 0.887057 2.758914
H 1.652041 1.929122 5.186995
H -1.986435 -3.139413 -1.498751
H -3.372666 -4.185257 0.918995
H -4.961603 -5.603438 -0.200588
H -6.638215 -3.884975 -0.897460

H 4.669962 3.490255 -1.109644
H 2.315775 -2.467627 4.320208
H -3.132243 0.790399 -0.016792
H -1.752050 2.558725 -3.382486
H -0.681252 0.789005 0.919687
H 1.146787 0.789042 1.459210
H 2.167745 -0.444080 -0.719062
C 0.390988 -0.655764 -2.209471
C 1.857485 -0.839204 -1.743220
C 2.019868 -2.331331 -1.769389
C 3.097232 -3.150675 -1.481949
C 2.933058 -4.538127 -1.530023
C 1.698517 -5.085493 -1.877461
C 0.612310 -4.270464 -2.201079
C 0.808017 -2.898245 -2.149462
H 4.058114 -2.716928 -1.223824
H 3.769275 -5.189961 -1.299525
H 1.579580 -6.164173 -1.910604
H -0.345944 -4.685004 -2.494525
O -0.135238 -1.980741 -2.465159
H 2.530845 -0.322227 -2.426885
C 0.269321 0.168592 -3.479302
H 0.873696 -0.276977 -4.274906
H -0.771333 0.218624 -3.812351
H 0.629101 1.182085 -3.284735
H -0.365549 -0.214923 -1.477923

149
1-C$_{trans}$-bf(re)
Ru -0.049919 0.095926 -0.225144
C 0.204240 -1.933496 0.260129
C 0.961090 -4.151506 0.254210
C -0.166662 -4.035976 1.257916
H 0.634409 -4.655432 -0.665140
H 1.824100 -4.682153 0.657062
H -0.930901 -4.804663 1.125483
H 0.206810 -4.092559 2.289357
C -0.048698 2.184009 -0.437641
C 0.641797 4.430885 -0.346841
C -0.532923 4.318825 -1.298640
H 0.350134 4.931737 0.584839
H 1.486849 4.971830 -0.776786
H -1.291228 5.080553 -1.115454
H -0.219579 4.391383 -2.349218
N 0.967713 3.023466 -0.100883
N -1.012450 2.968994 -1.010555
N -0.682811 -2.701216 0.964061
N 1.253500 -2.747428 -0.029976
C -2.401812 2.590679 -1.239444
H -2.388219 1.517294 -1.378490
C 2.076526 2.676428 0.799878
H 1.733638 1.779794 1.315806
C 2.553934 -2.357373 -0.576670
H 2.357338 -1.490219 -1.195061
C -1.813307 -2.170359 1.724892
H -1.578402 -1.118037 1.873182
C -3.134022 -2.276340 0.925752
C -4.290855 -1.561711 1.617359
C -3.466879 -3.712813 0.510525
C -5.592268 -2.086674 1.608549
C -4.863103 -3.791730 -0.095738
H -2.718330 -4.047168 -0.214565
C -5.902913 -3.416090 0.954546
H -4.925938 -3.097148 -0.943387
H -6.905060 -3.377525 0.513135
C 3.513463 -1.902012 0.552694
C 3.651197 -2.928857 1.680793
C 4.874507 -1.456766 0.023921
C 4.635885 -2.442641 2.736355
H 2.668668 -3.092622 2.134806
C 6.044042 -1.646524 0.775643
C 6.032453 -2.307234 2.138314
H 4.297341 -1.470100 3.108920
H 6.681259 -1.736931 2.812867
C 3.120285 -3.459072 -1.472249
H 3.395769 -4.360253 -0.916242
H 4.019805 -3.101784 -1.974614
C -1.903512 -2.786546 3.122488
H -2.099734 -3.862516 3.108174
H -0.978794 -2.612023 3.679537
H -2.721078 -2.308908 3.666551
C -4.084882 -0.309712 2.212029
H -3.091954 0.129594 2.216449
C -6.631871 -1.357663 2.191568
C -6.412139 -0.118940 2.776721
H -7.238802 0.437344 3.206981
C -5.125665 0.408245 2.784430
H -4.932850 1.383420 3.221079
H -7.637727 -1.770575 2.166856
C 4.988644 -0.825166 -1.222237
C 7.273223 -1.230095 0.255835
H 8.168194 -1.377578 0.855933
C 7.373740 -0.637803 -0.993511
C 6.216350 -0.432467 -1.737420
H 8.341240 -0.331673 -1.379781
H 6.267307 0.034255 -2.716760
C 3.356638 2.300580 0.015848
C 3.731545 3.340021 -1.043478
C 4.529750 1.965091 0.931066
C 5.030829 2.953779 -1.738219
H 2.918406 3.409547 -1.773344
C 5.847216 2.285312 0.572175
C 6.185054 2.960246 -0.740774
H 4.916790 1.950299 -2.163363
H 6.479920 3.999157 -0.537161
C 2.286057 3.780801 1.840075
H 3.038612 3.465069 2.562575
H 2.634249 4.720466 1.399512
H 1.358904 3.976075 2.386789
C -2.945836 3.211373 -2.525159
H -2.307009 2.945842 -3.373112
H -3.950811 2.830733 -2.718817
H -3.007386 4.302646 -2.479402
C -3.292106 2.834200 0.009321
C -4.682269 2.235639 -0.190841
C -3.324078 4.298322 0.463504
C -5.848299 2.923569 0.171184
C -4.437677 4.547174 1.474896
H -3.492611 4.961011 -0.396248
C -5.786803 4.294998 0.808560
H -4.315869 3.872446 2.332219
H -5.958477 5.066678 0.044959
C 4.316388 1.308801 2.151597
H 3.309321 1.015346 2.433695
C 6.892644 1.983297 1.449910
H 7.909190 2.231359 1.153636
C 5.359951 1.033493 3.024620
C 6.661946 1.378489 2.675647
C -4.807405 0.935010 -0.698218
H -3.916500 0.357022 -0.926324
C -7.092857 2.316641 -0.024270
C -7.201933 1.041058 -0.557007
H -8.178102 0.585406 -0.689681
C -6.045268 0.339894 -0.885108
H -6.104270 -0.676648 -1.262027
H -7.990186 2.858960 0.264533
H 7.488742 1.164613 3.346182
H 5.157044 0.546946 3.974061
H 3.997058 -3.893449 1.282169
H 3.028606 -1.015108 0.986051
H 4.659793 -3.125873 3.591927
H 6.483340 -3.305121 2.045617
H -3.421919 -4.388835 1.375536
H -5.059527 -4.794993 -0.488104
H -5.936044 -4.200851 1.723332
H -2.349418 4.560853 0.888580
H -4.387343 5.570782 1.859721
H -6.601965 4.393850 1.533461
H 3.094337 1.377602 -0.519684
H 3.848238 4.332592 -0.584378
H 5.248220 3.633973 -2.568405
H 7.063615 2.468403 -1.173469
H -2.952704 -1.741705 -0.018287
H -2.815563 2.261904 0.819708
H 4.098539 -0.650346 -1.817735
H 2.392628 -3.729590 -2.239296
H 0.247157 0.319755 1.361524
H -1.656275 0.465544 0.374562
H -1.769176 -0.261414 -0.026553
C 0.525189 -0.228311 -2.368222
O 0.844398 -1.556009 -2.756886
C -0.315741 -2.249818 -2.834214
C -0.433872 -3.605731 -3.095648
C -1.725365 -4.128171 -3.180317
C -2.846649 -3.312960 -3.008108
C -2.704273 -1.953682 -2.725616
C -1.423757 -1.419617 -2.625626
H 0.440635 -4.226695 -3.256958
H -1.858275 -5.183095 -3.399328
H -3.839274 -3.744124 -3.091738
H -3.579905 -1.324576 -2.585061
C -0.882811 -0.096697 -2.317188
C 1.491221 0.753112 -2.967771
H 1.519027 0.642910 -4.058599
H 1.186602 1.770444 -2.717204
H 2.502575 0.608031 -2.579841
H 1.538883 0.145206 -0.193391
H -1.370251 0.807735 -2.652098

149
TS$^{HT}_{trans}$(re)
Ru 0.039642 0.084879 -0.100591
C -0.080249 2.162958 -0.312835
C -0.675849 4.252235 -1.208075
C 0.487943 4.449997 -0.259265
H -0.358790 4.306553 -2.259514

H -1.470647 4.981525 -1.054965
H 1.299309 5.033563 -0.699211
H 0.167374 4.945724 0.666296
C 0.197679 -1.962496 0.370013
C -0.231263 -4.045387 1.382428
C 0.894607 -4.207665 0.381488
H 0.135813 -4.108101 2.415731
H -1.021368 -4.788051 1.253955
H 1.747103 -4.746022 0.798435
H 0.559854 -4.726259 -0.527427
N -0.702565 -2.694893 1.083184
N 1.217809 -2.817348 0.068770
N 0.893537 3.067645 0.000479
N -1.089939 2.890133 -0.878968
C 2.522029 -2.463874 -0.491383
H 2.330453 -1.652685 -1.182706
C -1.843043 -2.139339 1.811980
H -1.600786 -1.087264 1.933139
C -2.443549 2.425950 -1.163553
H -2.364707 1.345859 -1.199067
C 2.048570 2.794084 0.864867
H 1.751709 1.931838 1.459773
C 3.292096 2.389886 0.037278
C 4.498319 2.059653 0.910160
C 3.630241 3.412817 -1.050228
C 5.801999 2.373591 0.498657
C 4.899519 3.007611 -1.788480
H 2.788080 3.481533 -1.746582
C 6.090639 3.030022 -0.835365
H 4.768577 1.994855 -2.187021
H 6.953163 2.532796 -1.293308
C -3.422720 2.701612 0.009383
C -3.498871 4.176501 0.421382
C -4.792258 2.095248 -0.285579
C -4.691917 4.444670 1.332169
H -2.562689 4.458299 0.915105
C -5.985122 2.792227 -0.051498
C -5.980170 4.176512 0.560391
H -4.642615 3.786935 2.209305
H -6.853448 4.286376 1.212534
C -2.927819 2.942345 -2.518732
H -3.073108 4.027235 -2.529784
H -3.883493 2.479606 -2.771744
C 2.301524 3.955241 1.830195
H 2.595910 4.878341 1.321781
H 1.411358 4.160350 2.431389
H 3.110288 3.687002 2.511064
C 4.329768 1.416443 2.144569
H 3.334257 1.127440 2.468620
C 6.878738 2.081094 1.340805
C 6.693202 1.489068 2.580513
H 7.544074 1.281789 3.222340
C 5.405049 1.147925 2.980660
H 5.237131 0.670139 3.941286
H 7.883833 2.325311 1.004823
C -4.872032 0.780860 -0.765213
C -7.207931 2.182462 -0.349522
H -8.126797 2.732796 -0.160330
C -7.269871 0.894882 -0.859983
C -6.088880 0.183896 -1.055357
H -8.230362 0.438130 -1.077565
H -6.116557 -0.841664 -1.411857
C -3.133857 -2.217871 0.970901
C -3.492891 -3.644768 0.549212
C -4.293152 -1.477024 1.625363
C -4.863955 -3.688543 -0.115705
H -2.723973 -4.011785 -0.137674
C -5.607247 -1.963315 1.567783
C -5.936544 -3.275376 0.887579
H -4.873556 -2.998625 -0.970418
H -6.037657 -4.064290 1.646159
C -1.977089 -2.745592 3.209129
H -2.796704 -2.247354 3.731329
H -2.196796 -3.817658 3.198199
H -1.062498 -2.588306 3.788415
C 3.116178 -3.619139 -1.297728
H 2.391685 -3.993313 -2.022025
H 3.992312 -3.268195 -1.844945
H 3.436439 -4.456876 -0.671569
C 3.476682 -1.922768 0.600673
C 4.805743 -1.445729 0.024054
C 3.665339 -2.897912 1.765820
C 6.002275 -1.584800 0.743006
C 4.671914 -2.348390 2.768779
H 4.019445 -3.872472 1.401292
C 6.046119 -2.216368 2.118940
H 4.329086 -1.365353 3.109319
H 6.501075 -3.213090 2.033105
C -4.069215 -0.232828 2.229819
H -3.065362 0.180289 2.245337
C -6.645469 -1.205745 2.116306
H -7.662540 -1.585196 2.048932
C -5.107885 0.510114 2.772539
C -6.409249 0.022011 2.717054
C 4.858175 -0.830000 -1.234487
H 3.943133 -0.693335 -1.802596
C 7.200700 -1.139340 0.177879
C 7.242440 -0.565328 -1.083612
H 8.187132 -0.235315 -1.505082
C 6.056465 -0.406854 -1.793461
H 6.063052 0.045897 -2.780697
H 8.118300 -1.246717 0.751588
H -7.233816 0.600976 3.120831
H -4.902137 1.476991 3.222387
H -3.604522 4.816329 -0.465971
H -3.001553 2.142502 0.857254
H -4.673894 5.476206 1.698790
H -6.088385 4.933308 -0.229538
H 3.772363 4.410418 -0.610541
H 5.087140 3.669769 -2.640178
H 6.391151 4.072197 -0.658687
H 2.697139 -3.062414 2.250394
H 4.735850 -2.992893 3.651800
H 6.713663 -1.626271 2.757010
H -2.884673 -1.665591 0.053967
H -3.507546 -4.314410 1.420744
H -5.075426 -4.688739 -0.509077
H -6.913985 -3.193864 0.398612
H 3.002880 1.461650 -0.474071
H 2.969891 -1.036902 1.006468
H -3.960650 0.200818 -0.880389
H -2.206016 2.694156 -3.303862
H -0.258227 0.341880 1.437424
H 1.350041 0.221382 0.774404
H 1.509404 0.038192 -0.841291
C 0.635592 -0.173727 -2.505969
O 1.056788 -1.430549 -2.911309
C -0.038067 -2.253158 -2.899101
C -0.045856 -3.599404 -3.220971
C -1.282326 -4.244606 -3.191074
C -2.451942 -3.550815 -2.864289
C -2.415192 -2.196050 -2.543558
C -1.186991 -1.542493 -2.548778
H 0.859019 -4.117551 -3.516306
H -1.337043 -5.297957 -3.446612
H -3.402154 -4.075794 -2.859825
H -3.325066 -1.659187 -2.292418
C -0.748448 -0.182029 -2.266540
C 1.456675 0.942910 -3.077556
H 1.334799 0.962125 -4.166263
H 1.122845 1.891660 -2.656752
H 2.517517 0.821149 -2.847306
H -1.580822 0.055739 0.160916
H -1.349496 0.686301 -2.476112

149

int$^{1}_{trans}$(re)

Ru -0.007572 0.173498 -0.301224
C 0.116019 2.211107 0.094355
C 0.761257 4.265987 1.034620
C -0.392043 4.514057 0.072186
H 0.446844 4.375036 2.081459
H 1.602056 4.940644 0.863001
H -1.205899 5.075948 0.536708
H -0.064867 5.059136 -0.822310
C -0.034900 -1.865516 -0.732529
C 0.515977 -3.948097 -1.674820
C -0.601466 -4.141388 -0.663666
H 0.155834 -4.064304 -2.706301
H 1.342131 -4.645732 -1.522384
H -1.427579 -4.731476 -1.064947
H -0.239181 -4.614840 0.258077
N 0.911244 -2.563584 -1.419825
N -0.992286 -2.760259 -0.391969
N -0.798716 3.150854 -0.267669
N 1.103711 2.873599 0.750795
C -2.238428 -2.425320 0.291063
H -1.987923 -1.644566 1.007376
C 2.080782 -1.974394 -2.069013
H 1.802384 -0.940883 -2.266741
C 2.330810 2.270152 1.268096
H 2.087470 1.222281 1.400990
C -1.979539 2.876963 -1.088181
H -1.676994 2.091772 -1.784734
C -3.132589 2.314722 -0.216431
C -4.400116 2.014512 -1.006674
C -3.405866 3.203973 0.999948
C -5.670151 2.230766 -0.451608
C -4.590278 2.681903 1.800501
H -2.508426 3.240115 1.622778
C -5.859625 2.751561 0.957659
H -4.394176 1.644003 2.091191
H -6.666352 2.185332 1.435413
C 3.474232 2.308589 0.224915
C 3.785930 3.720327 -0.279673
C 4.722697 1.583571 0.722339

C 4.985609 3.707687 -1.218901
H 2.905481 4.113993 -0.798574
C 6.008358 2.041223 0.399253
C 6.236257 3.260313 -0.469735
H 4.783501 3.012510 -2.040604
H 7.045542 3.045085 -1.176751
C 2.688565 2.853990 2.634309
H 2.947102 3.916458 2.586891
H 3.547943 2.325354 3.049444
C -2.371786 4.101248 -1.916728
H -2.693943 4.943794 -1.298291
H -1.535729 4.430706 -2.539551
H -3.201851 3.844177 -2.575789
C -4.319689 1.494218 -2.305050
H -3.346631 1.283806 -2.740395
C -6.806805 1.965181 -1.220657
C -6.712963 1.493458 -2.521123
H -7.610918 1.302489 -3.100784
C -5.455641 1.248784 -3.064490
H -5.358422 0.859780 -4.073679
H -7.785405 2.130465 -0.776657
C 4.609517 0.429107 1.511685
C 7.126506 1.365263 0.897458
H 8.116319 1.730484 0.633137
C 6.999258 0.253592 1.715399
C 5.726273 -0.218907 2.020692
H 7.881504 -0.245869 2.104247
H 5.601811 -1.092188 2.653733
C 3.288808 -1.949051 -1.103641
C 3.668938 -3.341604 -0.592700
C 4.492558 -1.192908 -1.654407
C 4.855314 -3.251577 0.358462
H 2.808253 -3.779824 -0.077349
C 5.798602 -1.554285 -1.287430
C 6.086971 -2.728876 -0.375249
H 4.594763 -2.572631 1.178098
H 6.515234 -3.540050 -0.980411
C 2.372227 -2.643035 -3.412895
H 3.211196 -2.136929 -3.894569
H 2.639956 -3.698881 -3.312648
H 1.505252 -2.571588 -4.076146
C -2.767729 -3.621410 1.081914
H -1.983985 -4.030459 1.722026
H -3.587397 -3.303050 1.726328
H -3.149692 -4.414622 0.432388
C -3.260094 -1.835794 -0.717586
C -4.595860 -1.470402 -0.079111
C -3.435674 -2.738616 -1.943055
C -5.795290 -1.588144 -0.798438
C -4.478393 -2.168649 -2.895057
H -3.746955 -3.744298 -1.627953
C -5.843254 -2.118508 -2.216498
H -4.174560 -1.158165 -3.188556
H -6.268885 -3.131424 -2.192042
C 4.323468 -0.093145 -2.506125
H 3.323751 0.224866 -2.785016
C 6.880065 -0.824925 -1.788429
H 7.884712 -1.108025 -1.483753
C 5.407726 0.608342 -3.017667
C 6.699797 0.242273 -2.655846
C -4.653331 -0.992785 1.237891
H -3.747543 -0.906804 1.830795
C -7.001154 -1.237414 -0.184865
C -7.047125 -0.783295 1.124648
H -7.997493 -0.525033 1.582190
C -5.860353 -0.662517 1.840402
H -5.868486 -0.312129 2.868256
H -7.921356 -1.326189 -0.757577
H 7.556694 0.786681 -3.040897
H 5.241983 1.437857 -3.698660
H 3.997366 4.391263 0.565276
H 3.090847 1.736581 -0.632596
H 5.146001 4.696781 -1.660620
H 6.591826 4.083963 0.165062
H -3.618093 4.231808 0.672749
H -4.718981 3.257325 2.722990
H -6.199998 3.795171 0.903974
H -2.470924 -2.840818 -2.449651
H -4.537119 -2.766906 -3.810467
H -6.537919 -1.505835 -2.801705
H 2.933443 -1.376099 -0.235135
H 3.923221 -4.004170 -1.431734
H 5.072076 -4.226899 0.806338
H 6.861633 -2.436865 0.342465
H -2.764851 1.349930 0.168116
H -2.807126 -0.897640 -1.082730
H 3.627841 0.029554 1.750580
H 1.848859 2.734458 3.325615
H 0.435585 0.614269 -1.860422
H -0.444327 0.366643 -1.936371
H -2.095619 0.479991 1.822067
C -1.278222 0.298316 2.535106
O -1.756783 -0.788531 3.382645
C -0.865680 -1.815582 3.297147
C -0.973267 -3.001403 4.004149
C 0.004127 -3.978796 3.782270
C 1.047394 -3.752760 2.887229
C 1.135293 -2.537207 2.197552
C 0.173497 -1.559676 2.400214
H -1.793703 -3.163165 4.695011
H -0.056342 -4.922084 4.316754
H 1.802014 -4.518230 2.729581
H 1.941929 -2.356340 1.492990
C 0.023809 -0.180092 1.838718
C -1.103379 1.541943 3.389427
H -0.376801 1.345515 4.184524
H -0.720737 2.356714 2.766680
H -2.047948 1.857521 3.843310
H 1.532108 0.117942 -0.363819
H 0.832698 0.414577 2.276114

149

TS^{PT}_{trans}(re)

Ru 0.003903 0.088696 -0.083652
C 0.076557 2.131106 0.277251
C 0.684258 4.203884 1.216054
C -0.473745 4.422472 0.259145
H 0.368149 4.291745 2.265250
H 1.502757 4.906461 1.049535
H -1.288181 4.994484 0.709318
H -0.149510 4.943634 -0.650543
C -0.035049 -1.919577 -0.687972
C 0.542340 -3.929388 -1.772671
C -0.587349 -4.204840 -0.794323
H 0.202717 -3.999459 -2.814204
H 1.382790 -4.615197 -1.642200
H -1.410165 -4.752345 -1.258364
H -0.242107 -4.766974 0.083125
N 0.907201 -2.553858 -1.437495
N -0.978336 -2.854110 -0.401180
N -0.877440 3.049415 -0.045180
N 1.063830 2.826301 0.913909
C -2.264566 -2.578642 0.232933
H -2.062915 -1.887506 1.044636
C 2.037095 -1.896410 -2.095434
H 1.696426 -0.878732 -2.280939
C 2.338176 2.278357 1.367238
H 2.152806 1.223404 1.521721
C -2.042922 2.782257 -0.892738
H -1.742172 1.932195 -1.508817
C -3.261619 2.337052 -0.044813
C -4.503626 2.066039 -0.887688
C -3.560417 3.299986 1.107351
C -5.787607 2.364684 -0.409398
C -4.793741 2.843931 1.876202
H -2.690844 3.347652 1.768925
C -6.025081 2.921250 0.978925
H -4.644209 1.808272 2.203872
H -6.863149 2.382822 1.435593
C 3.420269 2.357866 0.262161
C 3.640932 3.781371 -0.257492
C 4.724540 1.687681 0.686160
C 4.786670 3.822527 -1.260532
H 2.716648 4.133736 -0.726983
C 5.969314 2.199879 0.292137
C 6.095006 3.429434 -0.582848
H 4.570033 3.121082 -2.072608
H 6.872821 3.250909 -1.333960
C 2.747982 2.882920 2.710550
H 2.970510 3.952194 2.645642
H 3.644152 2.383861 3.082893
C -2.327476 3.969532 -1.815240
H -2.627682 4.869977 -1.270279
H -1.445612 4.207101 -2.416660
H -3.139270 3.718256 -2.498150
C -4.384473 1.510080 -2.168769
H -3.402795 1.232970 -2.542517
C -6.895186 2.151234 -1.235688
C -6.758446 1.649897 -2.520738
H -7.632288 1.503980 -3.148493
C -5.489369 1.318898 -2.986428
H -5.359716 0.904985 -3.982033
H -7.884910 2.385697 -0.850506
C 4.707067 0.526706 1.473280
C 7.141346 1.569196 0.720852
H 8.098055 1.976001 0.401353
C 7.108782 0.449183 1.536766
C 5.876687 -0.077746 1.912135
H 8.032254 -0.015469 1.868691
H 5.825854 -0.961065 2.541696
C 3.253958 -1.825397 -1.143150
C 3.676077 -3.206309 -0.633185
C 4.427239 -1.040939 -1.718862
C 4.896446 -3.093516 0.271566
H 2.838332 -3.654214 -0.088817
C 5.751638 -1.384367 -1.404627

C 6.091029 -2.562385 -0.514905
H 4.666131 -2.406142 1.094114
H 6.493440 -3.369273 -1.143123
C 2.348094 -2.534810 -3.450085
H 3.156002 -1.980309 -3.930952
H 2.669176 -3.577597 -3.369760
H 1.474473 -2.493383 -4.106561
C -2.860279 -3.833495 0.871121
H -2.130532 -4.313415 1.526024
H -3.722831 -3.551421 1.477315
H -3.203282 -4.563179 0.132496
C -3.219739 -1.877639 -0.765692
C -4.549389 -1.478859 -0.136248
C -3.394440 -2.696385 -2.049112
C -5.741395 -1.518266 -0.876443
C -4.415992 -2.053364 -2.977187
H -3.726550 -3.715914 -1.809236
C -5.785455 -2.014515 -2.306667
H -4.089364 -1.035626 -3.213824
H -6.213994 -3.026740 -2.310464
C 4.208067 0.064443 -2.552131
H 3.192894 0.367524 -2.788950
C 6.802309 -0.633435 -1.938467
H 7.822067 -0.902896 -1.674282
C 5.261681 0.785919 -3.098653
C 6.572345 0.437118 -2.789690
C -4.604434 -1.026651 1.189985
H -3.705154 -1.002465 1.797818
C -6.936553 -1.113569 -0.276213
C -6.980443 -0.681814 1.041225
H -7.922698 -0.376881 1.486601
C -5.802257 -0.641347 1.779151
H -5.811818 -0.313957 2.814734
H -7.849324 -1.136299 -0.866540
H 7.405361 0.997669 -3.203051
H 5.057658 1.617535 -3.766534
H 3.868276 4.462582 0.575119
H 3.011191 1.768474 -0.570857
H 4.879205 4.818635 -1.705779
H 6.448613 4.266611 0.035176
H -3.730957 4.314182 0.718642
H -4.944002 3.451523 2.774666
H -6.341781 3.969665 0.889237
H -2.423619 -2.779174 -2.548380
H -4.478042 -2.600699 -3.923770
H -6.474834 -1.385198 -2.879919
H 2.890264 -1.257925 -0.274390
H 3.913405 -3.870983 -1.475567
H 5.142427 -4.061486 0.720857
H 6.897226 -2.270228 0.167151
H -2.967998 1.377830 0.409215
H -2.706328 -0.946129 -1.054527
H 3.757694 0.084995 1.763585
H 1.949370 2.744784 3.446365
H 1.237735 0.368647 -0.967142
H -0.619937 0.384276 -1.574159
H -1.718252 0.049708 1.468198
C -1.098635 -0.030298 2.397785
O -1.635598 -1.164223 3.128930
C -0.695241 -2.151728 3.130905
C -0.885774 -3.416623 3.662441
C 0.166128 -4.329311 3.539956
C 1.358706 -3.974656 2.910446
C 1.527453 -2.685322 2.397890
C 0.493586 -1.771265 2.516068
H -1.822065 -3.684736 4.139117
H 0.046158 -5.331325 3.940340
H 2.161218 -4.700241 2.821994
H 2.450347 -2.402480 1.898793
C 0.371287 -0.350045 2.071024
C -1.324477 1.223836 3.218003
H -0.823259 1.127300 4.185565
H -0.913259 2.085669 2.688194
H -2.391556 1.391983 3.388542
H 1.240281 -0.310243 0.844309
H 1.010971 0.298312 2.680610

149

int$^{2}_{trans}$(re)

Ru 0.066905 0.268139 -0.414424
C 0.183448 2.248360 0.080646
C 0.773208 4.261742 1.165530
C -0.280703 4.565726 0.105268
H 0.365548 4.359974 2.182342
H 1.641096 4.920486 1.089645
H -1.123263 5.128369 0.514978
H 0.137176 5.138663 -0.732992
C 0.041231 -1.710072 -1.021129
C 0.565791 -3.694705 -2.171545
C -0.531265 -3.993626 -1.159165
H 0.181945 -3.705300 -3.201186
H 1.392467 -4.405927 -2.112326
H -1.373536 -4.524351 -1.608098
H -0.159595 -4.588013 -0.313034
N 0.969031 -2.344862 -1.786391
N -0.896412 -2.650771 -0.716080
N -0.672894 3.226017 -0.322890
N 1.105378 2.870153 0.876596
C -2.131541 -2.371074 0.005062
H -1.868365 -1.636764 0.770493
C 2.161458 -1.701721 -2.330887
H 1.889683 -0.647818 -2.404775
C 2.261954 2.204681 1.465187
H 1.958792 1.164887 1.564428
C -1.848307 2.964338 -1.145739
H -1.532091 2.197202 -1.860030
C -2.986889 2.365545 -0.270988
C -4.274822 2.102099 -1.039544
C -3.232104 3.202275 0.988417
C -5.530409 2.289731 -0.441704
C -4.392125 2.642406 1.799118
H -2.317758 3.220274 1.587459
C -5.684280 2.743395 0.995052
H -4.187115 1.593135 2.040598
H -6.476106 2.152661 1.468011
C 3.470246 2.223025 0.495163
C 3.894311 3.643253 0.107670
C 4.642284 1.384815 0.994786
C 5.131979 3.619143 -0.779745
H 3.067093 4.126214 -0.422208
C 5.969153 1.761021 0.735371
C 6.316151 3.022286 -0.027260
H 4.918262 3.011310 -1.664072
H 7.134067 2.801247 -0.722205
C 2.571883 2.745725 2.860704
H 2.862234 3.800526 2.849479
H 3.400004 2.182192 3.295481
C -2.256405 4.210948 -1.930753
H -2.609279 5.019453 -1.283387
H -1.413295 4.583449 -2.518823
H -3.067029 3.968422 -2.618772
C -4.228069 1.652320 -2.365438
H -3.265940 1.466763 -2.834853
C -6.687008 2.062786 -1.192927
C -6.626983 1.660553 -2.518602
H -7.539711 1.500338 -3.084442
C -5.384114 1.448276 -3.106964
H -5.313584 1.116673 -4.138620
H -7.653692 2.204379 -0.715718
C 4.419051 0.194982 1.702468
C 7.016271 0.961987 1.203461
H 8.038218 1.262283 0.984009
C 6.780951 -0.190285 1.937967
C 5.467399 -0.574973 2.188218
H 7.610711 -0.786416 2.305477
H 5.258559 -1.475152 2.758628
C 3.336108 -1.815641 -1.326129
C 3.649956 -3.264315 -0.939543
C 4.584000 -1.056035 -1.759245
C 4.819588 -3.321216 0.034811
H 2.760993 -3.708531 -0.479626
C 5.865913 -1.505962 -1.405575
C 6.087292 -2.781474 -0.619231
H 4.574618 -2.713180 0.912027
H 6.493692 -3.542856 -1.299692
C 2.480050 -2.219605 -3.732980
H 3.333502 -1.674205 -4.140296
H 2.735600 -3.283866 -3.745195
H 1.627377 -2.064461 -4.400415
C -2.641601 -3.618404 0.724504
H -1.848356 -4.058643 1.331522
H -3.463820 -3.354028 1.389789
H -3.012746 -4.376083 0.028177
C -3.175596 -1.720457 -0.942347
C -4.496319 -1.396746 -0.253596
C -3.375614 -2.553062 -2.213934
C -5.715591 -1.494000 -0.941562
C -4.456002 -1.951740 -3.101696
H -3.660413 -3.581111 -1.950095
C -5.799796 -1.957477 -2.380883
H -4.175699 -0.924168 -3.352685
H -6.209526 -2.977251 -2.392261
C 4.477647 0.133146 -2.493548
H 3.496549 0.519161 -2.752324
C 6.987455 -0.773887 -1.805074
H 7.972810 -1.129457 -1.512716
C 5.601264 0.834085 -2.912283
C 6.869870 0.380598 -2.564521
C -4.520380 -0.976864 1.083421
H -3.598737 -0.900355 1.651251
C -6.905260 -1.181691 -0.277306
C -6.916545 -0.786734 1.052082
H -7.854982 -0.557182 1.547843
C -5.710035 -0.685361 1.737290
H -5.688079 -0.377515 2.778490
H -7.841225 -1.252648 -0.826421

H 7.756197 0.923622 -2.878719
H 5.484969 1.733772 -3.509278
H 4.105916 4.241899 1.005126
H 3.097254 1.736377 -0.416720
H 5.379646 4.627160 -1.129016
H 6.705226 3.764716 0.683698
H -3.457813 4.241255 0.709273
H -4.497791 3.178021 2.748625
H -6.028810 3.786986 0.998613
H -2.425893 -2.605205 -2.754755
H -4.534376 -2.504806 -4.043626
H -6.521107 -1.328691 -2.914443
H 2.964466 -1.317447 -0.419336
H 3.890810 -3.859457 -1.831908
H 4.986480 -4.345241 0.385944
H 6.857906 -2.601475 0.138810
H -2.607506 1.385677 0.069156
H -2.729227 -0.763548 -1.262609
H 3.402573 -0.140924 1.884692
H 1.704613 2.640689 3.519051
H 1.614131 0.239915 -0.436037
H 0.103356 0.686818 -1.937582
H -1.778508 0.311401 1.842366
C -1.196240 0.163873 2.759696
O -1.958933 -0.750526 3.593301
C -1.358964 -1.969278 3.554236
C -1.872804 -3.120923 4.130117
C -1.131818 -4.295785 3.982520
C 0.068600 -4.314081 3.272764
C 0.570090 -3.136697 2.707884
C -0.147746 -1.964204 2.867572
H -2.816091 -3.103286 4.663794
H -1.509797 -5.215327 4.418462
H 0.616907 -5.243808 3.162420
H 1.504902 -3.136933 2.153744
C 0.137388 -0.552946 2.446000
C -1.052196 1.486567 3.478134
H -0.490016 1.358539 4.408453
H -0.514737 2.184147 2.829706
H -2.033051 1.907194 3.714314
H 0.482195 -0.522041 1.391131
H 0.955256 -0.122287 3.037751

149

1-C$_{cis}$-bf(si)

Ru 0.204907 -0.432515 0.651746
C 0.003468 -1.474722 -1.125123
C 0.293377 -2.231344 -3.333709
C -0.747136 -3.102756 -2.654857
H -0.159640 -1.553116 -4.070794
H 1.066278 -2.814570 -3.840179
H -1.657123 -3.214819 -3.247593
H -0.354817 -4.104333 -2.435765
C 0.517849 1.428253 -0.407178
C 1.649435 3.213768 -1.454219
C 0.176129 3.280248 -1.828611
H 2.019815 4.154028 -1.038229
H 2.266405 2.960147 -2.322411
H -0.321930 4.155289 -1.394687
H 0.028897 3.305608 -2.912842
N 1.682146 2.134078 -0.470347
N -0.351863 2.047496 -1.249229
N -0.990372 -2.363467 -1.417410
N 0.831557 -1.477766 -2.207234
C -1.743260 1.661333 -1.479283
H -1.923605 0.878563 -0.750108
C 2.786742 1.994786 0.479535
H 2.471798 1.153747 1.086994
C 2.053285 -0.706530 -2.378971
H 2.188310 -0.183333 -1.434786
C -2.029088 -2.800763 -0.484227
H -1.610133 -2.603839 0.500344
C -3.312648 -1.953853 -0.647933
C -4.303768 -2.174579 0.489692
C -3.950330 -2.092785 -2.033439
C -5.687492 -2.202900 0.258149
C -5.283658 -1.357084 -2.087496
H -3.259112 -1.689950 -2.781250
C -6.267537 -2.019687 -1.128480
H -5.137614 -0.309429 -1.797285
H -7.183851 -1.423975 -1.054510
C 3.307518 -1.612440 -2.604415
C 4.454612 -0.771228 -3.202408
C 3.725407 -2.377094 -1.343628
C 5.828699 -1.395855 -3.004774
H 4.257869 -0.595760 -4.264550
C 5.027158 -2.298087 -0.820808
C 6.107663 -1.499882 -1.510638
H 5.864806 -2.393770 -3.460044
H 7.080236 -1.966313 -1.323031
C 1.837989 0.320510 -3.498726
H 1.775704 -0.151696 -4.485878
H 2.648007 1.051015 -3.535021
C -2.269799 -4.307968 -0.558269
H -2.638601 -4.644580 -1.531582
H -1.349131 -4.853398 -0.331578
H -3.016954 -4.579943 0.190924
C -3.844650 -2.262204 1.811644
H -2.784205 -2.190119 2.034485
C -6.561393 -2.356741 1.338033
C -6.094692 -2.468758 2.640429
H -6.792790 -2.585176 3.463753
C -4.723894 -2.413231 2.876552
H -4.333640 -2.484711 3.887352
H -7.631864 -2.371976 1.145459
C 2.809449 -3.216280 -0.696108
C 5.349648 -3.001022 0.343011
H 6.357744 -2.914095 0.739920
C 4.412564 -3.781336 1.002692
C 3.133275 -3.896379 0.468546
H 4.681668 -4.307552 1.913549
H 2.382105 -4.508508 0.957497
C 4.136414 1.532525 -0.138250
C 4.886278 2.538243 -1.030546
C 5.024898 0.987960 0.978003
C 5.815703 3.477431 -0.260806
H 5.501776 1.970223 -1.742228
C 6.271870 1.543337 1.298521
C 6.870314 2.671426 0.487637
H 6.298498 4.173012 -0.955385
H 7.463804 3.321709 1.139279
C 2.881115 3.227697 1.378532
H 3.656004 3.078472 2.135351
H 3.124522 4.141350 0.827752
H 1.925758 3.386636 1.887305
C -1.927927 1.024499 -2.854413
H -1.285869 0.144408 -2.930450
H -2.965286 0.699158 -2.976243
H -1.681602 1.707423 -3.674562
C -2.741025 2.815909 -1.148834
C -4.063874 2.250316 -0.633744
C -2.962858 3.854016 -2.270628
C -5.291593 2.473664 -1.272048
C -4.087622 3.494326 -3.242769
H -2.042508 4.023001 -2.834153
C -5.407904 3.349361 -2.498991
H -4.172219 4.272600 -4.008683
H -6.184046 2.954174 -3.163546
C 4.566236 -0.111993 1.713721
H 3.628025 -0.582976 1.427450
C 7.002000 1.010682 2.366073
C 5.298687 -0.631090 2.771125
C 6.523554 -0.060587 3.107557
C -4.050435 1.507349 0.554666
H -3.115195 1.370893 1.090387
C -6.456371 1.918450 -0.727474
C -6.423865 1.165901 0.436485
H -7.335148 0.732313 0.836873
C -5.209267 0.965701 1.088079
H -5.159297 0.381969 2.001143
H -7.402789 2.089313 -1.235623
H 4.918706 -1.489712 3.315951
H 4.474846 0.215986 -2.725437
H 3.045806 -2.365226 -3.362347
H 6.591392 -0.785365 -3.499789
H 6.160463 -0.491708 -1.074247
H -4.117563 -3.150067 -2.280469
H -5.689519 -1.360307 -3.104717
H -6.563264 -2.998817 -1.531186
H -3.213508 4.814073 -1.801527
H -3.857843 2.562514 -3.767107
H -5.752734 4.344398 -2.184157
H 3.868894 0.671213 -0.758544
H 4.186805 3.118182 -1.635938
H 5.246876 4.083870 0.450171
H 7.573906 2.242838 -0.240504
H -2.992518 -0.907571 -0.567972
H -2.286411 3.333975 -0.294037
H 1.809913 -3.324456 -1.093595
H 0.904171 0.857272 -3.314842
C 0.272574 -0.613164 2.833228
C 1.275864 -1.549433 3.444271
H 1.406178 -1.320554 4.509238
H 0.944433 -2.584735 3.341990
H 2.231269 -1.449931 2.929792
C -1.603149 0.445004 3.464453
C -0.759913 1.440931 2.952414
C -2.884022 0.708753 3.920162
C -1.206391 2.756863 2.915777
C -3.311979 2.038146 3.885588
H -3.522365 -0.086392 4.288555
C -2.486412 3.049660 3.392377
H -0.570761 3.543294 2.517576
H -4.309715 2.283085 4.235384
H -2.848917 4.072518 3.369634
C 0.466976 0.760958 2.529849

O -1.010122 -0.776195 3.435347
H 1.784736 -0.684787 0.559810
H 0.244705 -1.986443 1.098086
H -1.468395 -0.711187 0.994660
H -1.465068 0.103980 0.778682
H 1.434320 1.234424 2.624185
H 7.964668 1.451997 2.613833
H 7.105450 -0.453016 3.935999

149
TS$^{HT}_{cis}$(si)
Ru -0.104878 0.067632 -1.206278
C 0.647239 1.431965 0.169103
C 1.793788 2.409447 1.966260
C 1.008375 3.488861 1.250552
H 1.310722 2.126224 2.910336
H 2.822112 2.694083 2.176440
H 0.431346 4.113157 1.934908
H 1.671735 4.140935 0.665674
C -0.276438 -1.428141 0.295684
C -0.122262 -3.491530 1.392457
C -0.512569 -2.433368 2.399525
H -0.988303 -4.112068 1.118073
H 0.677655 -4.142277 1.748463
H -1.329750 -2.737481 3.048480
H 0.343876 -2.145714 3.023425
N 0.304050 -2.666719 0.263763
N -0.880720 -1.329428 1.507080
N 0.142416 2.699467 0.375532
N 1.703843 1.306567 1.006650
C -1.670185 -0.202356 1.995657
H -1.576735 0.582881 1.244495
C 0.655172 -3.317739 -0.992433
H 0.944685 -2.494980 -1.642868
C 2.659550 0.204780 1.036562
H 2.247971 -0.556887 0.372479
C -0.599887 3.416461 -0.660751
H -1.142521 2.652289 -1.209915
C -1.628681 4.402255 -0.071263
C -2.630336 3.740753 0.852763
C -2.429473 5.099730 -1.186721
C -3.780269 3.153526 0.304196
C -3.199116 4.086324 -2.039492
H -3.130931 5.793313 -0.709668
C -3.969230 3.040000 -1.193529
H -3.896527 4.624156 -2.688539
H -5.040809 3.090918 -1.410733
C 4.039329 0.601253 0.409875
C 3.862401 0.885098 -1.102734
C 4.781179 1.697526 1.157157
C 4.670089 2.066330 -1.630086
H 4.102164 -0.024231 -1.661956
C 4.877299 3.003381 0.646977
C 4.392401 3.287180 -0.753770
H 5.746536 1.853712 -1.617690
H 4.885316 4.184721 -1.141468
C 2.751726 -0.389229 2.437782
H 3.092276 0.335295 3.183242
H 3.440245 -1.237833 2.438481
C 0.357708 4.088083 -1.655555
H 0.793429 5.011132 -1.257141
H 1.165892 3.395770 -1.908789
H -0.161632 4.334917 -2.584347
C -2.492445 3.804361 2.240447
H -1.614709 4.285126 2.664410
C -4.767452 2.666969 1.164834
C -4.622544 2.734907 2.545338
H -5.405449 2.352469 3.193183
C -3.472662 3.301913 3.088635
H -3.349769 3.371790 4.165276
H -5.666177 2.227744 0.739271
C 5.385320 1.416203 2.387210
C 5.489153 3.997817 1.411210
H 5.559252 5.004522 1.006151
C 6.023241 3.718477 2.664515
C 5.988863 2.413202 3.145618
H 6.491610 4.506716 3.245793
H 6.438833 2.169356 4.103132
C 1.844660 -4.287507 -0.836256
C 2.236532 -4.910714 -2.191016
C 3.104258 -3.656099 -0.280560
C 2.672006 -3.844953 -3.207965
H 3.066161 -5.600564 -2.003466
C 3.936117 -2.946040 -1.162082
C 3.453987 -2.669189 -2.564921
H 3.282077 -4.323355 -3.979680
H 2.796346 -1.791881 -2.509852
C -0.572695 -4.015186 -1.592546
H -0.416523 -4.251431 -2.648363
H -0.809530 -4.950509 -1.072559
H -1.433671 -3.345855 -1.528098
C -1.064626 0.338089 3.290319
H -0.014523 0.585209 3.122394
H -1.590369 1.246205 3.596136
H -1.115040 -0.385490 4.109069
C -3.201538 -0.500336 2.130356
C -3.521039 -1.729053 2.958918
C -3.940963 -0.558348 0.787805
C -3.849546 -2.951161 2.350657
C -3.705987 -1.859697 0.027531
H -3.660971 0.299913 0.172475
C -4.109310 -3.075420 0.861981
H -4.275282 -1.847081 -0.904872
H -3.620391 -3.978499 0.476644
C 3.531578 -3.884541 1.025542
H 2.902184 -4.460503 1.699505
C 5.181277 -2.505573 -0.716816
C 4.767840 -3.421483 1.469930
C 5.600158 -2.735804 0.591709
C -3.455523 -1.666809 4.356581
H -3.261161 -0.707846 4.829048
C -4.011326 -4.084661 3.154246
C -3.884091 -4.022856 4.535746
H -4.008759 -4.919381 5.135239
C -3.624738 -2.797266 5.144927
H -3.558074 -2.723291 6.225947
H -4.251675 -5.032937 2.678501
H 5.086191 -3.615038 2.489790
H 2.808332 1.084888 -1.309782
H 4.641485 -0.305835 0.519073
H 4.389071 2.264891 -2.669915
H 3.312028 3.487148 -0.763206
H -1.774852 5.703785 -1.821824
H -2.504033 3.566243 -2.707568
H -3.662414 2.034299 -1.508656
H -5.014914 -0.450710 0.991781
H -2.655522 -1.926319 -0.262460
H -5.187716 -3.249329 0.744052
H 1.541096 -5.101630 -0.164296
H 1.419659 -5.511237 -2.601388
H 1.791945 -3.441679 -3.721175
H 4.299006 -2.377865 -3.196317
H -1.089468 5.172284 0.497028
H -3.585958 0.373995 2.666607
H 5.379584 0.394112 2.754951
H 1.766685 -0.749899 2.741346
C -0.210531 0.283386 -3.369942
C 1.028642 0.487751 -4.207077
H 0.825115 0.205398 -5.245878
H 1.352051 1.532421 -4.183882
H 1.837349 -0.129447 -3.812661
C -2.417761 0.376686 -3.787858
C -2.170900 -0.942220 -3.376899
C -3.677080 0.849978 -4.106242
C -3.229675 -1.841577 -3.368026
C -4.734590 -0.064894 -4.059669
H -3.825806 1.882610 -4.404389
C -4.510328 -1.395415 -3.712076
H -3.070714 -2.878036 -3.084875
H -5.736467 0.264978 -4.316396
H -5.340894 -2.094890 -3.702723
C -0.742147 -1.022550 -3.042242
O -1.273734 1.111849 -3.840162
H 1.376793 -0.475937 -1.461034
H 0.320382 1.303352 -2.149501
H -1.649631 0.948988 -1.095134
H -1.847213 0.157973 -0.942152
H -0.174178 -1.912181 -3.294384
H 5.832132 -1.971930 -1.405205
H 6.573458 -2.383562 0.919422

149
int$^{1}_{cis}$(si)
Ru -0.161106 -0.062830 1.062036
C 0.681849 -1.378524 -0.310663
C 1.858747 -2.291434 -2.116690
C 1.141646 -3.413843 -1.392664
H 1.337195 -2.023803 -3.045691
H 2.894929 -2.523425 -2.352440
H 0.600879 -4.075426 -2.071471
H 1.844633 -4.021335 -0.805236
C -0.311748 1.405988 -0.277999
C -0.201639 3.553319 -1.209930
C -0.557971 2.567543 -2.297933
H -1.089456 4.122362 -0.894778
H 0.575571 4.256668 -1.513877
H -1.365090 2.911068 -2.940070
H 0.316212 2.338552 -2.922954
N 0.252560 2.658819 -0.150231
N -0.929697 1.400962 -1.496502
N 0.236453 -2.665314 -0.522180
N 1.733449 -1.202770 -1.144704
C -1.701383 0.308781 -2.078653
H -1.598474 -0.536362 -1.397975
C 0.544287 3.229066 1.161288
H 0.851463 2.375565 1.761565

C 2.658205 -0.076227 -1.133433
H 2.218651 0.656284 -0.454218
C -0.496294 -3.393257 0.510531
H -1.075241 -2.639307 1.041936
C -1.491154 -4.405766 -0.092224
C -2.498255 -3.747246 -1.012155
C -2.277135 -5.145356 1.004688
C -3.668493 -3.202852 -0.463466
C -3.084226 -4.171066 1.867604
H -2.951105 -5.854301 0.510778
C -3.885143 -3.139001 1.033766
H -3.766370 -4.740996 2.505468
H -4.956918 -3.240816 1.231320
C 4.037177 -0.457322 -0.493899
C 3.835215 -0.792543 1.003196
C 4.817270 -1.516022 -1.257493
C 4.708460 -1.928005 1.524024
H 3.989628 0.116662 1.590066
C 4.963931 -2.825224 -0.767829
C 4.506754 -3.152792 0.633492
H 5.769933 -1.649814 1.519651
H 5.060093 -4.020435 1.007614
C 2.750768 0.560315 -2.515748
H 3.110257 -0.135716 -3.279239
H 3.423152 1.421763 -2.485724
C 0.465654 -4.028614 1.524335
H 0.935603 -4.939413 1.137058
H 1.254875 -3.314291 1.780570
H -0.058959 -4.284265 2.447843
C -2.330743 -3.751588 -2.398154
H -1.434508 -4.196071 -2.822653
C -4.648174 -2.701431 -1.324697
C -4.473501 -2.708507 -2.703109
H -5.250552 -2.314238 -3.350973
C -3.302117 -3.231298 -3.245154
H -3.155856 -3.253546 -4.320901
H -5.562904 -2.295054 -0.900603
C 5.409305 -1.194485 -2.484100
C 5.605307 -3.785703 -1.551727
H 5.713151 -4.795372 -1.162580
C 6.122869 -3.468904 -2.802607
C 6.043539 -2.157254 -3.260920
H 6.614626 -4.231334 -3.398751
H 6.482337 -1.881774 -4.215011
C 1.698496 4.249063 1.097524
C 2.025700 4.818737 2.491524
C 2.991945 3.681029 0.551635
C 2.468102 3.722656 3.472058
H 2.834534 5.546262 2.364410
C 3.823283 2.961816 1.425707
C 3.315464 2.608720 2.802708
H 3.033169 4.185444 4.286489
H 2.694684 1.709306 2.694120
C -0.722323 3.822401 1.790122
H -0.588946 3.981145 2.863524
H -1.000971 4.782532 1.340537
H -1.546426 3.116697 1.663230
C -1.088393 -0.122929 -3.411070
H -0.041174 -0.394173 -3.255554
H -1.617641 -0.998007 -3.797494
H -1.126418 0.667377 -4.166498
C -3.236765 0.602300 -2.186738
C -3.561022 1.899920 -2.899848
C -3.977435 0.538158 -0.844231
C -3.890694 3.060733 -2.181820
C -3.741541 1.764304 0.032213
H -3.698823 -0.372484 -0.310481
C -4.149857 3.049743 -0.687442
H -4.304057 1.666296 0.965020
H -3.661183 3.914859 -0.222824
C 3.438869 3.962319 -0.737633
H 2.805591 4.539460 -1.406744
C 5.090767 2.572986 0.994264
C 4.696526 3.548692 -1.169924
C 5.530969 2.859927 -0.295678
C -3.493122 1.965883 -4.297059
H -3.293753 1.054816 -4.854714
C -4.053134 4.262730 -2.878677
C -3.925180 4.327657 -4.259996
H -4.050456 5.275213 -4.774899
C -3.663399 3.163474 -4.978811
H -3.594867 3.189215 -6.061942
H -4.294055 5.163474 -2.318397
H 5.030094 3.784005 -2.176143
H 2.792070 -1.073102 1.170328
H 4.620535 0.466688 -0.560414
H 4.435475 -2.154914 2.560323
H 3.442260 -3.425155 0.647339
H -1.606921 -5.737414 1.635303
H -2.409808 -3.638061 2.546014
H -3.625887 -2.129769 1.379127
H -5.051365 0.451169 -1.058698
H -2.689432 1.807937 0.318464
H -5.228408 3.210274 -0.553027
H 1.389673 5.085553 0.456121
H 1.173861 5.368637 2.902075
H 1.587041 3.263091 3.933138
H 4.155188 2.325058 3.444719
H -0.924318 -5.148627 -0.669610
H -3.617262 -0.223169 -2.798982
H 5.369732 -0.167401 -2.834665
H 1.761065 0.910898 -2.817016
C -0.183778 -0.757824 3.314004
C 1.007358 -0.905876 4.242797
H 0.743918 -0.548833 5.242014
H 1.327446 -1.950047 4.312938
H 1.835073 -0.309720 3.850157
C -2.399139 -0.806037 3.791318
C -2.136730 0.526071 3.443394
C -3.654658 -1.270368 4.133804
C -3.182131 1.438757 3.506850
C -4.699929 -0.339294 4.160692
H -3.811337 -2.313342 4.388645
C -4.462684 0.999578 3.863704
H -3.011972 2.484240 3.267464
H -5.698936 -0.666275 4.431663
H -5.280179 1.713116 3.906764
C -0.717041 0.628719 3.037600
O -1.275745 -1.584227 3.759482
H 1.305319 0.495622 1.397842
H 0.181694 -1.318882 2.343496
H -1.657749 -0.891099 0.577149
H -1.910905 -0.224330 1.010258
H -0.149879 1.440549 3.497102
H 5.741692 2.033935 1.678440
H 6.521301 2.547802 -0.612991

149

TS$^{PT}_{cis}$(si)

Ru -0.150906 -0.055224 1.110193
C 0.687624 -1.385538 -0.282819
C 1.842692 -2.296385 -2.097854
C 1.037098 -3.397112 -1.431659
H 1.368227 -1.970057 -3.032295
H 2.869754 -2.581670 -2.311640
H 0.456815 -3.982845 -2.146222
H 1.682814 -4.083242 -0.865133
C -0.202801 1.413104 -0.233789
C -0.149476 3.593224 -1.086950
C -0.442689 2.630888 -2.217465
H -1.061570 4.124022 -0.773746
H 0.611904 4.330659 -1.345371
H -1.260870 2.954424 -2.856318
H 0.448584 2.469730 -2.839259
N 0.305801 2.677785 -0.045406
N -0.767123 1.414617 -1.471115
N 0.176827 -2.628626 -0.534828
N 1.753841 -1.229315 -1.095911
C -1.583276 0.360982 -2.066174
H -1.519280 -0.482228 -1.377711
C 0.625938 3.204123 1.275793
H 0.935097 2.321742 1.837874
C 2.706540 -0.124638 -1.060881
H 2.293528 0.589328 -0.345025
C -0.635764 -3.333490 0.453061
H -1.194436 -2.542534 0.955364
C -1.648154 -4.290605 -0.205348
C -2.611669 -3.587319 -1.138791
C -2.500194 -5.018741 0.851207
C -3.745632 -2.968502 -0.588753
C -3.297526 -4.034311 1.716293
H -3.186266 -5.683927 0.315517
C -3.896773 -2.852395 0.909600
H -4.094702 -4.582181 2.228088
H -4.952883 -2.712565 1.158653
C 4.088225 -0.553396 -0.461043
C 3.911981 -0.915193 1.034660
C 4.828375 -1.609065 -1.265851
C 4.712622 -2.127239 1.498658
H 4.156199 -0.037327 1.639295
C 4.923153 -2.940157 -0.825153
C 4.433467 -3.297934 0.557030
H 5.789776 -1.918124 1.500002
H 4.922099 -4.216986 0.896782
C 2.790818 0.554417 -2.423260
H 3.132516 -0.123043 -3.211631
H 3.476177 1.404415 -2.374554
C 0.258936 -4.036880 1.484232
H 0.684712 -4.969851 1.097232
H 1.085851 -3.378060 1.770111
H -0.304235 -4.271739 2.390556
C -2.464071 -3.639121 -2.524149
H -1.599172 -4.142604 -2.949036
C -4.712300 -2.443788 -1.446953
C -4.558882 -2.500224 -2.828791
H -5.324476 -2.084850 -3.477205

C -3.422812 -3.092545 -3.372102
H -3.294042 -3.149878 -4.448815
H -5.598260 -1.979517 -1.021579
C 5.429094 -1.264753 -2.481273
C 5.534282 -3.893667 -1.640293
H 5.603539 -4.920324 -1.288720
C 6.067352 -3.549517 -2.877877
C 6.031370 -2.221284 -3.291142
H 6.534961 -4.306525 -3.499832
H 6.478798 -1.928380 -4.235986
C 1.794829 4.210162 1.222556
C 2.166786 4.720901 2.627946
C 3.064719 3.645856 0.619997
C 2.615690 3.580797 3.552565
H 2.984654 5.439384 2.507230
C 3.913535 2.888115 1.443153
C 3.450019 2.494259 2.824234
H 3.190502 4.005096 4.381136
H 2.831214 1.594587 2.702386
C -0.618664 3.803908 1.943447
H -0.456159 3.949440 3.014840
H -0.894287 4.773417 1.512610
H -1.462811 3.120094 1.826965
C -0.983505 -0.072778 -3.402076
H 0.044100 -0.406975 -3.243050
H -1.557066 -0.906245 -3.817171
H -0.963721 0.735980 -4.138909
C -3.109545 0.702828 -2.154081
C -3.448841 1.945825 -2.958368
C -3.799037 0.732638 -0.782026
C -3.842634 3.142911 -2.336716
C -3.598975 2.050293 -0.040178
H -3.432174 -0.093029 -0.170325
C -4.136421 3.225394 -0.851686
H -4.097419 2.007060 0.933819
H -3.759316 4.175193 -0.453071
C 3.474687 3.965793 -0.672693
H 2.829739 4.575183 -1.300816
C 5.159188 2.494497 0.955638
C 4.710833 3.549395 -1.160227
C 5.561053 2.817057 -0.338271
C -3.356617 1.916661 -4.356318
H -3.117361 0.976477 -4.844781
C -4.038770 4.283931 -3.121840
C -3.885072 4.255044 -4.501475
H -4.038408 5.156570 -5.086697
C -3.560979 3.053483 -5.126752
H -3.472627 3.002010 -6.207616
H -4.328744 5.211260 -2.632651
H 5.015176 3.814448 -2.168256
H 2.855181 -1.113580 1.231250
H 4.689273 0.359415 -0.522224
H 4.429738 -2.381102 2.526028
H 3.352022 -3.494361 0.552623
H -1.877937 -5.657070 1.485981
H -2.649126 -3.628568 2.499787
H -3.392691 -1.927148 1.217906
H -4.874807 0.574433 -0.938830
H -2.536336 2.190982 0.161642
H -5.229084 3.267560 -0.741583
H 1.482204 5.075263 0.621923
H 1.336358 5.269462 3.082175
H 1.736997 3.105308 4.002826
H 4.311576 2.199055 3.431318
H -1.094765 -5.047576 -0.778151
H -3.520754 -0.156812 -2.694390
H 5.421300 -0.225314 -2.796623
H 1.802444 0.926893 -2.701247
C -0.294085 -0.873701 3.335748
C 0.873854 -0.984533 4.298814
H 0.569390 -0.643670 5.292179
H 1.216792 -2.020388 4.372733
H 1.692647 -0.364887 3.927234
C -2.496380 -0.935086 3.873124
C -2.262227 0.399451 3.547856
C -3.742926 -1.404420 4.254677
C -3.302070 1.313332 3.631740
C -4.782259 -0.474865 4.324575
H -3.894609 -2.451583 4.491971
C -4.567166 0.869911 4.025680
H -3.137133 2.357860 3.382281
H -5.771427 -0.807422 4.623655
H -5.388533 1.575828 4.094915
C -0.843324 0.524963 3.086408
O -1.380431 -1.709117 3.781804
H 1.379734 0.454984 1.603387
H 0.067078 -1.410200 2.374472
H -1.614454 -0.688262 0.645175
H -1.419593 0.742359 1.628817
H -0.279133 1.329512 3.558535
H 5.824393 1.924477 1.599856
H 6.535242 2.500529 -0.698484

149

1-C_{cis}-bf(re)

Ru 0.006871 -0.099767 0.573280
C 0.597284 1.740501 -0.187176
C 0.650198 3.771194 -1.368878
C 1.804877 3.738957 -0.395538
H 0.985938 3.605811 -2.402184
H 0.089183 4.707343 -1.336858
H 2.740573 4.071410 -0.840055
H 1.599925 4.361318 0.487046
C -0.295204 -0.935774 -1.404664
C -1.100768 -2.234630 -3.192658
C 0.126060 -1.479365 -3.652743
H -0.880666 -3.296278 -3.016387
H -1.930779 -2.172435 -3.897881
H 0.842856 -2.110968 -4.181922
H -0.140556 -0.635164 -4.305804
N -1.387721 -1.558379 -1.925533
N 0.655769 -0.995861 -2.381162
N 1.830105 2.322298 -0.025648
N -0.139111 2.637437 -0.900982
C 1.968069 -0.361341 -2.360768
H 2.089169 -0.012646 -1.337771
C -2.658769 -1.774136 -1.238780
H -2.427649 -1.722658 -0.176371
C -1.545724 2.552000 -1.265220
H -1.872888 1.579751 -0.905397
C 2.880319 1.830273 0.870015
H 2.464972 0.913382 1.280603
C 4.172694 1.434565 0.103339
C 5.156648 0.739834 1.036679
C 4.807547 2.582873 -0.700092
C 6.493490 1.139868 1.172790
C 6.287745 2.359406 -0.995595
H 4.249022 2.719754 -1.633033
C 7.050604 2.258757 0.322945
H 6.426672 1.435498 -1.572422
H 8.117543 2.087212 0.146006
C -2.360255 3.659907 -0.506982
C -3.698637 4.023067 -1.163101
C -2.560958 3.221420 0.934648
C -4.731829 2.913184 -0.986321
H -4.074470 4.935086 -0.682050
C -3.771414 2.645739 1.353227
C -5.019250 2.689991 0.494652
H -5.658926 3.158708 -1.514548
H -5.641322 3.519555 0.860135
C -1.694989 2.566630 -2.786893
H -1.371689 3.514914 -3.230055
H -2.731961 2.397973 -3.081331
C 3.115482 2.787306 2.037693
H 3.532383 3.753483 1.734260
H 2.170425 2.967225 2.559786
H 3.814494 2.331930 2.743037
C 4.715023 -0.381934 1.743257
H 3.706282 -0.740631 1.575618
C 7.328030 0.446130 2.053529
C 6.862475 -0.636309 2.789245
H 7.528020 -1.159216 3.469266
C 5.547434 -1.061498 2.620764
H 5.171980 -1.930609 3.152054
H 8.365303 0.758371 2.149320
C -1.499208 3.299625 1.839730
C -3.846709 2.084764 2.631180
H -4.775388 1.611270 2.939449
C -2.764698 2.117549 3.501542
C -1.589349 2.751399 3.111996
H -2.837480 1.653834 4.479638
H -0.735502 2.784261 3.780239
C -3.650539 -0.612458 -1.527992
C -3.942291 -0.332400 -3.009863
C -4.922417 -0.737925 -0.702789
C -5.114596 -1.140578 -3.561493
H -4.207043 0.727354 -3.114634
C -6.193056 -0.803504 -1.294174
C -6.380225 -0.765102 -2.796623
H -5.248010 -0.936245 -4.629047
H -7.211943 -1.419942 -3.078238
C -3.206748 -3.172059 -1.516617
H -4.123587 -3.318156 -0.941454
H -3.439069 -3.342318 -2.570888
H -2.482753 -3.931433 -1.207007
C 1.946552 0.855891 -3.293955
H 1.042086 1.433231 -3.089727
H 2.803597 1.508695 -3.118360
H 1.947510 0.576786 -4.353690
C 3.123351 -1.367118 -2.664440
C 3.390533 -2.328121 -1.500273
C 4.387555 -0.603001 -3.109196
C 4.670270 -2.488182 -0.945488
C 5.660912 -1.427408 -2.980166
H 4.515973 0.295306 -2.494510
C 5.875779 -1.777054 -1.512918

H 5.582077 -2.346514 -3.574955
H 6.067740 -0.861200 -0.937567
C -4.824819 -0.753761 0.694078
H -3.850533 -0.694811 1.171842
C -7.324889 -0.871284 -0.475760
H -8.306487 -0.919373 -0.941696
C -5.956184 -0.823299 1.495031
C -7.217223 -0.878606 0.907874
C 2.348874 -3.113400 -0.985162
H 1.360372 -3.032648 -1.417837
C 4.853359 -3.358340 0.134215
C 3.802989 -4.091135 0.662611
H 3.967025 -4.759636 1.502142
C 2.542680 -3.979110 0.081363
H 1.710553 -4.565056 0.459969
H 5.848497 -3.451689 0.561235
H -8.109637 -0.932137 1.523983
H -5.842349 -0.837550 2.573380
H -3.556239 4.270128 -2.219379
H -1.747341 4.569173 -0.507017
H -4.359388 1.983682 -1.426916
H -5.605130 1.778112 0.637596
H 4.727513 3.523433 -0.139860
H 6.678152 3.181225 -1.604836
H 6.973220 3.217201 0.855433
H 4.254300 -0.254543 -4.137981
H 6.514107 -0.861810 -3.369949
H 6.760854 -2.409125 -1.386704
H -3.122450 0.263894 -1.141150
H -3.033858 -0.464038 -3.603698
H -4.930278 -2.215002 -3.463671
H -6.679472 0.252164 -3.086818
H 3.852500 0.666096 -0.608122
H 2.818576 -1.984026 -3.522523
H -0.569165 3.763667 1.524960
H -1.095058 1.758230 -3.215386
C 0.198735 -1.236058 2.383808
C 1.567374 -1.469143 2.965490
H 2.041465 -0.538719 3.287792
H 1.479760 -2.136375 3.831344
H 2.196291 -1.950859 2.214879
C -1.751961 -2.420668 2.080098
C -1.852698 -1.599195 3.213463
C -2.764126 -3.343537 1.836332
C -2.963194 -1.588856 4.041548
C -3.875239 -3.382447 2.681069
H -2.689030 -4.036892 1.005656
C -3.979031 -2.505741 3.760416
H -3.006701 -0.932787 4.903956
H -4.670138 -4.096156 2.489814
H -4.852438 -2.546793 4.404606
H 0.516932 0.743229 1.846810
H 1.514756 -0.593486 0.379088
C -0.449558 -2.127285 1.470195
O -0.720642 -0.891578 3.419505
H 0.107917 -2.887535 0.937706
H -1.746963 0.223462 0.642340
H -1.420965 0.623769 1.304600

149

TS$^{HT}_{cis}$(re)

Ru -0.038399 -0.046680 0.521807
C 0.559462 1.696564 -0.430042
C 0.624914 3.587629 -1.830403
C 1.787359 3.650812 -0.865586
H 0.953718 3.314371 -2.843051
H 0.071309 4.527192 -1.893074
H 2.722834 3.915400 -1.354314
H 1.600319 4.376572 -0.061242
C -0.286930 -1.113962 -1.285462
C -1.051398 -2.705526 -2.839770
C 0.164749 -2.008017 -3.411402
H -0.810484 -3.720705 -2.496311
H -1.877761 -2.773479 -3.549452
H 0.894845 -2.703571 -3.830847
H -0.114340 -1.285311 -4.192918
N -1.361438 -1.842378 -1.699769
N 0.677527 -1.318572 -2.232874
N 1.796480 2.287595 -0.332704
N -0.169492 2.515522 -1.243660
C 1.970130 -0.646726 -2.302132
H 2.080419 -0.171284 -1.330524
C -2.623614 -1.979018 -0.977145
H -2.385534 -1.780347 0.067174
C -1.568075 2.377527 -1.623154
H -1.897056 1.447388 -1.166668
C 2.829667 1.888828 0.624505
H 2.412045 1.008420 1.107574
C 4.140889 1.427843 -0.073996
C 5.087953 0.809634 0.945704
C 4.805384 2.508170 -0.947786
C 6.396385 1.270023 1.149369
C 6.307838 2.303295 -1.117386
H 4.311018 2.534269 -1.925626
C 6.973729 2.343786 0.256323
H 6.510078 1.335822 -1.595807
H 8.056765 2.205907 0.171913
C -2.403998 3.555917 -1.011132
C -3.755526 3.794578 -1.696465
C -2.585679 3.307553 0.476877
C -4.752655 2.685571 -1.370522
H -4.156332 4.748868 -1.331560
C -3.776494 2.757782 0.977524
C -5.027328 2.647222 0.129233
H -5.689302 2.831981 -1.918259
H -5.676588 3.495695 0.388562
C -1.696479 2.224344 -3.139586
H -1.393381 3.128257 -3.679356
H -2.725401 1.995788 -3.423252
C 3.038360 2.948161 1.706026
H 3.462132 3.883597 1.325662
H 2.080811 3.174719 2.185958
H 3.720794 2.560808 2.466509
C 4.636478 -0.286184 1.685450
H 3.662182 -0.705851 1.454093
C 7.185097 0.673412 2.136304
C 6.700741 -0.372684 2.911827
H 7.328283 -0.818908 3.677283
C 5.421882 -0.867000 2.671009
H 5.041992 -1.717705 3.227869
H 8.200655 1.032465 2.285839
C -1.521763 3.534823 1.354732
C -3.830316 2.370738 2.319926
H -4.741967 1.912637 2.695346
C -2.747337 2.553173 3.169510
C -1.591760 3.161057 2.689507
H -2.800955 2.223303 4.201406
H -0.739569 3.312534 3.343810
C -3.627598 -0.878616 -1.423410
C -3.895301 -0.797591 -2.933820
C -4.910410 -0.909865 -0.607052
C -5.057159 -1.676283 -3.390601
H -4.157265 0.238481 -3.183414
C -6.171835 -1.050298 -1.205022
C -6.334955 -1.205740 -2.702646
H -5.173892 -1.618655 -4.477924
H -7.162877 -1.892304 -2.910302
C -3.156646 -3.408239 -1.042097
H -4.063485 -3.476843 -0.437234
H -3.400825 -3.734950 -2.056266
H -2.420386 -4.105454 -0.631664
C 1.908202 0.450657 -3.371247
H 0.995792 1.029702 -3.215636
H 2.753866 1.136514 -3.289144
H 1.895319 0.049661 -4.391055
C 3.155319 -1.645468 -2.487115
C 3.488105 -2.434168 -1.212192
C 4.384037 -0.919482 -3.070069
C 4.807771 -2.580043 -0.752820
C 5.665351 -1.732502 -2.946738
H 4.542406 0.031574 -2.547269
C 5.976010 -1.948875 -1.471882
H 5.548337 -2.701747 -3.448611
H 6.207402 -0.983995 -0.997530
C -4.834367 -0.755593 0.783038
H -3.867633 -0.642399 1.266540
C -7.316531 -1.015293 -0.402527
H -8.290748 -1.121761 -0.874260
C -5.978512 -0.724026 1.568125
C -7.230652 -0.849321 0.972466
C 2.470515 -3.081955 -0.496350
H 1.446802 -2.995311 -0.832369
C 5.063240 -3.330232 0.398987
C 4.042338 -3.938015 1.111850
H 4.263593 -4.511846 2.006705
C 2.734338 -3.814806 0.652541
H 1.917544 -4.294296 1.183824
H 6.090444 -3.418220 0.743256
H -8.132661 -0.824340 1.576297
H -5.881827 -0.603952 2.641457
H -3.626303 3.909469 -2.776654
H -1.813670 4.471390 -1.139058
H -4.352465 1.719134 -1.691600
H -5.581139 1.742635 0.393910
H 4.665377 3.497732 -0.494681
H 6.721308 3.079148 -1.770163
H 6.819164 3.337550 0.699950
H 4.190155 -0.667980 -4.117056
H 6.490858 -1.209861 -3.441615
H 6.865053 -2.575436 -1.345673
H -3.115319 0.048452 -1.148636
H -2.976516 -1.006149 -3.488255
H -4.871990 -2.727035 -3.145111
H -6.627825 -0.234930 -3.127325
H 3.836697 0.606419 -0.729671
H 2.857501 -2.386387 -3.243917

H -0.608385 3.980556 0.971834
H -1.071562 1.391534 -3.474275
C 0.159137 -0.629048 2.631239
C 1.494668 -0.687145 3.335384
H 1.905736 0.305124 3.542534
H 1.370477 -1.223477 4.281780
H 2.198412 -1.236342 2.710460
C -1.664759 -2.042046 2.476669
C -1.908042 -1.031184 3.418014
C -2.592952 -3.069329 2.349277
C -3.061195 -0.969763 4.179559
C -3.753349 -3.046983 3.128264
H -2.413817 -3.888193 1.659490
C -3.986403 -2.007841 4.026187
H -3.210542 -0.174804 4.901938
H -4.482040 -3.845246 3.029256
H -4.891696 -2.006844 4.625907
H 0.401168 0.751422 1.885013
H 1.469898 -0.567990 0.429813
C -0.353544 -1.761735 1.878115
O -0.853520 -0.182398 3.530056
H 0.314068 -2.580488 1.631915
H -1.805537 0.178467 0.560198
H -1.511240 0.830422 0.984758

149

int^{1}_{cis}(re)

Ru 0.219963 -0.520376 -0.324289
C 0.983076 -0.943472 1.570252
C 1.320057 -1.062999 3.902783
C 2.474187 -1.764819 3.206745
H 1.637523 -0.116354 4.360142
H 0.861800 -1.673827 4.685417
H 2.435071 -2.855599 3.339996
H 3.443711 -1.414349 3.563734
C -0.155546 1.352954 0.174937
C -1.204897 3.447035 0.399873
C 0.062251 3.443016 1.223912
H -1.067022 4.025293 -0.524988
H -2.058512 3.855587 0.939948
H 0.654099 4.348613 1.100550
H -0.159986 3.311191 2.294074
N -1.360849 2.021577 0.105268
N 0.731716 2.261723 0.693109
N 2.250285 -1.403442 1.811690
N 0.403992 -0.814326 2.795263
C 2.170994 2.105252 0.797789
H 2.361631 1.064929 0.539550
C -2.515915 1.550723 -0.662699
H -2.160667 0.679567 -1.211599
C -0.972095 -0.443902 3.088506
H -1.400691 -0.118720 2.139120
C 3.125509 -1.924074 0.770492
H 2.761789 -1.434732 -0.132188
C 4.602736 -1.459416 0.942745
C 5.246168 -1.485632 -0.436145
C 5.413826 -2.223289 2.027567
C 6.144853 -2.486157 -0.821527
C 6.773445 -2.744030 1.554731
H 5.562148 -1.576119 2.898090
C 6.631693 -3.467928 0.215630
H 7.476596 -1.911833 1.431250
H 7.585642 -3.902495 -0.099505
C -1.748236 -1.709525 3.595878
C -3.033212 -1.391784 4.366854
C -2.040149 -2.613999 2.411946
C -4.094973 -0.814014 3.438086
H -3.406623 -2.322612 4.811777
C -3.282183 -2.562009 1.761011
C -4.465844 -1.814828 2.343914
H -4.989828 -0.531919 4.002721
H -5.149330 -2.562596 2.769255
C -0.998696 0.749787 4.045093
H -0.677202 0.483599 5.058043
H -2.003572 1.172848 4.115042
C 2.950309 -3.430938 0.573785
H 3.212978 -4.018867 1.458719
H 1.905506 -3.642818 0.327804
H 3.573768 -3.773098 -0.257338
C 4.877330 -0.509013 -1.364060
C 6.623910 -2.506491 -2.133565
C 6.225792 -1.548838 -3.058682
H 6.606437 -1.583104 -4.074660
C 5.354166 -0.534612 -2.667543
H 7.326299 -3.282899 -2.426680
C -1.035486 -3.437232 1.893025
C -3.452240 -3.270543 0.567384
H -4.405710 -3.202543 0.050662
C -2.425170 -4.031835 0.026116
C -1.215552 -4.136438 0.707358
H -2.562957 -4.539233 -0.922509
H -0.410619 -4.740616 0.300617
C -3.662531 1.094602 0.277939
C -4.107444 2.217679 1.235275
C -4.813519 0.446163 -0.484803
C -5.522992 2.046550 1.777258
H -3.380898 2.294080 2.052498
C -6.147278 0.856493 -0.322865
C -6.499150 1.993811 0.607293
H -5.609791 1.123275 2.359632
H -6.463489 2.949142 0.064644
C -2.957665 2.566577 -1.716065
H -3.722870 2.108870 -2.347390
H -3.373762 3.485156 -1.290305
H -2.110821 2.839562 -2.353082
C 2.625107 2.301723 2.243685
H 2.113350 1.568338 2.874471
H 3.703811 2.138244 2.334535
H 2.401912 3.300538 2.629933
C 2.966193 2.942353 -0.260328
C 2.807255 4.447622 -0.122324
C 2.691921 2.484940 -1.707100
C 2.025155 5.194875 -1.018386
C 1.418890 3.073700 -2.312695
H 2.621848 1.395561 -1.745314
C 1.451274 4.599128 -2.288120
H 1.313408 2.725545 -3.345776
H 0.450353 5.009317 -2.467713
C -4.547060 -0.646973 -1.317791
H -3.526527 -0.992756 -1.458720
C -7.165538 0.173323 -0.991579
H -8.194350 0.495513 -0.849148
C -5.569478 -1.323742 -1.971032
C -6.888046 -0.911402 -1.812103
C 3.430305 5.115197 0.940423
H 4.085497 4.553631 1.600183
C 1.825547 6.557568 -0.772004
C 2.405127 7.194865 0.316454
H 2.230202 8.252779 0.486095
C 3.232406 6.469731 1.170451
H 3.723074 6.957378 2.007093
H 1.207159 7.127488 -1.461815
H -7.694183 -1.436051 -2.315829
H -5.323548 -2.168643 -2.603998
H -2.827370 -0.710339 5.197880
H -1.078901 -2.242273 4.282530
H -3.710225 0.106804 2.986718
H -5.022006 -1.319647 1.540416
H 4.838343 -3.083782 2.384707
H 7.192413 -3.410420 2.315945
H 5.925022 -4.302406 0.326020
H 3.548662 2.787342 -2.324628
H 0.548090 2.695318 -1.775624
H 2.073940 4.957392 -3.119801
H -3.221039 0.293490 0.885674
H -4.088229 3.180254 0.709745
H -5.767397 2.876736 2.448551
H -7.528727 1.875173 0.960483
H 4.532367 -0.404877 1.237545
H 4.017609 2.705807 -0.042257
H -0.081630 -3.496305 2.410510
H -0.330503 1.527721 3.663706
C 0.542777 -2.060647 -2.133845
C 1.865901 -2.536474 -2.708142
H 2.102481 -3.551579 -2.375952
H 1.806523 -2.529999 -3.800036
H 2.670210 -1.866006 -2.395210
C -1.169440 -0.870908 -3.199561
C -1.465116 -2.239779 -3.169451
C -2.016942 -0.022492 -3.899797
C -2.579147 -2.784170 -3.780917
C -3.144289 -0.548363 -4.540977
H -1.799181 1.040379 -3.954002
C -3.419634 -1.910826 -4.483408
H -2.764465 -3.852847 -3.750176
H -3.810136 0.112520 -5.087047
H -4.294946 -2.306537 -4.989680
H 0.635238 -2.247384 -0.980934
H 1.689683 -0.049224 -0.717790
C 0.089127 -0.650338 -2.459101
O -0.507815 -2.964151 -2.524288
H 0.824786 -0.044426 -2.989265
H -1.495514 -0.942229 -0.401558
H -1.265024 -1.226726 0.349076
H 4.188597 0.272326 -1.056713
H 5.045895 0.232088 -3.371630

149

TS^{PT}_{cis}(re)

Ru 0.386617 0.095055 0.710532
C -0.367703 -1.731544 0.325849
C -0.703546 -3.919135 -0.513848
C -1.772922 -3.624115 0.530417
H -1.121606 -4.076969 -1.515257
H -0.125050 -4.813800 -0.263348
H -2.785666 -3.760398 0.140294

H -1.658500 -4.272955 1.404472
C 0.713169 1.089743 -1.176702
C 1.529528 2.722644 -2.673392
C 0.103510 2.364973 -3.055593
H 1.655459 3.799883 -2.549167
H 2.262133 2.366548 -3.408637
H -0.584460 3.170774 -2.774977
H -0.011876 2.161637 -4.121422
N 1.680910 2.019363 -1.399017
N -0.145424 1.181898 -2.232841
N -1.520786 -2.228263 0.878365
N 0.123263 -2.720257 -0.478655
C -1.073586 0.128428 -2.621530
H -0.774831 -0.703094 -1.985079
C 2.907191 2.155011 -0.614506
H 2.618453 2.022318 0.421502
C 1.375005 -2.712112 -1.224017
H 1.764191 -1.699534 -1.107361
C -2.232289 -1.617034 2.008071
H -1.490859 -0.934300 2.422977
C -3.449866 -0.775710 1.563398
C -4.052503 0.063707 2.693895
C -4.530521 -1.566741 0.822937
C -5.402259 0.455930 2.652701
C -5.644289 -0.628950 0.369578
H -4.950863 -2.346448 1.473179
C -6.353681 -0.027356 1.576254
H -5.202307 0.167857 -0.242211
H -7.007799 0.797003 1.270191
C 2.387992 -3.730769 -0.610534
C 3.641881 -3.916704 -1.475178
C 2.771665 -3.351898 0.808994
C 4.537397 -2.684446 -1.405306
H 4.197461 -4.782512 -1.092954
C 3.993328 -2.724041 1.086504
C 5.047330 -2.492205 0.022020
H 5.383657 -2.783390 -2.093606
H 5.866164 -3.203729 0.198830
C 1.104820 -2.994084 -2.706091
H 0.939528 -4.060498 -2.899680
H 1.946401 -2.671071 -3.323684
C -2.567496 -2.650132 3.084963
H -3.284683 -3.405162 2.748274
H -1.659204 -3.157351 3.424219
H -3.010676 -2.144235 3.944883
C -3.273583 0.508300 3.772691
H -2.235585 0.202805 3.853776
C -5.910404 1.287778 3.654941
C -5.120299 1.732886 4.704702
H -5.537822 2.381268 5.468641
C -3.790290 1.331121 4.765105
H -3.152589 1.654614 5.581957
H -6.954898 1.586813 3.602986
C 1.889284 -3.609537 1.861966
C 4.278138 -2.345445 2.401199
H 5.226024 -1.855880 2.608017
C 3.376402 -2.570332 3.431531
C 2.173311 -3.215906 3.160536
H 3.615022 -2.254741 4.442831
H 1.460283 -3.409100 3.956032
C 3.935688 1.032497 -0.914784
C 4.446205 1.049713 -2.370331
C 5.032457 1.082395 0.137617
C 5.892393 0.580567 -2.513182
H 3.778637 0.442576 -2.992724
C 6.375311 1.322814 -0.182710
C 6.793679 1.442027 -1.629561
H 5.987783 -0.468243 -2.210239
H 6.727370 2.488623 -1.959673
C 3.486099 3.563934 -0.708350
H 4.332855 3.643942 -0.022674
H 3.844174 3.822332 -1.709657
H 2.733180 4.295091 -0.405405
C -0.844299 -0.275151 -4.083175
H -1.198951 0.481597 -4.788634
H 0.221386 -0.439543 -4.266537
H -1.381982 -1.198340 -4.310037
C -2.558127 0.435079 -2.306671
C -3.461133 -0.772092 -2.561842
C -3.093726 1.687842 -3.005621
C -4.791883 -0.618699 -2.980952
C -4.572317 1.890349 -2.693904
H -2.965294 1.606678 -4.093369
C -5.397850 0.740480 -3.259847
H -4.705207 1.944379 -1.606004
H -5.486191 0.863129 -4.348100
C 4.680968 0.871956 1.476647
H 3.656396 0.598272 1.718204
C 7.323475 1.386841 0.841392
H 8.365246 1.563361 0.584221
C 5.629885 0.947640 2.488013
C 6.959036 1.217655 2.171377
C -2.986062 -2.071565 -2.346383
H -1.967096 -2.208700 -2.008695
C -5.592787 -1.751660 -3.159909
C -5.104808 -3.032254 -2.945505
H -5.746359 -3.895212 -3.093154
C -3.783165 -3.191019 -2.539423
H -3.375865 -4.183310 -2.365570
H -6.624667 -1.614989 -3.475150
H 7.710333 1.273442 2.953189
H 5.333369 0.774732 3.518279
H 3.373047 -4.155097 -2.507710
H 1.880541 -4.703915 -0.577960
H 3.973697 -1.800693 -1.728101
H 5.479385 -1.494305 0.147950
H -4.092126 -2.058090 -0.046241
H -6.356870 -1.154229 -0.273751
H -7.011547 -0.787639 2.020007
H -2.529712 2.558144 -2.669828
H -4.926510 2.842926 -3.101299
H -6.419988 0.768374 -2.865621
H 3.382579 0.101943 -0.740513
H 4.401693 2.068612 -2.773526
H 6.204439 0.645352 -3.560941
H 7.843160 1.147675 -1.734801
H -3.041293 -0.061545 0.834058
H -2.581530 0.635480 -1.226046
H 0.951668 -4.118425 1.656900
H 0.220397 -2.448854 -3.040330
C 0.539817 2.133002 1.922227
C 0.801427 2.259250 3.411350
H 1.839225 2.547981 3.599262
H 0.139101 3.017414 3.838591
H 0.612621 1.292302 3.881058
C -1.308552 2.836020 0.665204
C -0.254417 3.740343 0.539131
C -2.526927 3.121798 0.072945
C -0.346313 4.891667 -0.226418
C -2.657644 4.289519 -0.684508
H -3.367359 2.444845 0.202387
C -1.573135 5.151943 -0.844101
H 0.491564 5.575255 -0.311199
H -3.608268 4.522968 -1.153974
H -1.686182 6.052926 -1.439263
H 0.317909 -0.497809 2.235784
H -1.158981 0.483344 0.441450
C -0.843488 1.693824 1.495869
O 0.834082 3.381007 1.267302
H 1.385400 1.401721 1.560303
H 1.810330 -0.691891 0.618158
H -1.507710 1.445131 2.317580
